# Genome-wide identification of microRNA and siRNA responsive to endophytic beneficial diazotrophic bacteria in maize

**DOI:** 10.1186/1471-2164-15-766

**Published:** 2014-09-06

**Authors:** Flávia Thiebaut, Cristian A Rojas, Clícia Grativol, Mariana Romeiro Motta, Tauan Vieira, Michael Regulski, Robert A Martienssen, Laurent Farinelli, Adriana S Hemerly, Paulo CG Ferreira

**Affiliations:** Laboratório de Biologia Molecular de Plantas, Instituto de Bioquímica Médica Leopoldo de Meis, Universidade Federal do Rio de Janeiro, Cidade Universitária, Avenida Carlos Chagas Filho, 373, CCS, Bl.L-29ss, Rio de Janeiro, RJ 21941-599 Brazil; Universidade Federal da INTEGRAÇÃO Latino-Americana, Av. Tancredo Neves, 6731, Bl.4, Foz do Iguaçu, PR 85867-970 Brazil; Howard Hughes Medical Institute and Gordon and Betty Moore Foundation, Cold Spring Harbor Laboratory, Cold Spring Harbor, NY 11724 USA; Fasteris SA, 1228-Plan-les-Ouates, Genève, Switzerland

**Keywords:** *Herbaspirillum seropedicae*, miRNA, siRNA, *Azospirillum brasilense*, Epigenetics

## Abstract

**Background:**

Small RNA (sRNA) has been described as a regulator of gene expression. In order to understand the role of maize sRNA (*Zea mays* – hybrid UENF 506-8) during association with endophytic nitrogen-fixing bacteria, we analyzed the sRNA regulated by its association with two diazotrophic bacteria, *Herbaspirillum seropedicae* and *Azospirillum brasilense*.

**Results:**

Deep sequencing analysis was done with RNA extracted from plants inoculated with *H. seropedicae,* allowing the identification of miRNA and siRNA. A total of 25 conserved miRNA families and 15 novel miRNAs were identified. A dynamic regulation in response to inoculation was also observed. A hypothetical model involving copper-miRNA is proposed, emphasizing the fact that the up-regulation of miR397, miR398, miR408 and miR528, which is followed by inhibition of their targets, can facilitate association with diazotrophic bacteria. Similar expression patterns were observed in samples inoculated with *A. brasilense*. Moreover, novel miRNA and siRNA were classified in the Transposable Elements (TE) database, and an enrichment of siRNA aligned with TE was observed in the inoculated samples. In addition, an increase in 24-nt siRNA mapping to genes was observed, which was correlated with an increase in methylation of the coding regions and a subsequent reduction in transcription.

**Conclusion:**

Our results show that maize has RNA-based silencing mechanisms that can trigger specific responses when plants interact with beneficial endophytic diazotrophic bacteria. Our findings suggest important roles for sRNA regulation in maize, and probably in other plants, during association with diazotrophic bacteria, emphasizing the up-regulation of Cu-miRNA.

**Electronic supplementary material:**

The online version of this article (doi:10.1186/1471-2164-15-766) contains supplementary material, which is available to authorized users.

## Background

Plants have a complex mechanism of gene expression regulation that influences their development, adaptation and response to biotic and abiotic interactions. One of these mechanisms involves sRNA and can act by silencing genes at a transcriptional or post-transcription level [1, 2]. In plants, sRNA is 20 to 24 nucleotides (nt) in length, and can be divided into two categories: microRNA (miRNA) and small interfering RNA (siRNA), both produced by RNase III-like enzymes called DCLs, or DICER-like enzymes [3, 4]. The type of precursor molecules and the enzymes involved in their biogenesis and function can differentiate these sRNA classes [5].

MiRNA is a class of sRNA derived from single-stranded precursors with self-complementary regions, forming a hairpin structure that is processed by DCL, particularly DCL1, together with a dsRNA-binding protein, HYPONASTIC LEAVES 1 - HYL1 [6]. After cleavage, a miRNA/miRNA* duplex is produced and one strand is incorporated into an RNA-induced silencing complex (RISC), where the miRNA associates with an Argonaute protein, most frequently the AGO1, guiding the control of target expression [7]. MiRNA can regulate their target by cleavage of messenger RNA, translational repression or transcriptional inhibition [8–10]. MiRNA is the best-characterized class of plant sRNA and they are highly conserved among related plant species [11]. Despite conservation of miRNA, a recent study has shown that new miRNA can be gained and old ones can be lost, with a rate of birth and death of the Arabidopsis miRNA genes around one per 1.2-3.3 million years [12]. Accordingly, the miRNA class can be divided into conserved families and species-specific miRNA. In contrast, siRNA is processed from longer double-strand RNA and can be divided into natural antisense transcript siRNA (nat-siRNA), secondary siRNA, like ta-siRNA whose precursor depends on cleavage of miRNA targets, and heterochromatic siRNA (hc-siRNA) produced from intergenic or repetitive regions [5]. At least three RNA-dependent RNA polymerases (RDR1, RDR2 and RDR6) are needed to form siRNA precursors [13–15]. Like miRNA, the precursor of siRNA is processed by DCL and loaded into a RISC complex containing AGO that guides target regulation [5, 7]. However, specific members of the DCL and AGO family of proteins are required in the biogenesis of each different type of siRNA. For instance, RDR2 and DCL3 are preferentially used in the biogenesis of hc-siRNA originating from repetitive regions, and AGO4 is required for its function [16–18]. Repeat-associated siRNA is involved in silencing transposons and other repeat elements by methylation of DNA [19], resulting in epigenetic modifications that mediate gene silencing [20].

Small RNA has been implicated in the interaction between leguminous plants and nitrogen-fixing bacteria [21, 22]. Gramineous plants also establish association with endophytic diazotrophic bacteria, which colonize intercellular spaces and vascular tissues of plants without causing damage to the host plant [23–25]. However, the role of sRNA in the grass-diazotrophic bacteria interaction has not been described. Maize is one of the world’s most widely cultivated crops, valuable not only for human and animal consumption, but also for ethanol production [26]. Previous studies showed that maize interacts with endophytic diazotrophic bacteria, including *Herbaspirillum spp.* and *Azospirillum spp.* [27, 28]. Furthermore, inoculation with diazotrophic bacteria increases maize productivity, demonstrating the benefits of these bacteria for the plant [29]. In addition to its economic importance and the successful interaction with diazotrophic bacteria, maize has several advantages as a grass-model for the analysis of plant-diazotrophic bacteria association by sRNA regulation: its genome is sequenced to a high quality [30]; recent studies have shown that maize sRNA is regulated in response to changes in the environment; and a strong epigenetic regulation occurs in maize due to the high abundance of transposable elements (TE) in the genome [31–33].

In order to understand the roles of sRNA in maize during the interaction with endophytic diazotrophic bacteria, sRNA libraries from maize hybrids (*Zea mays* – UENF 506-8) inoculated with *H. seropedicae* were constructed and sequenced. The analysis uncovered a dynamic regulation of known and novel miRNA in plants inoculated with *H. seropedicae*. Expression analysis in biological replicas and in plants inoculated with another diazotrophic bacteria, *A. brasilense*, showed that the expression of four copper-regulated miRNAs increased in the presence of the bacteria. Targets of that miRNA are involved in copper homeostasis and in defense pathways against pathogenic microorganisms, suggesting that maize colonization by diazotrophic bacteria is facilitated by the attenuation of defense mechanisms. Also, our analysis identified novel miRNA mapping to transposable elements (TE). Additional analysis identified siRNA that matched small regions close to either the 5′ or 3′ ends of coding DNA sequences (CDSs). A reduction in the transcript levels of the corresponding CDS was verified. Finally, an increase in GC and GHC methylation was observed in the same region, suggesting an epigenetic regulation in response to diazotrophic bacteria inoculation. Our findings suggest important roles for sRNA regulation in maize during association with beneficial endophytic diazotrophic bacteria and could assist breeding programs to develop maize or other grasses more efficiently in association with diazotrophic bacteria, which would result in an improvement of crop production.

## Results

### Maize inoculation with diazotrophic bacteria

**Figure 1:**
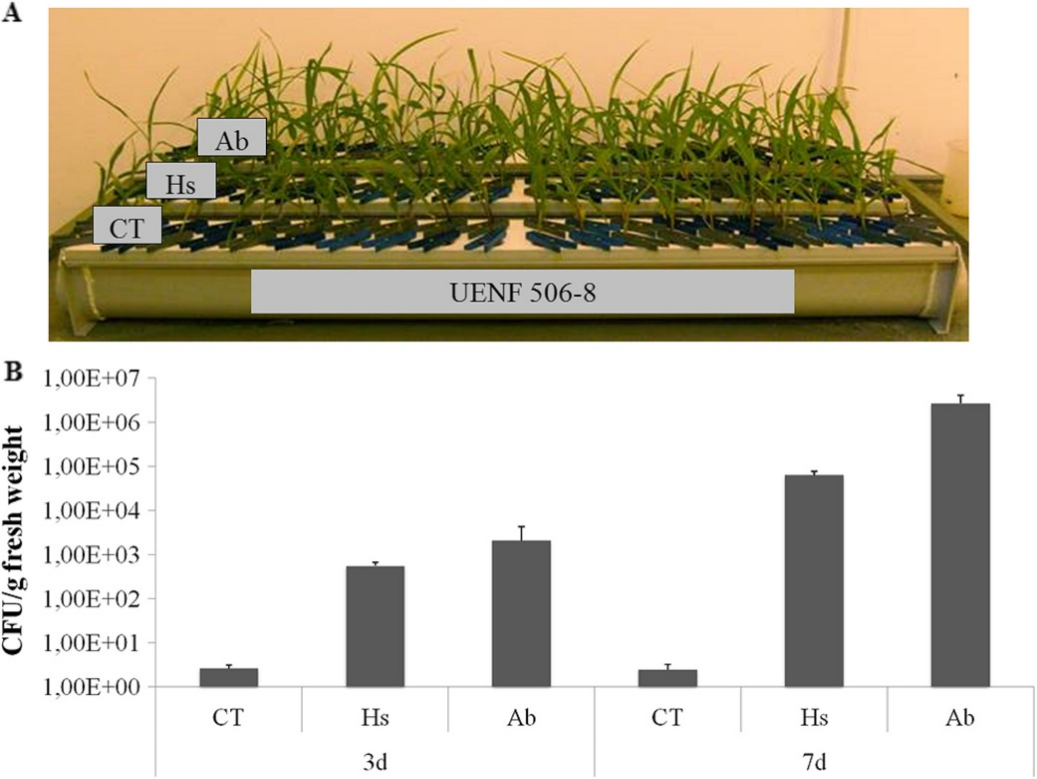
**Maize inoculated with diazotrophic bacteria. (A)** Hydroponic experiment. **(B)** Measurement of colonization levels in maize during the experiment. Two biological replicates were used for each treatment. The error bars represent the standard deviation. CT: mock sample; Hs: inoculation with *H. seropedicae*; Ab: inoculation with *Azospirillum brasilense*; d: days after inoculation.

Diazotrophic bacteria, such as *Azospirillum* spp. and *Herbaspirillum* spp., have been linked with N_2_ fixation in association with agriculturally important grasses, such as maize, sugarcane and rice [23, 27, 34]. To understand the regulation of small non-coding RNA in response to association with diazotrophic bacteria, an experiment was carried out with maize plants grown in hydroponic culture and inoculated with *Azospirillum brasilense* (BR11005) and *Herbaspirillum seropedicae* (HRC54) (Figure 1A). *H. seropedicae* survives poorly in the soil, being considered an obligate endophyte [23]. In maize, these bacteria are isolated from roots, stems and leaves [27]. On the other hand, studies have shown that, depending on the *A. brasilense* strain, these bacteria can survive in the soil, colonizing the root surface, or live within inoculated plants, being considered a facultative endophyte [24, 35]. In the present work, the colonization was confirmed by the most probable number method, which counts colony-formation units (CFU) *per* g of fresh weigh, using plants three and seven days after inoculation (Figure 1B). Both species of bacteria were more abundant in inoculated plants compared with the mock treatment, and there was an increase in bacterial numbers seven days after inoculation. Previous studies showed that seven days after inoculation most species of endophytic bacteria have established themselves inside the plant [36, 37]. Therefore, further analyses were done using samples of plants harvested seven days after inoculation.

### Computational identification of sRNA from library data

**Figure 2:**
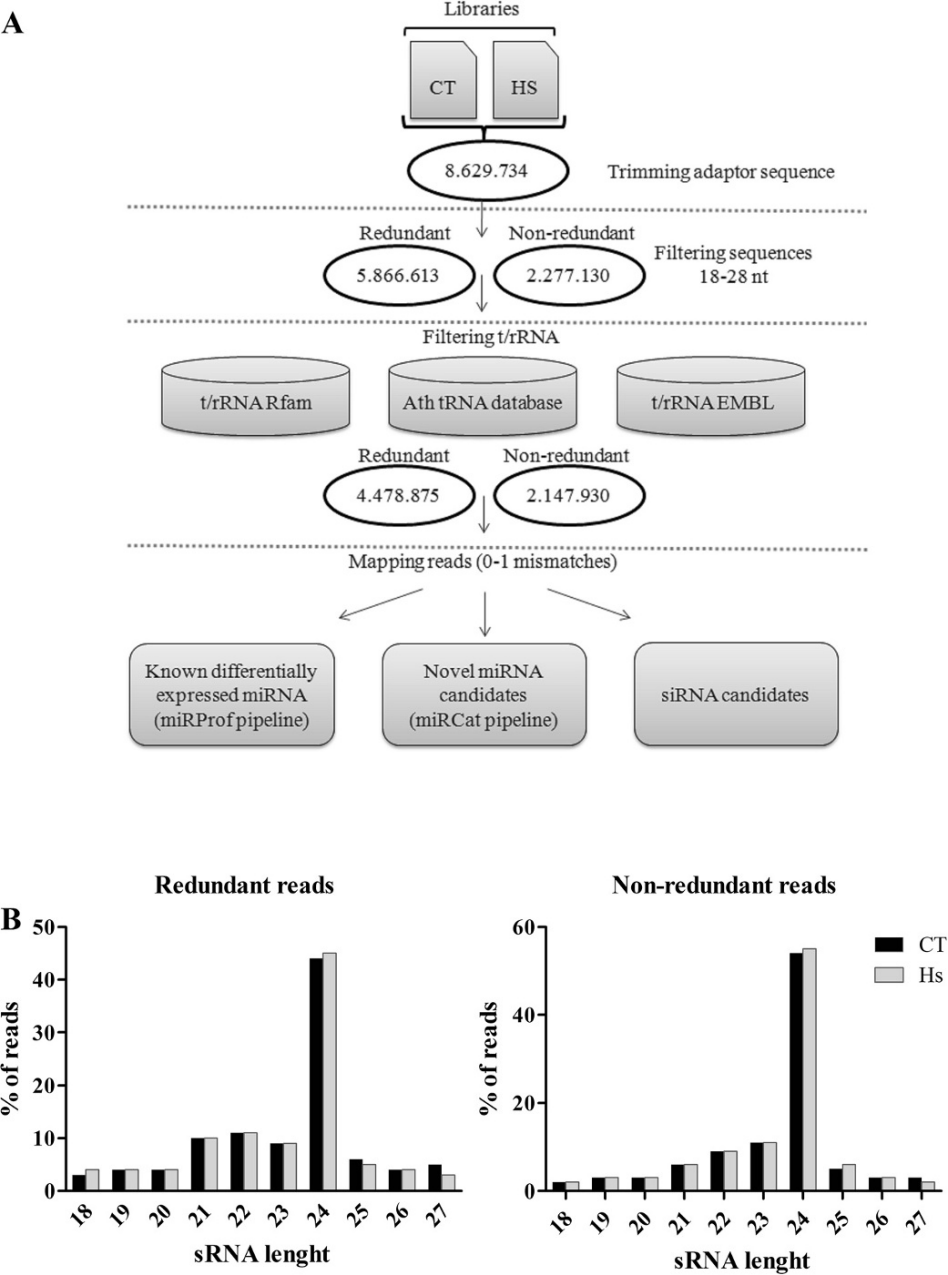
**Analysis of sRNA libraries. (A)** Pipeline used to identify maize sRNAs regulated in response to *H. seropedicae* inoculation. **(B)** Distribution of redundant and non-redundant sRNA dataset. CT: control library; Hs: inoculated library.

Small-RNA libraries prepared from control plants and plants inoculated with *H. seropedicae* were sequenced by Illumina SBS technology, providing altogether 8,629,734 raw reads. The bioinformatics pipeline used in the sRNA analysis is shown in Figure 2A. First, low-quality sequences and adapter sequences were removed; next, sequences ranging from 18 to 26 nucleotides (nt) were selected. After these steps, 4,478,875 redundant filtered reads (corresponding to 2,147,930 non-redundant sequences) from the two libraries were used for sRNA analysis. Their size distribution is shown in Figure 2B. A peak of 24-nt species was observed in the distribution of redundant reads (44% in control library - CT; 45% in inoculated library - Hs) and non-redundant reads (54% and 55% in CT and Hs, respectively), followed by sRNA with 22, 23 and 21 nt in length.

Filtered reads allowed the identification of two classes of sRNA: miRNA and siRNA (Table 1). Using Blastn against the B73 genome (B73 RefGen_v2) and allowing one mismatch, a total of 244 and 308 miRNA sequences were identified in control and inoculated libraries, respectively. From these reads, a search in the miRBase database, release 19, identified 237 and 295 known miRNAs in control and inoculated libraries, respectively. The other miRNAs were classified as putative novel miRNA. Eighteen novel miRNA candidates (7 in CT and 11 in Hs) were identified using the miRCat pipeline, based on the presence of mature miRNA and miRNA* sequences in the same library. The remaining reads - 750,691 in CT and 1,396,687 in Hs- were classified as siRNA candidates (Table 1). Additional analysis of these sequences showed a larger number of non-redundant sequences in the inoculated library.

**Table 1:** **Summary of results obtained from bioinformatics analysis of each small RNA library**

Description	CT	Hs
Filtered reads^a^	750935	1396995
miRNA	244	308
Conserved miRNA^b^	237	295
Novel miRNA^c^	7	11
siRNA candidate	750691	1396687

^a^Filtering for tRNA, rRNA, low-complexity sequence and trimming for “N” bases and 3′ adapters.

^b^Known miRNAs deposited at miRBase database.

^c^miRNAs class I detected by miRCat pipeline.

Overall information of filtered reads, number of miRNAs detected, known miRNAs, novel miRNAs and siRNAs using data from libraries experiment A.

### Changes in abundance of known maize miRNA during association with endophytic diazotrophic bacteria

The known miRNA identified were classified in 25 miRNA families, previously identified in maize or other plants. Next, the abundance of the different miRNA families was inferred from the sum of the abundance of each member and reads were normalized to compare the expression in both libraries. The results showed a dynamic miRNA regulation in response to association with diazotrophic bacteria (Table 2). While 80% of these miRNA families were identified in both libraries, miR172, miR397, miR529 and miR827 were found only in the inoculated library, and miR3630 was present only in the control library. The most abundant family was miR159, followed by miR319, miR168 and miR156 (Table 2). The ratio of normalized reads in the inoculated and the control libraries for each miRNA family showed that most of these miRNA families were up-regulated. The miR398 and miR408 families were the most up-regulated, showing ratios of 13.24 and 9.32, respectively. This result suggests that these miRNAs may have an important role in the maize response to the association with *H. seropedicae*.

**Table 2:** **Differential expression of conserved miRNAs**

miRNA	CT	Hs	Log 2 (Hs/CT)	***p***-value
miR156	945.57	1309.01	0.47	0.000000
miR159	51822.95	59294.59	0.19	0.000000
miR160	119.67	28.49	-2.07	0.000000
miR162	25.12	35.95	0.52	0.246230
miR164	118.20	114.62	-0.04	0.837848
miR166	645.64	1033.64	0.68	0.000000
miR167	234.91	220.43	-0.09	0.524589
miR168	1501.09	1526.72	0.02	0.664597
miR169	76.83	35.27	-1.12	0.000117
miR171	97.51	183.13	0.91	0.000002
miR172	-	13.56	-	0.000977
miR319	1886.70	3144.33	0.74	0.000000
miR3630	29.55	-	-	0.000000
miR390	32.50	25.09	-0.37	0.329586
miR393	59.10	25.09	-1.24	0.000196
miR394	17.73	19.67	0.15	0.867164
miR396	463.92	693.84	0.58	0.000000
miR397	-	21.03	-	0.000015
miR398	19.21	254.34	3.73	0.000000
miR408	91.60	853.91	3.22	0.000000
miR444	53.19	74.61	0.49	0.089931
miR528	132.97	214.32	0.69	0.000037
miR529	-	8.14	-	0.023690
miR827	-	13.56	-	0.000977
miR858	90.12	75.28	-0.26	0.285608

The number of reads found in each library from experiment A was normalized *per* million, and the log_2_ (Hs/CT) was calculated. CT: control library, Hs: inoculated library. The Fisher exact test was performed with Bonferroni correction.

To confirm the miRNA regulation, two more libraries were constructed and sequenced using biological replicates (experiment B) and analyzed as above. These libraries, denominated CTb and Hsb, had 5,137,415 and 2,159,935 non-redundant reads filtered, respectively. The patterns of differential expression of 25 miRNA families identified are available in Additional file 1: Table S1. Comparison between the results from libraries in experiments A and B showed that 15 miRNAs shared the same regulation profile, while 10 miRNAs had contrasting profiles. Interestingly, miR398 and miR408 were up-regulated in both analyses.

### Cu-miRNAs are up-regulated in response to diazotrophic bacteria

**Figure 3:**
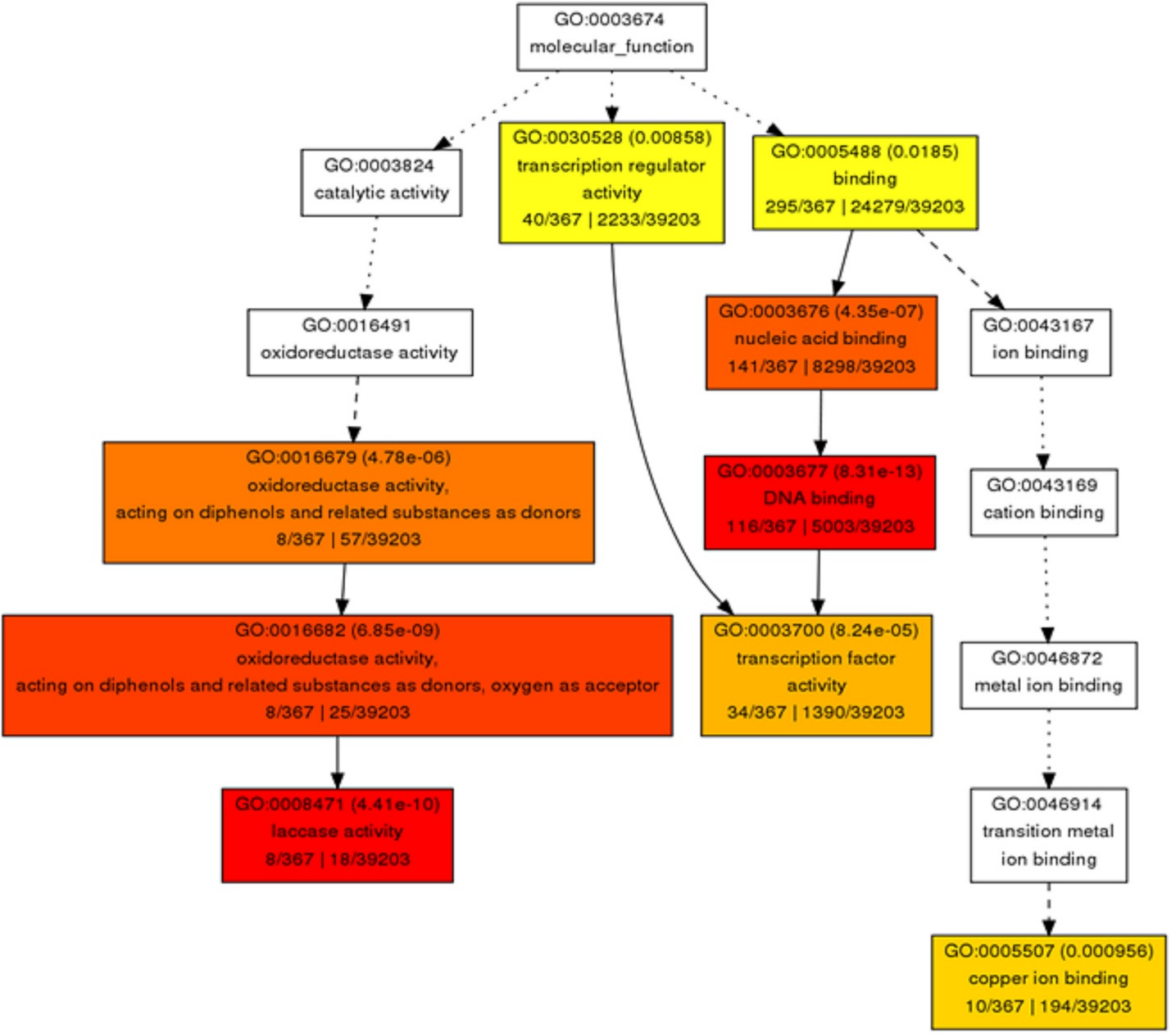
**Molecular function of conserved miRNA targets, based on Gene Ontology (GO) classification.** AgriGO web-based tool was used to analyze GO categorization of genes (http://bioinfo.cau.edu.cn/agriGO/). The graphical output is a GO hierarchical image containing all statistically significant terms. Red color means terms with higher statistical significance. Inside the box: GO term, adjusted p-value, GO description, item number mapping the GO in the query list and background, and total number of query list and background.

In order to understand the biological mechanisms triggered by miRNA in response to the association with diazotrophic bacteria, we identified the targets of regulated miRNA. Using *psRNA* target [38], 536 unique target genes were detected in the maize genome v.2 (Additional file 2: Table S2). An ontology (GO) of the targets was constructed using AgriGO [39]. The results showed a figure with a hierarchical organization with three arms: one for transcription factor activity, one for laccase activity, and one for copper ion binding, respectively (Figure 3). In one of the arms of the figure, we observe transcription factors which are the major targets of conserved miRNA; on the other two arms of the figure, both classes are related to copper metabolism [40].

**Figure 4:**
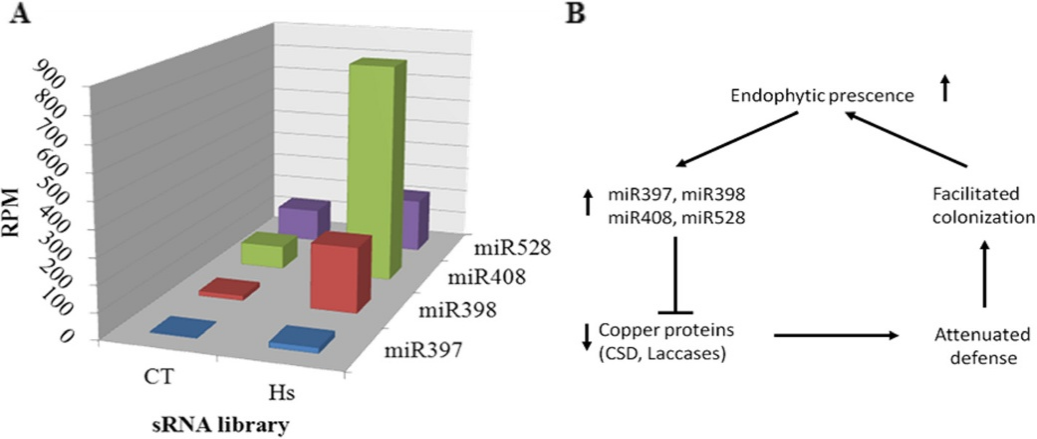
**Regulation of four Cu-miRNAs in response to endophytic association. (A)** Distribution of miRNA profile expression identified by bioinformatics analysis. CT: control library; Hs: *H. seropedicae* inoculated library; RPM: reads per million. **(B)** Putative pathway of regulation of miRNAs/targets involved in copper metabolism.

Accordingly, miR398 and miR408, the two miRNAs with sharply contrasting expression profiles in the inoculated versus the control libraries, are known as copper-regulated small RNA molecules, or Cu-miRNA [41]. Moreover, miR397 and miR528 are also classified as Cu-miRNA because they regulate proteins involved in copper homeostasis. The expression analysis showed that these four maize miRNAs were up-regulated in response to association with *H. seropedicae* (Figure 4A). Given the coordinated expression, we propose a hypothetical model for the regulation of endophytic bacteria colonization in maize: in the presence of endophytic bacteria, Cu-miRNAs are up-regulated, and their targets, Cu-protein, are down-regulated, leading to an attenuation of defense mechanisms and consequently facilitation of colonization (Figure 4B).

**Figure 5:**
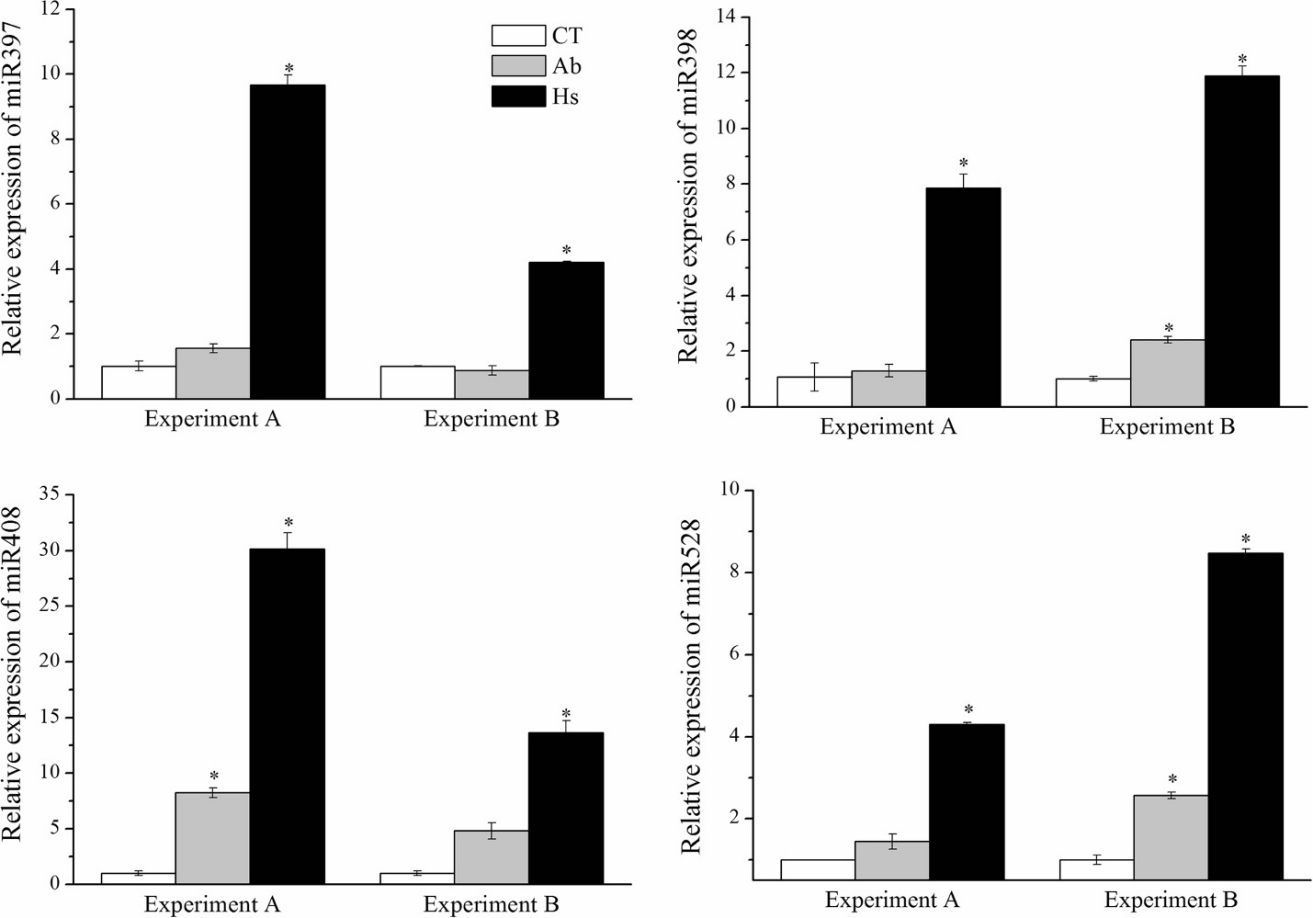
**Expression of Cu-miRNA in plants inoculated with** ***A. brasilense*** **and** ***H. seropedicae*** **.** Relative expression of miR397, miR398, miR408 and miR528 in response to inoculation with *A. brasilense* and *H. seropedicae*. Biological replicates were analyzed (Experiment A and B). The error bars represent the standard deviation between three technical replicates. *represent significant changes in miRNA expression between control and inoculated samples for each experiment (p-value < 0.05).

In order to confirm the model, the expression profiles of the Cu-miRNAs were analyzed by stem-loop qRT-PCR using samples from two independent experiments. Plants were harvested seven days after inoculation with either *H. seropedicae* or *A. brasilense.* The results confirmed an increase in expression of miR397, miR398, miR408 and miR528 in plants inoculated with *H. seropedicae* (Figure 5). Moreover, three of the four miRNAs were also up-regulated in samples inoculated with *A. brasilense.*

**Figure 6:**
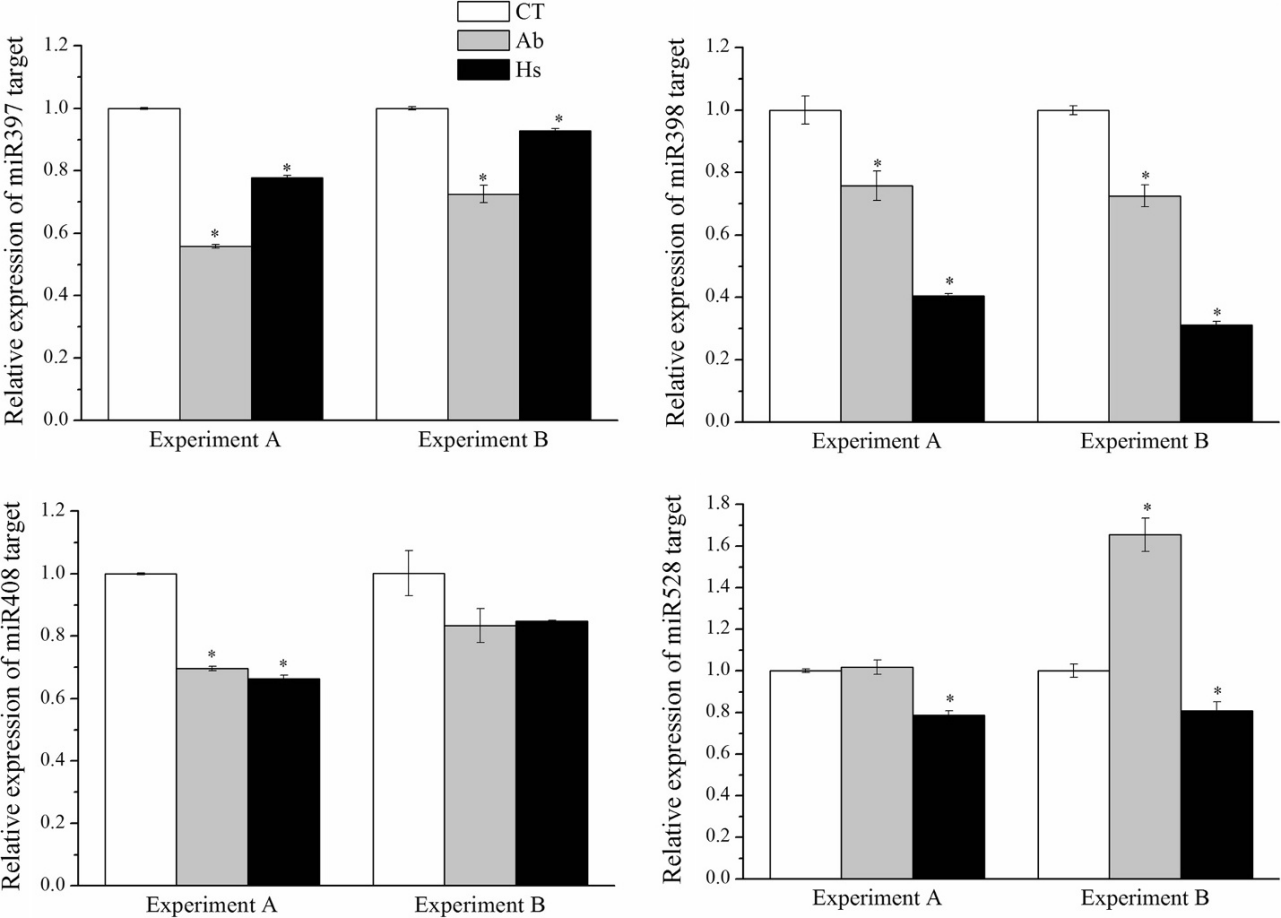
**Expression of Cu-miRNA targets.** Relative expression of Cu-miRNAs targets in response to inoculation with *A. brasilense* and *H. seropedicae* was analyzed using qRT-PCR. Biological replicates were analyzed (Experiment A and B). The error bars represent the standard deviation of three technical replicates. *represents significant changes in target expression between control and inoculated samples for each experiment (p-value < 0.05).

To ascertain whether the putative targets of Cu-miRNA had inverse expression profile, their mRNA levels were measured by qRT-PCR, using the same samples (Figure 6). Basically, the results showed that the targets were down-regulated in samples inoculated with endophytic bacteria. The one exception was the putative target of miR528; in plants inoculated with *A. brasilense* an increase in mRNA accumulation was observed, suggesting that in this situation, this mRNA is either not a target of miR528, or there is an additional layer of transcriptional control of its expression. Altogether, the results support the proposed model for the regulatory pathways triggered in maize in response to endophytic diazotrophic bacteria.

### Identification of novel maize miRNA

Next, a search for novel miRNA candidates was carried out using the miRCat pipeline mapped to the *Zea mays* genome v.2 and a Blast-n search against the miRBase database, release 19. MiRNA sequences that showed no hits to the miRBase database and had a precursor characteristic of miRNA were selected. Based on the criteria for miRNA annotation established by the plant small-RNA research community [42, 43], we selected only miRNA precursors that exhibited miRNA and miRNA* sequences in the same libraries (Class I). Hence, 18 *bona fide* precursors with miRNA/miRNA* complementarity were found, corresponding to 15 novel mature miRNA sequences (Table 3). These miRNAs were denominated Zma_miR_Seq01 to Zma_miR_Seq15. The length of novel miRNA precursors ranged from 75 to 232 nt and their structure with the position of miRNA/miRNA* highlighted is available in Additional file 3: Figure S3. The precursor fold-back structures have an MFE (Minimum Free Energy) ranging from -23.9 to -110.7 kcal/mol, and more than 77% had an MFEI (Minimum Fold Energy Index) value greater than 0.7 (Table 3).

**Table 3:** **New miRNAs identified**

miRNA ID	Length of hairpin precursor	MFE	MFEI ^a^	Randfold p-value	Sequence	sRNA length	RPM - CT	RPM - Hs
Zma_miR_Seq01	169	-42.5	0.61	0.029703	AAAATTTAGAGGACGTTGCTGGAG	24	-	10.34
Zma_miR_Seq02	141	-84.8	1.15	0.009901	AAACCGTCGGAGATAGCTTATGTC	24	-	8.86
Zma_miR_Seq03	128	-76.7	1.08	0.009901	AACCTATGTCCGACGGTTTTAGGC	24	-	7.39
Zma_miR_Seq04	79	-26.4	0.52	0.09901	ACGTCTATGGTTAGATCACGCGGC	24	-	8.86
Zma_miR_Seq05	75	-23.9	0.70	0.049505	ATAACTGTAGTGCATTAAAGCGGG	24	4.07	7.39
Zma_miR_Seq06	173	-60.52	0.85	0.009901	ATCCATATGGACTGGGAGGAAAGC	24	-	44.32
Zma_miR_Seq07a	104	-58.3	0.80	0.009901	CCCGCCGGCGAGCGCTTTCCT	21	-	13.30
Zma_miR_Seq07b	104	-58.3	0.80	0.019802	CCCGCCGGCGAGCGCTTTCCT	21	-	13.30
Zma_miR_Seq08	170	-91.5	0.92	0.009901	CGGCGGGGGCGAACTGAGAAC	21	21.70	143.31
Zma_miR_Seq09a	161	-64.2	0.84	0.009901	CGTGGTATTGTTTCGGCTCATG	22	11.53	125.58
Zma_miR_Seq09b	123	-57.4	0.94	0.009901	CGTGGTATTGTTTCGGCTCATG	22	11.53	125.58
Zma_miR_Seq09c	123	-57.4	0.94	0.009901	CGTGGTATTGTTTCGGCTCATG	22	11.53	125.58
Zma_miR_Seq10	114	-31.3	0.68	0.059406	TCCTTGTTGGACAGATAAAGGAGC	24	-	7.39
Zma_miR_Seq11	85	-45.5	1.17	0.009901	TTGTTGGTCTATTCGGGTTTTCGA	24	-	7.39
Zma_miR_Seq12	143	-57.8	0.81	0.009901	CATGAACCGAGCGAGCTAGCGAGC	24	14.92	-
Zma_miR_Seq13	232	-110.7	0.75	0.009901	GGCGGACTGGGAACACATGGG	21	7.46	-
Zma_miR_Seq14	152	-103.3	1.45	0.009901	TTGGGAGCCACAAAACTGAAG	21	3.39	-
Zma_miR_Seq15	179	-84	1.00	0.009901	TTTTGTTGGTGGTCATTTAACC	22	14.24	-

^a^minimal folding free energy index (MFEI) was calculated according to Zhang et al. [99].

CT: control library, Hs: Inoculated library, RPM: reads per million, MFE: Minimum Free Energy.

In addition to the precursor analysis, the length and the abundance of new mature miRNA was also examined. The majority of the novel miRNAs are 24 nt in length (9 out 15 sequences), four were of 21 nt and two were of 22 nt. To compare the expression of novel miRNA, we used the same approach described for expression analysis of known miRNA. Eleven new miRNAs were identified in the inoculated sample, three of which were also found in the control library but at lower levels. Four novel miRNAs were exclusive to the control library (Table 3). Next, their putative targets were predicted using *psRNA* target. A total of 131 putative targets for the 15 mature new miRNAs were identified (Additional file 1: Table S1). However, more than 87% of the putative targets are uncharacterized genes. Therefore, the molecular function of these novel miRNAs is unknown.

In order to investigate the role of these novel miRNAs, further analyses were performed using the POPcorn website (http://popcorn.maizegdb.org/main/index.php) to map them in the maize genome v.2. The results showed the precursors of new miRNAs scattered throughout the genome (Additional file 4: Figure S4). Next, also using the POPcorn website, the precursors were compared with TEs from the Maize Transposable Elements database. In this analysis, seven of the new miRNAs aligned with different classes of maize DNA transposon, among them *PIF/Harbinger*, *CACTA*, *Mutator* and *hAT* (Table 4).

**Table 4:** **Novel miRNAs mapped at Transposable Elements**

miRNA ID	TE class
Zma_miR_Seq01	PIF/Harbinger
Zma_miR_Seq02	CACTA
Zma_miR_Seq03	CACTA
Zma_miR_Seq05	Mutator
Zma_miR_Seq06	PIF/Harbinger
Zma_miR_Seq11	hAT
Zma_miR_Seq12	hAT

Novel miRNA precursors were mapped against TEs from the Maize Transposable Elements Database (http://maizetedb.org/~maize/).

### siRNA production derived from repeats and CDSs

**Figure 7:**
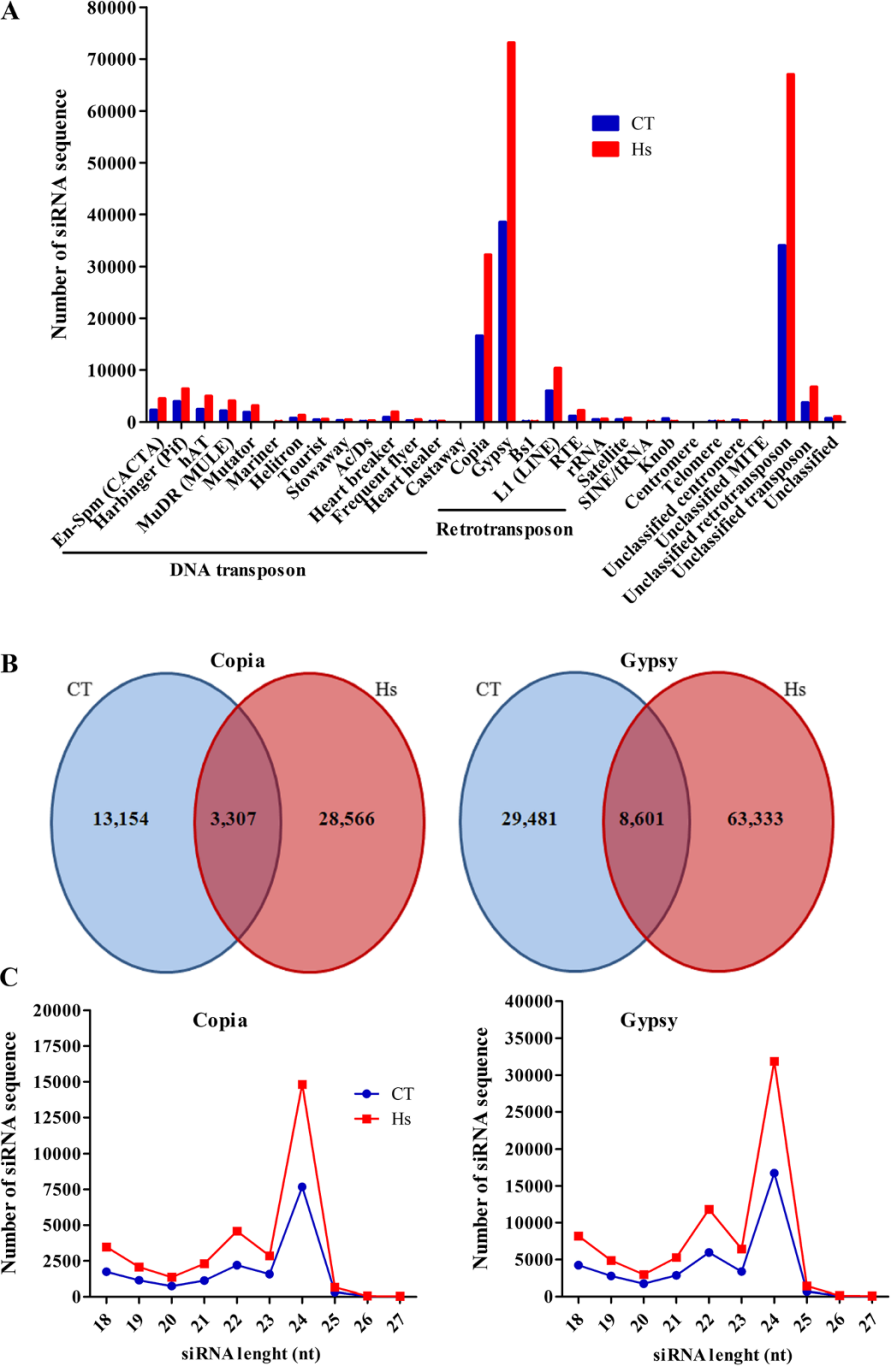
**siRNA mapped to repeats. (A)** Distribution of siRNA that aligned with the Repbase database. **(B)** Number of siRNA identified in each library matching *Copia* and *Gypsy*. **(C)** Length distribution of siRNA related with *Copia* and *Gypsy*. CT: control library; Hs: *H. seropedicae* inoculated library.

In order to find repeat-associated siRNA in maize sRNA libraries, we aligned the siRNA candidates (Table 1) in the maize repeat database (Repbase, v.18) using the Bowtie alignment and allowing three mismatches. Only the best hits of aligned siRNA from each library were selected for further analysis, giving a total of 117,935 and 222,432 siRNAs from control and inoculated libraries, respectively. These siRNAs aligned with different classes of TE, among them 14 classes of DNA transposon, five classes of retrotransposon, rRNA, satellite, SINE/tRNA, Knob, centromere, telomere and a number of unclassified repeats (Figure 7A). Approximately eight percent of the siRNAs in each library came from a DNA transposon, and approximately 32% were from a retrotransposon, especially *Copia* and *Gypsy*. In all classes, more siRNA were identified in the inoculated than in the control library. Nevertheless, a small fraction of the total siRNA was present in both libraries. For instance, 3,307 siRNAs from *Copia* were identified in both libraries, while 13,154 and 28,566 were exclusive to the control and inoculated library, respectively. Figure 7B shows the number of exclusive and shared siRNA from *Copia* and *Gypsy*. These siRNA were analyzed for size distribution, and the majority was 24 nt in length, with a smaller population of 22 nt (Figure 7C).

**Figure 8:**
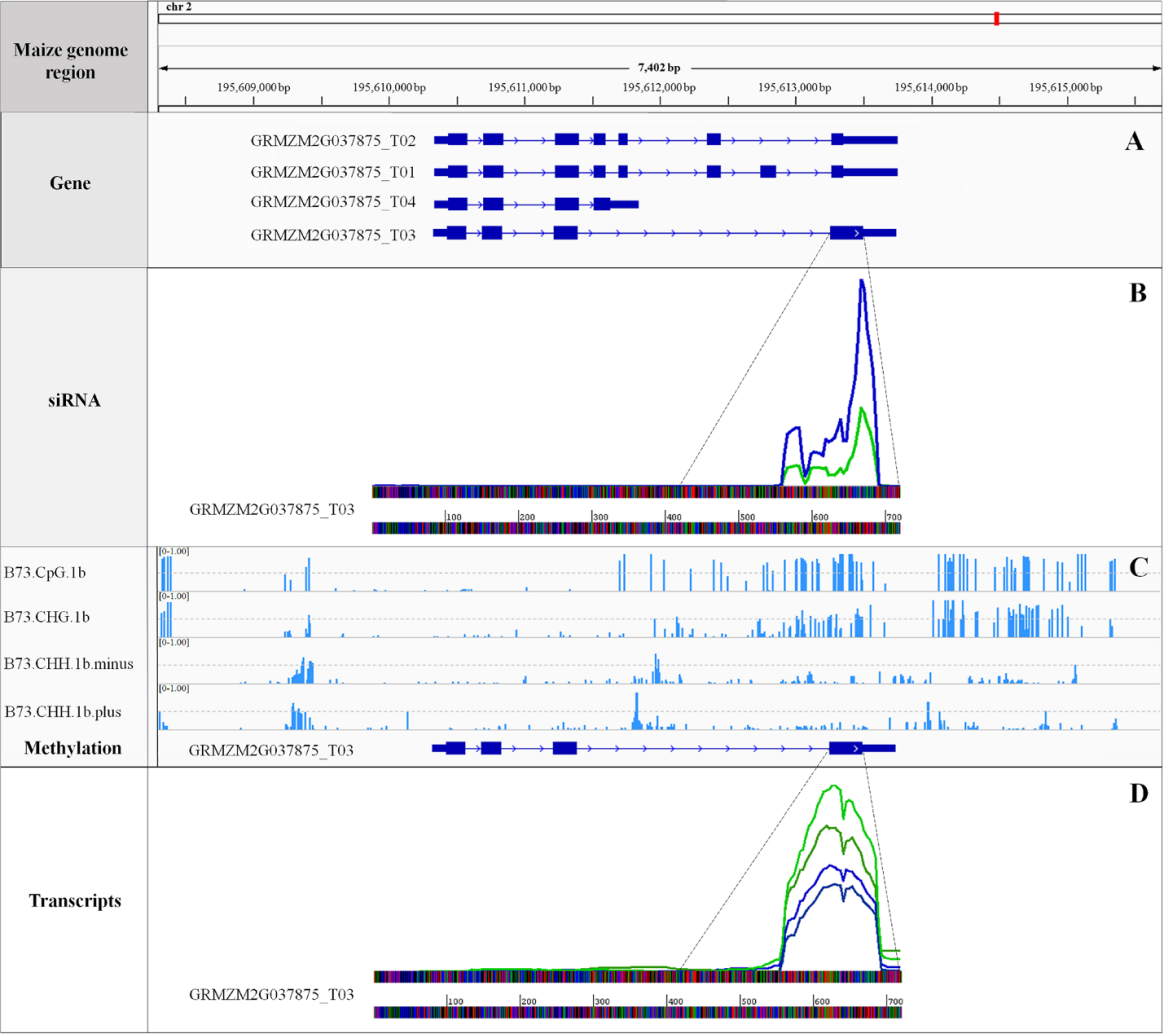
**Analysis of siRNA, methylation profile and mRNAseq reads mapped to the sequence of a splice variant of GRMZM2G037875. (A)** Localization of GRMZM2G037875 gene in chromosome 2 and four possible splice variants from this gene (boxes represent exons, and lines are introns). **(B)** Position of siRNA aligned in GRMZM2G037875_T03. The two peaks, blue and green, are non-redundant siRNA sequences from inoculated and control libraries, respectively. **(C)** Four data tracks show methylation levels for B73 in different sequence contexts. Methylation levels are displayed on a scale from 0 to 1. **(D)** Position and abundance of transcripts that matched in GRMZM2G037875_T03. Blue and green indicate transcript sequences from inoculated and control libraries, respectively.

Additional analyses were performed to classify siRNA with homology to CDSs. A total of 237 and 456 CDSs aligned with more than 50 unique siRNAs from control and inoculated libraries, respectively. Among these, five CDSs had more than 1,000 siRNA sequences from the inoculated library aligned with them. Two CDSs that showed the highest amounts of siRNA aligned, GRMZM2G037875_T03 and GRMZM2G487629_T02, were selected to carry out further analyses. GRMZM2G037875_T03 aligned with 721 and 1545 unique siRNA sequences in control and inoculated libraries, respectively. This difference was statistically significant. In each library, 71% of the siRNAs were 24-nt species. Almost all siRNAs mapped onto the fourth exon of GRMZM2G037875_T03 (Figure 8). Interestingly, this splice variant of the GRMZM2G037875 gene had an exclusive region at the 3′-end on the fourth exon where the siRNA mapped. Because 24 nt can trigger DNA methylation, the methylation profiles of these CDSs were investigated [44]. The results show that the region where the siRNA mapped was highly methylated in CG and CHG sites (Figure 8). In order to verify the expression of the CDSs, a dataset of mRNAseq obtained from the same biological samples (Carvalho *et al.,* personal communication) was examined. A total of 249.6 and 170.8 normalized reads from the control transcriptome library and 192.9 and 135.6 reads from the inoculated library mapped onto GRMZM2G037875_T03, with significantly more reads mapped at the 3′-end on the fourth exon in the control library than in the inoculated library, showing an inverse regulation to that of the siRNA. A similar analysis was performed for CDS GRMZM2G487629_T02 (Additional file 5: Figure S5), and almost all of the siRNA mapped onto the 5′-end of these CDSs. Normalized siRNA that aligned with these CDS were 1026.72 and 1046.53 from control and inoculated libraries, respectively. Despite a small increase in siRNA in the inoculated library, this difference was not significant; but the methylation analysis also revealed an enrichment of methylated sites in the same region of the siRNA peak (data not shown).

## Discussion

Recently, plant sRNA regulation has been shown to play important roles in plant development, nutrition homeostasis, response of abiotic stress and the plant-microbe interaction, including interaction with pathogens, and in rhizobia-legume symbiosis [45–48]. One characteristic of the association of leguminous plants and rhizobia bacteria is the development of structures called root nodules, where bacteria establish and contribute to the plant with biologically fixed nitrogen [22]. Endophytic nitrogen-fixing bacteria have also been isolated from gramineous plants. However, in grasses, the diazotrophic bacteria are found colonizing intercellular spaces and vascular tissues of most plant organs, without forming any particular structure [24, 49]. Although several studies have described the benefits of grass-diazotrophic bacteria interactions and the molecular pathways involved in this association [50–52], little is known about the role played by sRNA in response to colonization by endophytic diazotrophic bacteria in grasses, like maize. One study has shown that the maize hybrid line UENF 506-8 has an efficient association with diazotrophic bacteria [53]; this hybrid, therefore, was used in the present study.

The main categories of plant regulatory sRNA are miRNA and siRNA [47]. Our analysis identified a set of maize miRNA and siRNA regulated by the association of the plant with diazotrophic bacteria. Maize sRNA libraries made from seedlings inoculated for seven days with the endophytic diazotrophic bacteria *H. seropedicae* (HRC54) and control seedlings were constructed and sequenced. The distribution profile of sequenced sRNA showed that the most abundant and most complex fraction was the sRNA of 24 nt in length, followed by 22, 23 and 21 nt, in agreement with published reports of maize small- RNA libraries [54–56].

Based on characteristics of plant miRNA conservation, 25 miRNA families were identified in the analysis. Among the miRNA most expressed in plants inoculated with *H. seropedicae,* miR159 and miR168 were also identified as being regulated in soybean nodules [22], suggesting a role for these miRNAs in the association of diazotrophic bacteria with plants. Interestingly, these miRNAs were previously characterized as miRNA modulated in response to pathogen infection. MiR159 was up-regulated in Arabidopsis inoculated with *Pseudomonas syringae* [57], as well as in maize inoculated with beneficial bacteria. In contrast, the miR393 expression profile in response to diazotrophic bacterial inoculation was different from that observed in a pathogenic infection. MiR393 was the first miRNA described to be involved in the regulation of plant immunity [58]. This miRNA was induced in plants infected with *Pseudomonas syringae*, contributing to antibacterial resistance [57, 59]. On the other hand, in maize inoculated with diazotrophic bacteria, a repression of miR393 was observed, suggesting that in the presence of *H. seropedicae,* the defense response through the miR393-based regulation pathway was not activated.

More recently, miR397 has been shown to be involved in nitrogen fixation-related copper homeostasis in *Lotus japonicus* [21]. MiR397 was classified as Cu-miRNA, because its target is an mRNA that encodes laccase protein involved in copper homeostasis. In maize, four Cu-miRNAs - miR397, miR398, miR408 and miR528 - were up-regulated and their targets were down-regulated in response to *H. seropedicae* inoculation. There are canonical targets described for these miRNAs and the cleavage of these targets by their respective miRNAs was previously confirmed using RACE 5′ PCR [60–62]. Copper is an important micronutrient and serves as a cofactor for proteins involved in important pathways, among them photosynthesis and the metabolism of scavenging reactive oxygen species [63]. In plants, targets of other evolutionarily conserved miRNA encode genes involved in copper homeostasis, such as laccase, copper superoxide dismutase (CSD) and cupredoxin [41]. The described targets of miR397, miR408 and miR528 in maize are laccases and cupredoxins [64], important copper protein families with redox activities [65] whose domains are conserved in other enzymes [66]. More so, in maize, cupredoxins are involved in oxidative stress response signaling, mediating electron transfer or oxidation homeostasis during stress [67]. Biotic stress causes accumulation of reactive oxygen species as an early response to pathogen attack [68]. The oxidative burst can result in the direct death of pathogens, acting as a mechanism of plant defense [69]. Previous work has shown that miR408, after 14 h, is repressed in Arabidopsis plants inoculated with the pathogenic bacteria, *Pseudomonas syringae* pv. *Tomato*, while its target was induced [70], suggesting that biotic stress can trigger down-regulation of Cu-miRNA, increasing the steady-state of their targets, generating a burst of oxidative stress. In plants inoculated with endophytic diazotrophic bacteria, an inverse miRNA/target regulation was observed: miR397, miR408 and miR528 were induced, and their targets were repressed. These results suggest that *H. seropedicae* does not activate the early defense response against bacterial colonization in maize. A similar regulation of these miRNAs occurs in plants inoculated with *A. brasilense.* Furthermore, an additional role for miR408 in plant/beneficial interaction might be to reduce lignin biosynthesis as a consequence of decreased laccase activity [71], facilitating colonization by endophytic bacteria.

Copper superoxide dismutase is responsible for removing reactive O_2_ species (ROS), reducing oxidative stress [72]. Superoxide dismutase is the first line of defense to convert this reactive oxygen species [73]; accordingly, during pathogenesis, CSD accumulates due to the repression of miR398 expression [72, 74]. In contrast, the up-regulation of miR398, and consequently, the repression of its target, suggests once more that beneficial endophytes such as *H. seropedicae* and *A. brasilense* are not recognized as pathogens by maize and therefore do not trigger defense responses such as ROS production.

A subset of miRNA is considered non-conserved if it is present only in certain plants or in closely related species [75, 76]. The miR528, for instance, is an example of monocot-specific miRNA [77]. Non-conserved miRNA is the result of emerging classes of lineage-specific families, and it originated from recently evolved MIR genes [78]. Here, we have identified 18 *bona fide* precursors of novel miRNAs, which obey all the rules established for identification of new microRNA, including the identification of miRNA and miRNA* sequences in the same library. The majority of these new miRNAs are 24-nt species, longer than canonical 21-nt miRNA [79]. A recent study proposed that miRNA with 24 nt is derived from precursor cleavage by DCL3 and that its biogenesis is similar to that of siRNA [80, 81]. Because these novel miRNAs had not been identified in other plants, they probably have emerged recently as lineage-specific miRNA. The evolution of miRNA genes has been widely discussed and two hypotheses for miRNA birth have been postulated: miRNA genes could originate from inverted gene duplication [82] or randomly from repetitive elements present in the genome [83, 84]. In support of the last model, a recent study revealed the co-localization of maize miRNA within TE sequences [64]. Furthermore, analysis of 163 miRNAs evolving from repetitive elements in four plants, Arabidopsis, poplar, rice and sorghum, demonstrated that a considerable number of young miRNA identified were species-specific [83]. In our study, a subset of the novel miRNA identified was characterized as repetitive element-related miRNA, suggesting that they are young miRNA. Although the targets of the novel miRNAs have not been determined, most of these miRNAs are induced in response to plant-diazotrophic bacteria association and their targets could play a role in the plant’s interaction with diazotrophic bacteria.

More frequently in plants, the number of miRNA families related to TE is smaller than the number of miRNA families not related to TE [85]. However, TEs are highly abundant in maize, comprising more than 80% of the genome [30]. Repeat-associated siRNA has been identified in maize, especially siRNA matching retrotransposons. This corroborated data that showed that the maize genome has more retrotransposons than DNA transposons, with *Gypsy* being the most frequent class [86]. The majority of siRNAs derived from TE have 24 nt followed by 22-nt species. Repeat-associated siRNA is most commonly of 24 nt, and recent studies have suggested that RDR2 and DCL3 are required for the biogenesis of this siRNA class, while 24 nt guided-DNA methylation is dependent on DCL4 [5, 17, 18]. Additionally, there is evidence of other mechanisms involved in the production of siRNA from repeats, inferred from an enrichment of 22-nt siRNA that was seen in the MOP1 mutant, an ortholog of RDR2 [87]. In plants, despite the existence of many TEs, they are usually inactive. SiRNA could be a tool for controlling their own TE precursors, acting as a feedback mechanism [88]. Interestingly, TEs are activated in response to stress, including pathogen infection, mechanical stress or abiotic stress [89, 90]. Accordingly, TEs are demethylated during pathogen infection in Arabidopsis, relevant because TE demethylation is thought to take part in the plant defense genes’ activation [91]. On the other hand, several siRNAs from TEs were identified only in the inoculated library, suggesting that siRNA may also have an important role in TE silencing, resulting in a more efficient plant association with beneficial bacteria. Based on this, it is possible to propose that plants can sense pathogenic and beneficial microorganisms differently and trigger specific epigenetic-mediated regulatory mechanisms.

It has been shown that siRNA that match protein-coding genes can regulate gene expression [17, 92]. In this study, genes with a large number of siRNA aligned with their CDSs were identified, five among them with more than 1,000 unique siRNA reads. Although, these genes are classified as unknown, the abundance and complexity of the siRNAs mapping onto these genes suggest that its silencing could be important for diazotrophic bacteria association, given that more siRNAs were identified in the inoculated sample. GRMZM2G037875_T03 is the CDS with greatest number of unique siRNAs aligned with it. Interestingly, a hotspot of mapped siRNAs is located at the 3′-end of the CDS, a region that is exclusive to one splice variant of GRMZM2G037875. In contrast, for another CDS, GRMZM2G487629_T02, siRNAs were aligned at the 5′-end. According to a recent study in Arabidopsis, siRNA related to protein-coding genes can be generated by the RDR2-DCL3 pathway, but the mechanism that regulates gene expression of protein-coding genes by siRNA is not well understood [17]. One hypothesis is that the siRNA is loaded onto AGO4-containing complexes to guide methylation of target genes. Accordingly, the gene regions enriched for siRNA are also enriched in sites of CG and CHG methylation [44]. For two CDSs (GRMZM2G037875_T03 and GRMZM2G487629_T02), the results suggested that siRNA can mediate the DNA methylation. Also, the majority of maize siRNA that aligned in this CDS is 24 nt in length, corroborating the hypothesis that 24 nt siRNA triggers DNA methylation [93]. DNA methylation at the 5′ or 3′-end has been correlated with the silencing of genes, consequently leading to the reduction of gene transcription [94, 95]. Interestingly, the levels of GRMZM2G037875_T03 transcript were reduced in transcriptome analysis of plants inoculated with *H. seropedicae*, suggesting that this splice variant was methylated by siRNA, leading to a decreased transcription. This information can help to understand the regulation of this siRNA class; however, further studies should be performed to uncover the function of the genes, in particular the role of the splice variant that is enriched in the plants inoculated with diazotrophic bacteria.

## Conclusions

Relatively little is known about plant epigenome mechanisms involved in the plant response to diazotrophic bacteria. Our results show that plants may use a variety of sRNA regulation mechanisms to regulate and favor this association, and that the mechanisms activated are in contrast with the ones previously described for pathogen infection. In conclusion, our data suggest that maize, and possibly other grass species, have RNA-based silencing mechanisms that can trigger specific responses when plants interact with microorganisms to establish either a beneficial association or to fight pathogenic infection.

## Methods

### Plant material and diazotrophic bacteria inoculation

Maize seeds of the hybrid UENF 506-8 were surface-sterilized for 15 min with a 10% (v/v) solution of commercial bleach containing 5.25% (w/v) NaCl, then washed several times with distilled water. After soaking overnight in distilled water, seeds were germinated at 25°C in wet paper. Seven days after germination, seedlings were transferred to a 0.5x Hoagland’s solution [96] in a growth chamber at 24°C with a 10 h photoperiod and left for two weeks. The Hoagland’s solution was renewed every 3 or 4 days. After this period, seedlings were inoculated with two diazotrophic bacteria, *A. brasilense* (BR11005) and *H. seropedicae* (HRC54), as described by James et al. [97]. Suspensions (150 uL) containing 10^-6^ to 10^-7^ diazotrophic bacteria were added to each 30 mL of the plant growth medium. Control plants were mock inoculated. Seven days after inoculation, whole plants were harvested, bacteria colonization was evaluated by the Most Probable Number (MNP) estimation [98], and quickly frozen in liquid nitrogen. Four experiments were performed, of which two were used for Illumina sequencing, while the other two were used to validate the sequencing analysis. Total RNA was isolated from whole plants using Trizol (Invitrogen, CA, USA) as described by the manufacturer. RNA purity was analyzed using a Thermo Scientific NanoDrop™ 2000c spectrophotometer and the RNA integrity was verified by electrophoresis on a 1% agarose gel.

### Construction and analysis of small RNA libraries

Two sRNA libraries made from control maize hybrid (UENF 506-8) seedlings and seedlings inoculated for seven days with diazotrophic bacteria, *H. seropedicae* (HRC54) were constructed and sequenced; two biological replicas of each library were sequenced. Total RNA (~10 μg) from control and *H. seropedicae* inoculated plants was sent to Fasteris Life Sciences SA (Plan-les-Ouates, Switzerland) for small RNA library construction and subsequent sequencing by Illumina technology. Quality of the sequences was evaluated by measurement of the quality of the reads according to the percentage of bases having a base quality greater than or equal to 30 (Q30). On average, 80% of each channel had Q30 quality. Next, 3′ Illumina adapters (CTGTAGGCACCATCAAT) and “N” bases trimmed of the reads and sequences within the 18-28-nt range were separated for further analysis.

### Bioinformatics analysis

After trimming and filtering, the remained reads were subjected to the University of East Anglia (UEA) sRNA toolkit (Plant version) miRProf, which allows the selection of conserved mature miRNA and provides the miRNA expression profile. The sRNA libraries data have been submitted to NCBI - Gene Expression Omnibus (http://www.ncbi.nlm.nih.gov/geo/) under accession number GSE47886. The miRProf was run with sRNA of minimum size 18 nt, maximum size 28 nt. This tool matches sRNA libraries with known Viridiplantae mature miRNA deposited in miRBase database release 19 (http://www.mirbase.org/ftp.shtml), using a PatMaN program. The output of the miRProf showed sequences of miRNA that had at most one mismatch with the miRBase database and contained information about total and non-redundant sequence counts. To allow comparison between libraries, counts were normalized. Normalized counts are given in reads per 1 million (RPM) and the total reads after the final trimming and filtering steps were used for normalization. The fold changes were calculated by l log_2_ (Hs/CT). The statistical analysis (Fisher exact test) was performed with a *p-value* cutoff < 0.05 and Bonferroni correction.

Novel miRNAs were identified in the maize libraries using the UEA sRNA toolkit (Plant version) miRCat pipeline. Sequences were mapped to the maize genome (B73 RefGen_v2) to find clusters of sRNA. The most abundant sRNA read within a cluster was chosen as the likely miRNA candidate. Only new miRNA candidates with a corresponding miRNA* were further analyzed. The flanking sequences surrounding the sRNA were extracted from the genome using a 75-nt window length. Each sequence window was then folded using RNAfold. The precursors of the miRNA candidates were tested using randfold (using a cutoff of 0.1), and an additional minimal folding free energy index (MFEI) was calculated according to Zhang et al. [99]. In order to be classified as novel miRNA, candidate sequences were searched against miRBase database release 19 using standalone BLAST [100], with default parameters. The folding structures of the new miRNA precursors identified were obtained with the UEA sRNA toolkit-RNA hairpin folding and annotation tool, which uses the Vienna Package to obtain the secondary structure of a precursor sequence, highlighting the miRNA/miRNA* sequences on the hairpin structure. Next, these precursors were mapped in the maize genome using BLASTn at MaizeGDB (http://popcorn.maizegdb.org/main/index.php) and a CViT image of the B73 assembly was created using information from the Maize Genome Sequencing Consortium [101]. Additional analyses were performed on the MaizeGDB website to compare the precursor of novel miRNA against TEs from the Maize Transposable Elements Database (http://maizetedb.org/~maize/).

### Prediction of miRNA targets

To identify the putative miRNA targets, we used the Plant Small RNA Target Analysis Server, *psRNA Target* (http://plantgrn.noble.org/psRNATarget/). In this investigation, we used the maize genome sequence - B73 RefGen_v2, and the following parameters: maximum expectation less than 3.0; 20 bp of length for complementarity scoring; target accessibility equal to 25; flanking length around target site for target accessibility analysis was 17 bp in upstream and 13 bp in downstream; and range of central mismatch leading to translational inhibition was 9-11 nt.

In order to obtain a functional characterization of the putative targets of conserved miRNA, the maize genome locus for each target was submitted to agriGO [39]. The singular enrichment analysis (SEA) was performed to find enriched GO terms within annotated miRNA targets.

### Characterization of siRNA candidates

After the identification of miRNAs (conserved and novel), the remaining sRNAs were classified as siRNA candidates. The classification of these siRNAs was performed using two approaches: the identification of siRNA related to repeats, and siRNA related to CDSs (Coding DNA Sequences). Both analyses used the program Bowtie, release 0.12.9 [102], to align the siRNA candidates against specific databases. Only the best reads were selected and three mismatches were allowed. In the first alignment, we used sequences of maize repeats from the Repbase, v.18 [103]; and in the second, we used maize CDS dataset of the B73 RefGen_v2. From CDSs aligned, we selected two genes that had the largest numbers of matches to non-redundant siRNA. The alignment files were converted to bam using SAMtools [104] and siRNA matches in maize CDS were quantified in Artemis.

Additional analyses were performed in Cold Spring Harbor Laboratory using their maize methylation database to identify the methylation sites in two CDS regions (GRMZM2G037875_T03 and GRMZM2G487629_T02). The identification of maize transcripts was done in the maize transcriptome database available in our laboratory. The mRNAseq was performed using the same samples used for the construction of the sRNA libraries.

### Validation of bioinformatics analysis by qRT-PCR

The expression profiles of four Cu-miRNAs (miR397, miR398, miR408 and miR528) were assayed by stem–loop qRT-PCR [105, 106]. Total RNA extracted from two independent experiments of plants inoculated with *A. brasilense*, *H. seropedicae* and control plants was treated with DNaseI (Promega). Total RNA was then reverse transcribed into cDNA using Super-ScriptIII reverse transcriptase (Invitrogen). In the same reaction, RT primers specific for each miRNA sequence and random primers were used to enable the amplification of constitutive genes and of the miRNA targets. With this cDNA, qRT-PCR was used with SYBR Green PCR Master Mix (Applied Biosystems). To each well, 1 μL of first strand cDNA, 5 μL of SYBR Green solution, 2 μL of the forward primer (10 μM) and 2 μL of reverse primer (10 μM), designed as described in the protocol [106], were added. Two housekeeping genes were used as internal controls: Ubiquitin (F/5′ AGACCCTGACTGGAAAAACC 3′; R/5′ CGACCCATGACTTACTGACC 3′) and Actin (F/5′ CAATGGCACTGGAATGGT 3′; R/5′ ATCTTCAGGCGAAACACG 3′). qRT-PCR was performed using Applied Biosystems 7500 Real-Time PCR Systems. In the expression analysis of miRNA targets, the following primers were used: miR397 target (F/5′ GTTCGATGTGCAAATGACCAA 3′; R/5′ CCGTCACGATGCTCTTGCT 3′), miR398 target (F/5′ TCTCATTATTCTCATGTGTTCTCAGTTC 3′; R/5′ CGGCGACGGCAACAAG 3′), miR408 target (F/5′ CCAAGAGACGCCAGTGAAGAG 3′; R/5′ TACTGCCCGTTCACCGTGAT 3′) and miR528 target (F/5′ CCCAGCACTCATTCCATAGCA 3′; R/5′ CCCAGCACTCATTCCATAGCA 3′).

## Availability of supporting data

The data set supporting the results of this article is available in the NCBI - Gene Expression Omnibus repository, (http://www.ncbi.nlm.nih.gov/geo/) under accession number GSE47886.

## Electronic supplementary material

Additional file 1: Table S1: Differential expression of conserved miRNAs in replicates of libraries. The number of reads found in each library was normalized *per* million, and the log_2_ (Hsb/CTb) was calculated. CTb: control library of experiment B, Hsb: Inoculated library of experiment B. The Fisher exact test was performed with Bonferroni correction. (PDF 63 KB)

Additional file 2: Table S2: Predicted targets of novel miRNAs using *psRNA Target*. All novel miRNA sequences were denominated Zma_miR_Seq following the number, varying from 01 to 15. (PDF 236 KB)

Additional file 3: Figure S3: Predicted precursor structure of novel miRNAs class I identified. The maize mature miRNA (green), miRNA* (pink) were illustrated in pre-miRNA with chromosome and locus information based in the maize genome v.2. All novel miRNA sequences were denominated Zma_miR_Seq following the number, varying from 01 to 15. (PDF 413 KB)

Additional file 4: Figure S4: CViT image of the B73 assembly aligned with precursor of novel miRNAs. The POPcorn website (http://popcorn.maizegdb.org/main/index.php) was used. All novel miRNA sequences were denominated Zma_miR_Seq following the number, varying from 01 to 15. (PDF 454 KB)

Additional file 5: Figure S5: Analysis of siRNA and methylation profile at the splice variant of GRMZM2G487629 gene. (A) Localization of GRMZM2G487629 in chromosome 1 and two possible splice variants from this gene (boxes represent exons, and lines are introns). (B) Position of siRNA aligned in GRMZM2G487629_T02. The two peaks, blue and green, are siRNA non-redundant sequences from inoculated and control libraries, respectively. (C) Four data tracks show methylation levels for B73 in different sequence contexts. Methylation levels are displayed on a scale from 0 to 1. (PDF 115 KB)
