# Supplementary material for: Genome-wide identification of microRNA and siRNA responsive to endophytic beneficial diazotrophic bacteria in maize
Source: BMC Genomics. 2014 Sep 6;15(1):766. doi: 10.1186/1471-2164-15-766 (PMC4168055; doi:10.1186/1471-2164-15-766)
Supplement: Supplementary file 1 — Additional file 1: Table S1: Differential expression of conserved miRNAs in replicates of libraries. The number of reads found in each library was normalized per million, and the log2 (Hsb/CTb) was calculated. CTb: control library of experiment B, Hsb: Inoculated library of experiment B. The Fisher exact test was performed with Bonferroni correction. (PDF 63 KB) [file 12864_2014_6444_MOESM1_ESM.pdf]

Additional file Table S1: **Differential expression of conserved miRNAs in replicates of libraries.** The number of reads found in each library was normalized per million, and the log2 (Hsb/CTb) was calculated. CTb: control library of experiment B, Hsb: Inoculated library of experiment B. The Fisher exact test was performed with Bonferroni correction.

miRNA	CTb	Hsb	Log 2 (Hsb/CTb)	*p*-value
miR156	3604.31	5596.72	0.63[a]	0.000000
miR159	2545.64	19860.96	2.96[a]	0.000000
miR160	24.28	220.32	3.18	0.000000
miR162	140.11	54.96	-1.35	0.000000
miR164	4.74	431.65	6.51	0.000000
miR166	105248.63	1555.54	-6.08	0.000000
miR167	1007.11	807.38	-0.32[a]	0.000000
miR168	5929.27	4340.16	-0.45	0.000000
miR169	73.64	66.33	-0.15[a]	0.278277
miR171	171.20	852.40	2.32[a]	0.000000
miR172	3.12	39.80	3.67[a]	0.000000
miR319	2147.86	1006.39	-1.09	0.000000
miR3630	116.99	0.00	-[a]	0.000000
miR390	174.90	94.29	-0.89[a]	0.000000
miR393	78.49	38.85	-1.01 [a]	0.000000
miR394	30.29	58.75	0.96 [a]	0.000000
miR396	2230.17	424.07	-2.39	0.000000
miR397	2.54	55.91	4.46 [a]	0.000000
miR398	49.71	272.44	2.45 [a]	0.000000
miR408	162.30	603.64	1.90 [a]	0.000000
miR444	429.57	162.52	-1.40	0.000000
miR528	4.97	211.80	5.41 [a]	0.000000
miR529	44.04	4.26	-3.37	0.000000
miR827	16.18	32.69	1.01 [a]	0.000005
miR858	2.54	135.04	5.73	0.000000

[a] same regulation profile showed in analysis of libraries from samples of experiment A.
