# Supplementary material for: Genome-wide identification of microRNA and siRNA responsive to endophytic beneficial diazotrophic bacteria in maize
Source: BMC Genomics. 2014 Sep 6;15(1):766. doi: 10.1186/1471-2164-15-766 (PMC4168055; doi:10.1186/1471-2164-15-766)
Supplement: Supplementary file 2 — Additional file 2: Table S2: Predicted targets of novel miRNAs using psRNA Target. All novel miRNA sequences were denominated Zma_miR_Seq following the number, varying from 01 to 15. (PDF 236 KB) [file 12864_2014_6444_MOESM2_ESM.pdf]

Additional file Table S2: **Predicted targets of novel miRNAs using *psRNA Target*.** All novel miRNAs sequence were denominated Zma_miR_Seq following the number, varying 01 to 15.

miRNA	Target_Acc.	Target Description
Zma_miR_Seq1	GRMZM2G559586_T01	UniRef90 blastp 11-8-2010: No significant hits (1e-5)
	GRMZM2G165597_T01	UniRef90 blastp 11-8-2010: B8A9K1_ORYSI Putative uncharacterized protein n=1 (Oryza sativa subsp. indica) Exp=0; maizesequence.org: UniGene:Zm.28119; TSA: Zea mays contig18840 mRNA sequence | UniGene:Zm.112943; Transcribed locus | GO:0009405; pathogenesis | GO:0006468; protein amino acid phosphorylation | GO:0005576; extracellular region | GO:0005524; ATP binding | GO:0004674; protein serine/threonine kinase activity | GO:0004672; protein kinase activity
	GRMZM2G132019_T02	UniRef90 blastp 11-8-2010: C5WZV7_SORBI Putative uncharacterized protein Sb01g048230 n=3 (Andropogoneae) Exp=3e-57; maizesequence.org: GO:0005509; calcium ion binding
	GRMZM2G567647_T01	UniRef90 blastp 11-8-2010: No significant hits (1e-5)
	GRMZM2G061702_T07	UniRef90 blastp 11-8-2010: C0PHR9_MAIZE Putative uncharacterized protein n=2 (Zea mays) Exp=3e-79; maizesequence.org: Uniprot/SPTREMBL:C4JBU0; Putative uncharacterized protein | Uniprot/SPTREMBL:B4G0J8; Putative uncharacterized protein | UniGene:Zm.144260; TSA: Zea mays contig56593 mRNA sequence | RefSeq_peptide:NP_001142210; hypothetical protein LOC100274378 | EntrezGene:100274378; LOC100274378
	GRMZM2G061702_T03	UniRef90 blastp 11-8-2010: C0PHR9_MAIZE Putative uncharacterized protein n=2 (Zea mays) Exp=9e-89; maizesequence.org: Uniprot/SPTREMBL:C0PHR9; Putative uncharacterized protein | Uniprot/SPTREMBL:B4G0J8; Putative uncharacterized protein | UniGene:Zm.68046; LOC100274378 | UniGene:Zm.144260; TSA: Zea mays contig56593 mRNA sequence | RefSeq_peptide:NP_001142210; hypothetical protein LOC100274378 | EntrezGene:100274378; LOC100274378

	GRMZM2G080912_T01	UniRef90 blastp 11-8-2010: C0PH10_MAIZE Putative uncharacterized protein n=1 (Zea mays) Exp=0; maizesequence.org: Uniprot/SPTREMBL:C0PH10; Putative uncharacterized protein | UniGene:Zm.20393; Hypothetical protein LOC100383562 | RefSeq_peptide:NP_001169681; hypothetical protein LOC100383562 | RefSeq_dna:NM_001176210; hypothetical protein LOC100383562 (LOC100383562) mRNA | EntrezGene:100383562; hypothetical protein LOC100383562
	GRMZM2G551737_T01	UniRef90 blastp 11-8-2010: No significant hits (1e-5)
	GRMZM2G539278_T01	UniRef90 blastp 11-8-2010: No significant hits (1e-5); maizesequence.org: UniGene:Zm.113829; Transcribed locus
	GRMZM2G575130_T01	UniRef90 blastp 11-8-2010: No significant hits (1e-5); maizesequence.org: UniGene:Zm.107904; Transcribed locus
	GRMZM2G515206_T01	UniRef90 blastp 11-8-2010: No significant hits (1e-5)
	GRMZM2G179715_T05	UniRef90 blastp 11-8-2010: No significant hits (1e-5)
	GRMZM2G179715_T02	UniRef90 blastp 11-8-2010: C5XJF1_SORBI Putative uncharacterized protein Sb03g047450 n=1 (Sorghum bicolor) Exp=1e-26; maizesequence.org: GO:0003676; nucleic acid binding | GO:0003723; RNA binding
	GRMZM2G179715_T01	UniRef90 blastp 11-8-2010: C5XJF1_SORBI Putative uncharacterized protein Sb03g047450 n=1 (Sorghum bicolor) Exp=4e-35; maizesequence.org: UniGene:Zm.72715; TSA: Zea mays contig07741 mRNA sequence | GO:0003723; RNA binding | GO:0003676; nucleic acid binding
	GRMZM2G179715_T04	UniRef90 blastp 11-8-2010: C5XJF1_SORBI Putative uncharacterized protein Sb03g047450 n=1 (Sorghum bicolor) Exp=2e-23; maizesequence.org: GO:0003676; nucleic acid binding | GO:0003723; RNA binding
	GRMZM2G179715_T03	UniRef90 blastp 11-8-2010: No significant hits (1e-5)

	GRMZM2G574124_T01	UniRef90 blastp 11-8-2010: No significant hits (1e-5); maizesequence.org: UniGene:Zm.141078; TSA: Zea mays contig11636 mRNA sequence
	GRMZM2G589781_T01	UniRef90 blastp 11-8-2010: No significant hits (1e-5)
	GRMZM5G836389_T03	UniRef90 blastp 11-8-2010: No significant hits (1e-5)
	GRMZM2G319969_T02	UniRef90 blastp 11-8-2010: No significant hits (1e-5); maizesequence.org: Uniprot/SPTREMBL:B6TNW1; Ubiquitin-like protein 5 | Uniprot/SPTREMBL:B4FFL1; Putative uncharacterized proteinUbiquitin-like protein 5 ; | UniGene:Zm.154099; TSA: Zea mays contig35097 mRNA sequence | RefSeq_peptide:NP_001150362; ubiquitin-like protein 5 | RefSeq_dna:NM_001156890; ubiquitin-like protein 5 (LOC100283992) mRNA | EntrezGene:100283992; ubiquitin-like protein 5
	GRMZM5G897980_T01	UniRef90 blastp 11-8-2010: No significant hits (1e-5); maizesequence.org: UniGene:Zm.138846; Clone 220067 mRNA sequence | UniGene:Zm.78914; LOC100280129 | UniGene:Zm.9771; Transcribed locus
	GRMZM2G319969_T01	UniRef90 blastp 11-8-2010: No significant hits (1e-5); maizesequence.org: Uniprot/SPTREMBL:B6TNW1; Ubiquitin-like protein 5 | Uniprot/SPTREMBL:B4FFL1; Putative uncharacterized proteinUbiquitin-like protein 5 ; | RefSeq_peptide:NP_001150362; ubiquitin-like protein 5 | EntrezGene:100283992; ubiquitin-like protein 5
	GRMZM5G897980_T02	UniRef90 blastp 11-8-2010: No significant hits (1e-5); maizesequence.org: Uniprot/SPTREMBL:C4JB27; Putative uncharacterized protein | UniGene:Zm.9771; Transcribed locus | UniGene:Zm.78914; LOC100280129 | UniGene:Zm.156647; Full-length cDNA clone ZM_BFb0366F02
	GRMZM5G897980_T03	UniRef90 blastp 11-8-2010: No significant hits (1e-5); maizesequence.org: UniGene:Zm.78914; LOC100280129 | UniGene:Zm.9771; Transcribed locus
Zma_miR_Seq2	GRMZM2G089936_T01	UniRef90 blastp 11-8-2010: C5Y329_SORBI Putative uncharacterized protein Sb05g000810 n=1 (Sorghum bicolor) Exp=3e-111

Zma_miR_Seq3	GRMZM2G009633_T07	UniRef90 blastp 11-8-2010: B4FV05_MAIZE Putative uncharacterized protein n=1 (Zea mays) Exp=2e-130; maizesequence.org: Uniprot/SPTREMBL:B4FV05; Putative uncharacterized protein
	GRMZM2G009633_T10	UniRef90 blastp 11-8-2010: B4FV05_MAIZE Putative uncharacterized protein n=1 (Zea mays) Exp=5e-57
Zma_miR_Seq4	GRMZM2G135790_T04	UniRef90 blastp 11-8-2010: B7ZY08_MAIZE Putative uncharacterized protein n=1 (Zea mays) Exp=2e-90; maizesequence.org: Uniprot/SPTREMBL:B7ZY08; Putative uncharacterized protein | UniGene:Zm.27432; Hypothetical protein LOC100279414 | RefSeq_peptide:NP_001145898; hypothetical protein LOC100279414 | RefSeq_dna:NM_001152426; hypothetical protein LOC100279414 (LOC100279414) mRNA | EntrezGene:100279414; hypothetical protein LOC100279414
	GRMZM2G130356_T01	UniRef90 blastp 11-8-2010: B4FL21_MAIZE Putative uncharacterized protein n=2 (Zea mays) Exp=4e-151; maizesequence.org: Uniprot/SPTREMBL:B6T3C0; Putative uncharacterized protein | Uniprot/SPTREMBL:B4FL21; Putative uncharacterized protein | RefSeq_peptide:NP_001143393; hypothetical protein LOC100276031 | RefSeq_dna:NM_001149921; LOC100276031 (pco101788) mRNA | EntrezGene:100276031; LOC100276031
Zma_miR_Seq5	GRMZM2G063792_T01	UniRef90 blastp 11-8-2010: Q2QRH9_ORYSJ Protein kinase AFC1, putative, expressed n=2 (Oryza sativa) Exp=2e-167; maizesequence.org: Uniprot/SPTREMBL:B8A3P5; Putative uncharacterized protein | UniGene:Zm.155466; Transcribed locus weakly similar to XP_002463671.1 hypothetical protein SORBIDRAFT_01g003990 [Sorghum bicolor] | RefSeq_peptide:NP_001146808; hypothetical protein LOC100280413 | EntrezGene:100280413; LOC100280413
	GRMZM2G179308_T02	UniRef90 blastp 11-8-2010: B6TN85_MAIZE Putative uncharacterized protein n=2 (Zea mays) Exp=2e-127; maizesequence.org: Uniprot/SPTREMBL:B4FDM7; Putative uncharacterized protein | RefSeq_peptide:NP_001131698; hypothetical protein LOC100193059 | GO:0006468; protein amino acid phosphorylation | GO:0005524; ATP binding | GO:0004674; protein serine/threonine kinase activity | EntrezGene:100193059; hypothetical protein LOC100193059

	GRMZM2G063792_T02	UniRef90 blastp 11-8-2010: A7XW60_SORBI Lammer-type protein kinase n=1 (Sorghum bicolor) Exp=3e-121; maizesequence.org: EntrezGene:100280413; LOC100280413 | RefSeq_dna:NM_001153336; LOC100280413 (cl31185_1) mRNA | RefSeq_peptide:NP_001146808; hypothetical protein LOC100280413 | UniGene:Zm.155466; Transcribed locus weakly similar to XP_002463671.1 hypothetical protein SORBIDRAFT_01g003990 [Sorghum bicolor] | Uniprot/SPTREMBL:B8A3P5; Putative uncharacterized protein
	GRMZM2G063792_T03	UniRef90 blastp 11-8-2010: A7XW60_SORBI Lammer-type protein kinase n=1 (Sorghum bicolor) Exp=3e-121; maizesequence.org: UniGene:Zm.94274; LOC100280413 | UniGene:Zm.155466; Transcribed locus weakly similar to XP_002463671.1 hypothetical protein SORBIDRAFT_01g003990 [Sorghum bicolor] | GO:0006468; protein amino acid phosphorylation | GO:0005524; ATP binding | GO:0004674; protein serine/threonine kinase activity
	GRMZM2G153369_T01	UniRef90 blastp 11-8-2010: C5X7T8_SORBI Putative uncharacterized protein Sb02g032580 n=2 (Andropogoneae) Exp=2e-35; maizesequence.org: Uniprot/SPTREMBL:B6SPH2; Putative uncharacterized protein | UniGene:Zm.144407; TSA: Zea mays contig55400 mRNA sequence | GO:0016021; integral to membrane
	GRMZM2G153369_T02	UniRef90 blastp 11-8-2010: C5X7T8_SORBI Putative uncharacterized protein Sb02g032580 n=2 (Andropogoneae) Exp=5e-09; maizesequence.org: GO:0016021; integral to membrane
	GRMZM2G117612_T01	UniRef90 blastp 11-8-2010: B6TXY1_MAIZE Protein binding protein n=3 (Andropogoneae) Exp=0; maizesequence.org: UniGene:Zm.141823; TSA: Zea mays contig20249 mRNA sequence | UniGene:Zm.124074; TSA: Zea mays contig25228 mRNA sequence | GO:0008270; zinc ion binding | GO:0005515; protein binding
	GRMZM2G439350_T01	UniRef90 blastp 11-8-2010: No significant hits (1e-5); maizesequence.org: GO:0004672; protein kinase activity | GO:0004674; protein serine/threonine kinase activity | GO:0005524; ATP binding | GO:0006468; protein amino acid phosphorylation | UniGene:Zm.100550; Transcribed locus weakly similar to NP_001043977.1 Os01g0699100 [Oryza sativa Japonica Group] | UniGene:Zm.128343; Clone 335251 mRNA sequence

Zma_miR_Seq6	GRMZM2G081114_T01	UniRef90 blastp 11-8-2010: B4FS10_MAIZE Putative uncharacterized protein n=1 (Zea mays) Exp=1e-16; maizesequence.org: UniGene:Zm.144280; TSA: Zea mays contig56400 mRNA sequence | Uniprot/SPTREMBL:B4FS10; Putative uncharacterized protein
	GRMZM2G032528_T02	UniRef90 blastp 11-8-2010: C0PGD9_MAIZE Putative uncharacterized protein n=1 (Zea mays) Exp=8e-141; maizesequence.org: Uniprot/SPTREMBL:C0PGD9; Putative uncharacterized protein | UniGene:Zm.122426; Hypothetical protein LOC100276404 | RefSeq_peptide:NP_001143680; hypothetical protein LOC100276404 | RefSeq_dna:NM_001150208; hypothetical protein LOC100276404 (LOC100276404) mRNA
	GRMZM2G118917_T02	UniRef90 blastp 11-8-2010: C5X0K2_SORBI Putative uncharacterized protein Sb01g021230 n=1 (Sorghum bicolor) Exp=0; maizesequence.org: GO:0008408; 3-prime-5-prime exonuclease activity | GO:0006139; nucleobase nucleoside nucleotide and nucleic acid metabolic process | GO:0005622; intracellular | GO:0003676; nucleic acid binding
	GRMZM2G118917_T01	UniRef90 blastp 11-8-2010: C5X0K2_SORBI Putative uncharacterized protein Sb01g021230 n=1 (Sorghum bicolor) Exp=0; maizesequence.org: EntrezGene:100383056; hypothetical protein LOC100383056 | Uniprot/SPTREMBL:C0PCP0; Putative uncharacterized protein | Uniprot/SPTREMBL:C0HIE0; Putative uncharacterized protein | UniGene:Zm.28005; Hypothetical protein LOC100383056 | RefSeq_peptide:NP_001169203; hypothetical protein LOC100383056 | RefSeq_dna:NM_001175732; hypothetical protein LOC100383056 (LOC100383056) mRNA | GO:0008408; 3-prime-5-prime exonuclease activity | GO:0006139; nucleobase nucleoside nucleotide and nucleic acid metabolic process | GO:0005622; intracellular | GO:0003676; nucleic acid binding
Zma_miR_Seq7	GRMZM2G028208_T01	UniRef90 blastp 11-8-2010: No significant hits (1e-5)
	GRMZM2G374169_T01	UniRef90 blastp 11-8-2010: C5XB14_SORBI Putative uncharacterized protein Sb02g004455 (Fragment) n=1 (Sorghum bicolor) Exp=2e-40; maizesequence.org: GO:0016021; integral to membrane | GO:0007186; G-protein

		coupled receptor protein signaling pathway | GO:0004939; beta-adrenergic receptor activity | GO:0003676; nucleic acid binding
	GRMZM2G138259_T01	UniRef90 blastp 11-8-2010: C5YKU9_SORBI Putative uncharacterized protein Sb07g020180 n=1 (Sorghum bicolor) Exp=0; maizesequence.org: GO:0003677; DNA binding | UniGene:Zm.25847; Transcribed locus moderately similar to XP_002444325.1 hypothetical protein SORBIDRAFT_07g020180 [Sorghum bicolor]
	AC186885.3_FGT001	UniRef90 blastp 11-8-2010: No significant hits (1e-5)
	AC188753.3_FGT004	UniRef90 blastp 11-8-2010: No significant hits (1e-5)
	GRMZM2G099609_T01	UniRef90 blastp 11-8-2010: B4FLC2_MAIZE Putative uncharacterized protein n=1 (Zea mays) Exp=3e-98; maizesequence.org: GO:0016021; integral to membrane | GO:0016020; membrane | GO:0007186; G-protein coupled receptor protein signaling pathway
	GRMZM2G086983_T01	UniRef90 blastp 11-8-2010: No significant hits (1e-5); maizesequence.org: RefSeq_dna:NM_001149579; hypothetical protein LOC100275520 (LOC100275520) mRNA | UniGene:Zm.84900; Hypothetical protein LOC100275520
Zma_miR_Seq8	GRMZM5G824689_T01	UniRef90 blastp 11-8-2010: No significant hits (1e-5); maizesequence.org: EntrezGene:100384429; hypothetical protein LOC100384429 | RefSeq_peptide:NP_001170437; hypothetical protein LOC100384429 | Uniprot/SPTREMBL:C0PP74; Putative uncharacterized protein
	GRMZM2G146940_T01	UniRef90 blastp 11-8-2010: Q6Z4F3_ORYSJ Os07g0187700 protein n=4 (Poaceae) Exp=3e-28
	GRMZM2G029737_T01	UniRef90 blastp 11-8-2010: No significant hits (1e-5)
	GRMZM2G317272_T01	UniRef90 blastp 11-8-2010: No significant hits (1e-5)

	GRMZM2G105176_T01	UniRef90 blastp 11-8-2010: No significant hits (1e-5); maizesequence.org: UniGene:Zm.17035; Transcribed locus
	AC194898.3_FGT003	UniRef90 blastp 11-8-2010: C5Y3V9_SORBI Putative uncharacterized protein Sb05g020880 n=1 (Sorghum bicolor) Exp=1e-40; maizesequence.org: GO:0016020; membrane | GO:0006810; transport | GO:0005215; transporter activity
	GRMZM2G506660_T01	UniRef90 blastp 11-8-2010: B6U683_MAIZE ATP binding protein n=1 (Zea mays) Exp=2e-73; maizesequence.org: EntrezGene:100285538; ATP binding protein | GO:0003676; nucleic acid binding | RefSeq_dna:NM_001158429; ATP binding protein (LOC100285538) mRNA | RefSeq_peptide:NP_001151901; ATP binding protein | UniGene:Zm.20574; ATP binding protein | Uniprot/SPTREMBL:B6U683; ATP binding proteinPutative uncharacterized protein ;
	GRMZM2G071113_T01	UniRef90 blastp 11-8-2010: B6TDI2_MAIZE F-box domain containing protein n=5 (Andropogoneae) Exp=4e-128; maizesequence.org: Uniprot/SPTREMBL:B6UEE7; Putative uncharacterized protein | Uniprot/SPTREMBL:B6TAK8; F-box domain containing protein | Uniprot/SPTREMBL:B4FC83; Putative uncharacterized protein | UniGene:Zm.95766; Hypothetical protein LOC100192671 | RefSeq_peptide:NP_001131351; hypothetical protein LOC100192671 | RefSeq_dna:NM_001137879; hypothetical protein LOC100192671 (LOC100192671) mRNA | EntrezGene:100192671; hypothetical protein LOC100192671
	GRMZM2G058021_T01	UniRef90 blastp 11-8-2010: B6TZQ4_MAIZE Putative uncharacterized protein n=1 (Zea mays) Exp=2e-126; maizesequence.org: Uniprot/SPTREMBL:B6TZQ4; Putative uncharacterized protein | UniGene:Zm.91367; Hypothetical protein LOC100277766 | RefSeq_peptide:NP_001144724; hypothetical protein LOC100277766 | RefSeq_dna:NM_001151252; hypothetical protein LOC100277766 (LOC100277766) mRNA | EntrezGene:100277766; hypothetical protein LOC100277766
Zma_miR_Seq09	GRMZM2G515535_T01	UniRef90 blastp 11-8-2010: No significant hits (1e-5)
	GRMZM2G538544_T01	UniRef90 blastp 11-8-2010: No significant hits (1e-5)

	GRMZM2G533989_T01	UniRef90 blastp 11-8-2010: No significant hits (1e-5)
	GRMZM2G151151_T01	UniRef90 blastp 11-8-2010: No significant hits (1e-5); maizesequence.org: UniGene:Zm.128600; Clone 1529191 mRNA sequence
	GRMZM2G150264_T01	UniRef90 blastp 11-8-2010: B6U841_MAIZE Galactosylgalactosylxylosylprotein 3-beta-glucuronosyltransferase 1 n=2 (Zea mays) Exp=0; maizesequence.org: UniGene:Zm.153536; TSA: Zea mays contig37948 mRNA sequence | UniGene:Zm.29623; LOC100285679 | Uniprot/SPTREMBL:B6U841; Galactosylgalactosylxylosylprotein 3-beta-glucuronosyltransferase 1 | Uniprot/SPTREMBL:B8A370; Putative uncharacterized protein | UniGene:Zm.147416; TSA: Zea mays contig26269 mRNA sequence | RefSeq_peptide:NP_001152042; LOC100285679 | RefSeq_dna:NM_001158570; LOC100285679 (TIDP2711) mRNA | GO:0016020; membrane | GO:0015018; galactosylgalactosylxylosylprotein 3-beta-glucuronosyltransferase activity | EntrezGene:100285679; LOC100285679
	GRMZM2G406101_T05	UniRef90 blastp 11-8-2010: C5Z2M2_SORBI Putative uncharacterized protein Sb10g031140 n=3 (Andropogoneae) Exp=0; maizesequence.org: Uniprot/SPTREMBL:C0P3W7; Putative uncharacterized protein | Uniprot/SPTREMBL:B7ZYY8; Putative uncharacterized protein | Uniprot/SPTREMBL:B6SSS6; Putative uncharacterized protein | UniGene:Zm.144390; TSA: Zea mays contig55510 mRNA sequence | RefSeq_peptide:NP_001146062; hypothetical protein LOC100279593 | EntrezGene:100279593; hypothetical protein LOC100279593
Zma_miR_Seq10	GRMZM2G059214_T01	UniRef90 blastp 11-8-2010: C5YT61_SORBI Putative uncharacterized protein Sb08g005060 n=1 (Sorghum bicolor) Exp=0; maizesequence.org: UniGene:Zm.127082; TSA: Zea mays contig13060 mRNA sequence | GO:0006468; protein amino acid phosphorylation | GO:0005524; ATP binding | GO:0005515; protein binding | GO:0004674; protein serine/threonine kinase activity | GO:0004672; protein kinase activity
	GRMZM2G059214_T02	UniRef90 blastp 11-8-2010: C5YT61_SORBI Putative uncharacterized protein Sb08g005060 n=1 (Sorghum bicolor) Exp=9e-149; maizesequence.org: GO:0006468; protein amino acid phosphorylation | GO:0005524; ATP binding | GO:0004674; protein serine/threonine kinase activity | GO:0004672; protein kinase activity

Zma_miR_Seq11	GRMZM2G470258_T01	UniRef90 blastp 11-8-2010: No significant hits (1e-5)
	GRMZM2G470242_T01	UniRef90 blastp 11-8-2010: No significant hits (1e-5)
	GRMZM2G143142_T01	UniRef90 blastp 11-8-2010: No significant hits (1e-5); maizesequence.org: UniGene:Zm.137370; Hypothetical protein LOC100279645 | RefSeq_dna:NM_001152636; hypothetical protein LOC100279645 (LOC100279645) mRNA
	GRMZM5G891837_T01	UniRef90 blastp 11-8-2010: No significant hits (1e-5)
	GRMZM2G098305_T01	UniRef90 blastp 11-8-2010: B6U5B2_MAIZE Single-stranded DNA-binding protein n=2 (Zea mays) Exp=2e-106; maizesequence.org: Uniprot/SPTREMBL:B6U5B2; Single-stranded DNA-binding protein | Uniprot/SPTREMBL:B4FRF4; Putative uncharacterized proteinSingle-stranded DNA-binding protein ; | RefSeq_peptide:NP_001151839; single-stranded DNA-binding protein | RefSeq_dna:NM_001158367; single-stranded DNA-binding protein (LOC100285474) mRNA | GO:0003697; single-stranded DNA binding | EntrezGene:100285474; single-stranded DNA-binding protein
	GRMZM2G047139_T03	UniRef90 blastp 11-8-2010: B6TZ91_MAIZE Esterase n=2 (Andropogoneae) Exp=5e-24; maizesequence.org: UniGene:Zm.144285; TSA: Zea mays contig56349 mRNA sequence
	GRMZM2G108759_T01	UniRef90 blastp 11-8-2010: No significant hits (1e-5)
	GRMZM2G702613_T02	UniRef90 blastp 11-8-2010: C5Z8T6_SORBI Putative uncharacterized protein Sb10g028590 n=1 (Sorghum bicolor) Exp=4e-13
	GRMZM2G324340_T01	UniRef90 blastp 11-8-2010: No significant hits (1e-5)
	GRMZM2G324340_T02	UniRef90 blastp 11-8-2010: No significant hits (1e-5)

	GRMZM2G702613_T01	UniRef90 blastp 11-8-2010: C5Z8T6_SORBI Putative uncharacterized protein Sb10g028590 n=1 (Sorghum bicolor) Exp=3e-14
	GRMZM2G704020_T01	UniRef90 blastp 11-8-2010: No significant hits (1e-5)
Zma_miR_Seq12	GRMZM2G107457_T01	UniRef90 blastp 11-8-2010: C5XAA0_SORBI Putative uncharacterized protein Sb02g003680 n=1 (Sorghum bicolor) Exp=0; maizesequence.org: UniGene:Zm.10391; TSA: Zea mays contig38996 mRNA sequence | MaizeGDB_GenBank:AY105677; | GO:0005622; intracellular | GO:0003725; double-stranded RNA binding
	GRMZM2G042723_T01	UniRef90 blastp 11-8-2010: No significant hits (1e-5); maizesequence.org: UniGene:Zm.140722; Hypothetical protein LOC100383619 | RefSeq_peptide:NP_001169738; hypothetical protein LOC100383619 | RefSeq_dna:NM_001176267; hypothetical protein LOC100383619 (LOC100383619) mRNA
	GRMZM5G855035_T01	UniRef90 blastp 11-8-2010: No significant hits (1e-5); maizesequence.org: UniGene:Zm.149795; TSA: Zea mays contig15448 mRNA sequence | UniGene:Zm.80596; Full-length cDNA clone ZM_BFc0062H03 | Uniprot/SPTREMBL:C4J4N5; Putative uncharacterized protein
Zma_miR_Seq13	GRMZM5G824689_T01	UniRef90 blastp 11-8-2010: No significant hits (1e-5); maizesequence.org: EntrezGene:100384429; hypothetical protein LOC100384429 | RefSeq_peptide:NP_001170437; hypothetical protein LOC100384429 | Uniprot/SPTREMBL:C0PP74; Putative uncharacterized protein
	GRMZM2G178650_T05	UniRef90 blastp 11-8-2010: No significant hits (1e-5)
	GRMZM2G178650_T04	UniRef90 blastp 11-8-2010: No significant hits (1e-5); maizesequence.org: UniGene:Zm.155382; Transcribed locus strongly similar to NP_001137123.1 hypothetical protein LOC100217304 [Zea mays]
	GRMZM2G178650_T03	UniRef90 blastp 11-8-2010: C5WYR7_SORBI Putative uncharacterized protein Sb01g034550 n=1 (Sorghum bicolor) Exp=0; maizesequence.org: UniGene:Zm.145110; TSA: Zea mays contig49845 mRNA sequence

	GRMZM2G178650_T02	UniRef90 blastp 11-8-2010: C5WYR7_SORBI Putative uncharacterized protein Sb01g034550 n=1 (Sorghum bicolor) Exp=0; maizesequence.org: Uniprot/SPTREMBL:B4FMG9; Putative uncharacterized protein | UniGene:Zm.94652; LOC100217304 | UniGene:Zm.145110; TSA: Zea mays contig49845 mRNA sequence | RefSeq_peptide:NP_001137123; hypothetical protein LOC100217304 | RefSeq_dna:NM_001143651; LOC100217304 (si486041f06) mRNA | EntrezGene:100217304; LOC100217304
	GRMZM2G178650_T01	UniRef90 blastp 11-8-2010: C5WYR7_SORBI Putative uncharacterized protein Sb01g034550 n=1 (Sorghum bicolor) Exp=0; maizesequence.org: UniGene:Zm.145110; TSA: Zea mays contig49845 mRNA sequence
	GRMZM2G073222_T01	UniRef90 blastp 11-8-2010: No significant hits (1e-5); maizesequence.org: GO:0006412; translation | GO:0005840; ribosome | GO:0005622; intracellular | GO:0003735; structural constituent of ribosome | GO:0003723; RNA binding
	GRMZM2G448151_T01	UniRef90 blastp 11-8-2010: RR3_ORYSJ 30S ribosomal protein S3, chloroplastic n=19 (Poaceae) Exp=5e-120; maizesequence.org: Uniprot/SWISSPROT:P06586; 30S ribosomal protein S3 chloroplastic | UniGene:Zm.52251; Transcribed locus | UniGene:Zm.115900; Transcribed locus | RefSeq_peptide:NP_043062; ribosomal protein S3 | GO:0015935; small ribosomal subunit | GO:0006412; translation | GO:0005840; ribosome | GO:0005622; intracellular | GO:0003735; structural constituent of ribosome | GO:0003723; RNA binding | EntrezGene:845237; 30S ribosomal protein S3
	GRMZM2G100336_T01	UniRef90 blastp 11-8-2010: Q6R9C6_MAIZE Putative uncharacterized protein orf247-ct n=1 (Zea mays) Exp=1e-64; maizesequence.org: GO:0015935; small ribosomal subunit | GO:0006412; translation | GO:0005840; ribosome | GO:0005622; intracellular | GO:0003735; structural constituent of ribosome | GO:0003723; RNA binding
	GRMZM2G074479_T01	UniRef90 blastp 11-8-2010: UBC1_ARATH Ubiquitin-conjugating enzyme E2 1 n=60 (Embryophyta) Exp=2e-83; maizesequence.org: Uniprot/SPTREMBL:B6SGR7; Ubiquitin carrier protein (EC 6.3.2.-) | RefSeq_peptide:NP_001150120; ubiquitin-conjugating enzyme E2-17 kDa | RefSeq_peptide:NP_001149671; ubiquitin-conjugating enzyme E2-17 kDa | RefSeq_peptide:NP_001146868; ubiquitin-conjugating enzyme E2-17

		kDa | GO:0051246; regulation of protein metabolic process | GO:0043687; post-translational protein modification | GO:0019787; small conjugating protein ligase activity | EntrezGene:100283749; ubiquitin-conjugating enzyme E2-17 kDa | EntrezGene:100283297; ubiquitin-conjugating enzyme E2-17 kDa | EntrezGene:100280476; ubiquitin-conjugating enzyme E2-17 kDa
	GRMZM2G074479_T04	UniRef90 blastp 11-8-2010: B6SYW5_MAIZE Putative uncharacterized protein n=1 (Zea mays) Exp=6e-28; maizesequence.org: GO:0051246; regulation of protein metabolic process | GO:0043687; post-translational protein modification | GO:0019787; small conjugating protein ligase activity
	GRMZM2G074479_T03	UniRef90 blastp 11-8-2010: UBC1_ARATH Ubiquitin-conjugating enzyme E2 1 n=60 (Embryophyta) Exp=2e-83; maizesequence.org: Uniprot/SPTREMBL:B6SGR7; Ubiquitin carrier protein (EC 6.3.2.-) | RefSeq_peptide:NP_001150120; ubiquitin-conjugating enzyme E2-17 kDa | RefSeq_peptide:NP_001149671; ubiquitin-conjugating enzyme E2-17 kDa | RefSeq_peptide:NP_001146868; ubiquitin-conjugating enzyme E2-17 kDa | RefSeq_dna:NM_001156648; ubiquitin-conjugating enzyme E2-17 kDa (LOC100283749) mRNA | GO:0051246; regulation of protein metabolic process | GO:0043687; post-translational protein modification | GO:0019787; small conjugating protein ligase activity | EntrezGene:100283749; ubiquitin-conjugating enzyme E2-17 kDa | EntrezGene:100283297; ubiquitin-conjugating enzyme E2-17 kDa | EntrezGene:100280476; ubiquitin-conjugating enzyme E2-17 kDa
	GRMZM2G074479_T06	UniRef90 blastp 11-8-2010: Q10C79_ORYSJ Ubiquitin-conjugating enzyme E2-17 kDa, putative, expressed n=1 (Oryza sativa subsp. japonica) Exp=3e-18; maizesequence.org: GO:0019787; small conjugating protein ligase activity | GO:0043687; post-translational protein modification | GO:0051246; regulation of protein metabolic process | UniGene:Zm.45074; Ubiquitin-conjugating enzyme E2-17 kDa
	GRMZM2G088132_T02	UniRef90 blastp 11-8-2010: No significant hits (1e-5); maizesequence.org: GO:0055085; transmembrane transport | GO:0008308; voltage-gated anion channel activity | GO:0006820; anion transport | GO:0005741; mitochondrial outer membrane

	GRMZM2G172101_T01	UniRef90 blastp 11-8-2010: O82108_MAIZE Seryl-tRNA synthetase n=4 (Andropogoneae) Exp=0; maizesequence.org: RefSeq_dna:NM_001147191; hypothetical protein LOC100272738 (LOC100272738) mRNA | RefSeq_peptide:NP_001105110; seryl-tRNA synthetase1 | RefSeq_peptide:NP_001140663; hypothetical protein LOC100272738 | UniGene:Zm.103342; Hypothetical protein LOC100272738 | UniGene:Zm.49401; Clone 1579927 mRNA sequence | Uniprot/SPTREMBL:B4FQC4; Putative uncharacterized protein | Uniprot/SPTREMBL:B4FRC4; Putative uncharacterized proteinSeryl-tRNA synthetase ; | Uniprot/SPTREMBL:B4FXV6; Putative uncharacterized protein | Uniprot/SPTREMBL:O82108; Seryl-tRNA synthetase (EC 6.1.1.11) | RefSeq_dna:NM_001111640; seryl-tRNA synthetase1 (ser1) mRNA | GO:0006434; seryl-tRNA aminoacylation | EntrezGene:100272738; hypothetical protein LOC100272738 | EntrezGene:541989; seryl-tRNA synthetase1 | GO:0000166; nucleotide binding | GO:0004812; aminoacyl-tRNA ligase activity | GO:0004828; serine-tRNA ligase activity | GO:0005524; ATP binding | GO:0005737; cytoplasm | GO:0006412; translation | GO:0006418; tRNA aminoacylation
Zma_miR_Seq14	GRMZM2G056645_T01	UniRef90 blastp 11-8-2010: No significant hits (1e-5); maizesequence.org: EntrezGene:100381565; hypothetical protein LOC100381565 | RefSeq_dna:NM_001174390; hypothetical protein LOC100381565 (LOC100381565) mRNA | RefSeq_peptide:NP_001167861; hypothetical protein LOC100381565 | UniGene:Zm.138676; Transcribed locus strongly similar to NP_001173640.1 Os03g0744650 [Oryza sativa Japonica Group] | UniGene:Zm.153141; TSA: Zea mays contig39509 mRNA sequence | UniGene:Zm.23427; Hypothetical protein LOC100381565 | Uniprot/SPTREMBL:C0HH26; Putative uncharacterized protein
	GRMZM2G144841_T01	UniRef90 blastp 11-8-2010: B6TGV7_MAIZE TOM2B n=4 (Andropogoneae) Exp=6e-49; maizesequence.org: Uniprot/SPTREMBL:B6SX44; TOM2B | UniGene:Zm.32833; TOM2B | RefSeq_peptide:NP_001148020; TOM2B | RefSeq_dna:NM_001154548; TOM2B (LOC100281629) mRNA | EntrezGene:100281629; TOM2B
	GRMZM5G852776_T01	UniRef90 blastp 11-8-2010: No significant hits (1e-5); maizesequence.org: UniGene:Zm.37095; TSA: Zea mays cultivar W22 CL520Contig1.betc mRNA sequence | GO:0006468; protein amino acid phosphorylation | GO:0005524; ATP binding | GO:0005515; protein binding | GO:0004674; protein serine/threonine kinase activity | GO:0004672; protein kinase activity

	GRMZM2G167758_T02	UniRef90 blastp 11-8-2010: B6SR37_MAIZE Putative uncharacterized protein n=2 (Zea mays) Exp=6e-65; maizesequence.org: Uniprot/SPTREMBL:B6SQ99; Putative uncharacterized protein | UniGene:Zm.7961; Hypothetical protein LOC100275228 | RefSeq_peptide:NP_001142838; hypothetical protein LOC100275228 | RefSeq_dna:NM_001149366; hypothetical protein LOC100275228 (LOC100275228) mRNA | EntrezGene:100275228; hypothetical protein LOC100275228
	GRMZM5G828155_T01	UniRef90 blastp 11-8-2010: No significant hits (1e-5); maizesequence.org: UniGene:Zm.125127; Clone 234839 mRNA sequence
	GRMZM2G136268_T01	UniRef90 blastp 11-8-2010: C5WP96_SORBI Putative uncharacterized protein Sb01g038980 n=1 (Sorghum bicolor) Exp=0
	GRMZM2G136268_T02	UniRef90 blastp 11-8-2010: C5WP96_SORBI Putative uncharacterized protein Sb01g038980 n=1 (Sorghum bicolor) Exp=0; maizesequence.org: UniGene:Zm.125660; TSA: Zea mays contig35986 mRNA sequence
	GRMZM2G050709_T01	UniRef90 blastp 11-8-2010: B4FB05_MAIZE Putative uncharacterized protein n=1 (Zea mays) Exp=0; maizesequence.org: Uniprot/SPTREMBL:B4FB05; Putative uncharacterized protein | UniGene:Zm.120329; Hypothetical protein LOC100192016 | RefSeq_peptide:NP_001130912; hypothetical protein LOC100192016 | RefSeq_dna:NM_001137440; hypothetical protein LOC100192016 (LOC100192016) mRNA | GO:0005529; sugar binding | EntrezGene:100192016; hypothetical protein LOC100192016
	GRMZM2G098331_T01	UniRef90 blastp 11-8-2010: C0HGK9_MAIZE Putative uncharacterized protein n=2 (Zea mays) Exp=1e-124; maizesequence.org: Uniprot/SPTREMBL:B4FCK1; Putative uncharacterized protein | RefSeq_peptide:NP_001131439; hypothetical protein LOC100192771 | RefSeq_dna:NM_001137967; LOC100192771 (AY111680) mRNA | GO:0016020; membrane | GO:0008021; synaptic vesicle | GO:0006810; transport | GO:0005215; transporter activity | EntrezGene:100192771; LOC100192771

	GRMZM2G098331_T02	UniRef90 blastp 11-8-2010: C0HGK9_MAIZE Putative uncharacterized protein n=2 (Zea mays) Exp=1e-176; maizesequence.org: Uniprot/SPTREMBL:C0HGK9; Putative uncharacterized protein | UniGene:Zm.5132; LOC100192771
	GRMZM2G496410_T01	UniRef90 blastp 11-8-2010: No significant hits (1e-5); maizesequence.org: UniGene:Zm.46475; Transcribed locus
Zma_miR_Seq15	GRMZM2G044788_T01	UniRef90 blastp 11-8-2010: B6SLC8_MAIZE Putative uncharacterized protein n=1 (Zea mays) Exp=6e-43; maizesequence.org: Uniprot/SPTREMBL:B6SLC8; Putative uncharacterized protein | UniGene:Zm.152044; TSA: Zea mays contig04626 mRNA sequence | UniGene:Zm.147351; TSA: Zea mays contig26568 mRNA sequence | UniGene:Zm.131538; Hypothetical protein LOC100275041 | UniGene:Zm.131537; Hypothetical protein LOC100274914 | RefSeq_peptide:NP_001142713; hypothetical protein LOC100275041 | RefSeq_peptide:NP_001142637; hypothetical protein LOC100274914 | RefSeq_dna:NM_001149241; hypothetical protein LOC100275041 (LOC100275041) mRNA | RefSeq_dna:NM_001149165; hypothetical protein LOC100274914 (LOC100274914) mRNA | EntrezGene:100274914; hypothetical protein LOC100274914
	GRMZM2G048819_T03	UniRef90 blastp 11-8-2010: C5X9Z8_SORBI Putative uncharacterized protein Sb02g034580 n=1 (Sorghum bicolor) Exp=2e-102; maizesequence.org: UniGene:Zm.126617; Transcribed locus moderately similar to XP_002462926.1 hypothetical protein SORBIDRAFT_02g034580 [Sorghum bicolor] | GO:0008408; 3-prime-5-prime exonuclease activity | GO:0006139; nucleobase nucleoside nucleotide and nucleic acid metabolic process | GO:0005622; intracellular | GO:0003676; nucleic acid binding
	GRMZM2G048819_T02	UniRef90 blastp 11-8-2010: B9FXH1_ORYSJ Putative uncharacterized protein n=2 (Oryza sativa) Exp=0; maizesequence.org: UniGene:Zm.20791; TSA: Zea mays contig26909 mRNA sequence | UniGene:Zm.147341; TSA: Zea mays contig26628 mRNA sequence | UniGene:Zm.125875; Transcribed locus strongly similar to XP_002462926.1 hypothetical protein SORBIDRAFT_02g034580 [Sorghum bicolor] | GO:0008408; 3-prime-5-prime exonuclease activity | GO:0006139; nucleobase nucleoside nucleotide and nucleic acid metabolic process | GO:0005622; intracellular | GO:0003676; nucleic acid binding

	GRMZM2G048819_T01	UniRef90 blastp 11-8-2010: B9FXH1_ORYSJ Putative uncharacterized protein n=2 (Oryza sativa) Exp=0; maizesequence.org: UniGene:Zm.20791; TSA: Zea mays contig26909 mRNA sequence | UniGene:Zm.147341; TSA: Zea mays contig26628 mRNA sequence | UniGene:Zm.125875; Transcribed locus strongly similar to XP_002462926.1 hypothetical protein SORBIDRAFT_02g034580 [Sorghum bicolor] | GO:0008408; 3-prime-5-prime exonuclease activity | GO:0006139; nucleobase nucleoside nucleotide and nucleic acid metabolic process | GO:0005622; intracellular | GO:0003676; nucleic acid binding
	GRMZM2G441284_T01	UniRef90 blastp 11-8-2010: No significant hits (1e-5)
	GRMZM2G414334_T01	UniRef90 blastp 11-8-2010: No significant hits (1e-5)
	AC218985.3_FGT006	UniRef90 blastp 11-8-2010: No significant hits (1e-5)
	GRMZM2G337870_T01	UniRef90 blastp 11-8-2010: No significant hits (1e-5)
	GRMZM2G309787_T02	UniRef90 blastp 11-8-2010: No significant hits (1e-5)
	GRMZM2G405528_T01	UniRef90 blastp 11-8-2010: No significant hits (1e-5)
	GRMZM2G309787_T01	UniRef90 blastp 11-8-2010: No significant hits (1e-5)
	GRMZM2G344414_T01	UniRef90 blastp 11-8-2010: No significant hits (1e-5)
	GRMZM2G011824_T01	UniRef90 blastp 11-8-2010: No significant hits (1e-5)
	AC192368.3_FGT004	UniRef90 blastp 11-8-2010: No significant hits (1e-5)
	GRMZM2G702552_T02	UniRef90 blastp 11-8-2010: C0HHP9_MAIZE Putative uncharacterized protein n=2 (Zea mays) Exp=4e-55
	AC194136.3_FGT003	UniRef90 blastp 11-8-2010: No significant hits (1e-5)

	GRMZM2G002804_T01	UniRef90 blastp 11-8-2010: No significant hits (1e-5)
	GRMZM2G307860_T01	UniRef90 blastp 11-8-2010: No significant hits (1e-5)
	GRMZM2G479061_T01	UniRef90 blastp 11-8-2010: No significant hits (1e-5)
	GRMZM2G098252_T01	UniRef90 blastp 11-8-2010: No significant hits (1e-5)
	AC185119.3_FGT001	UniRef90 blastp 11-8-2010: No significant hits (1e-5)
	GRMZM5G846648_T01	UniRef90 blastp 11-8-2010: No significant hits (1e-5)
	GRMZM2G445681_T01	UniRef90 blastp 11-8-2010: No significant hits (1e-5)
	GRMZM2G399575_T01	UniRef90 blastp 11-8-2010: No significant hits (1e-5)
	GRMZM2G425603_T01	UniRef90 blastp 11-8-2010: No significant hits (1e-5)
