# Supplementary material for: Genome-wide identification of microRNA and siRNA responsive to endophytic beneficial diazotrophic bacteria in maize
Source: BMC Genomics. 2014 Sep 6;15(1):766. doi: 10.1186/1471-2164-15-766 (PMC4168055; doi:10.1186/1471-2164-15-766)

Additional file Figure S3: **Predicted precursor structure of novel miRNAs class I identified.** The maize mature miRNA (green), miRNA* (pink) were illustrated in pre-miRNA with chromosome and locus information based in maize genome v.2. All novel miRNAs sequence were denominated Zma_miR_C following the number, varying 01 to 15.

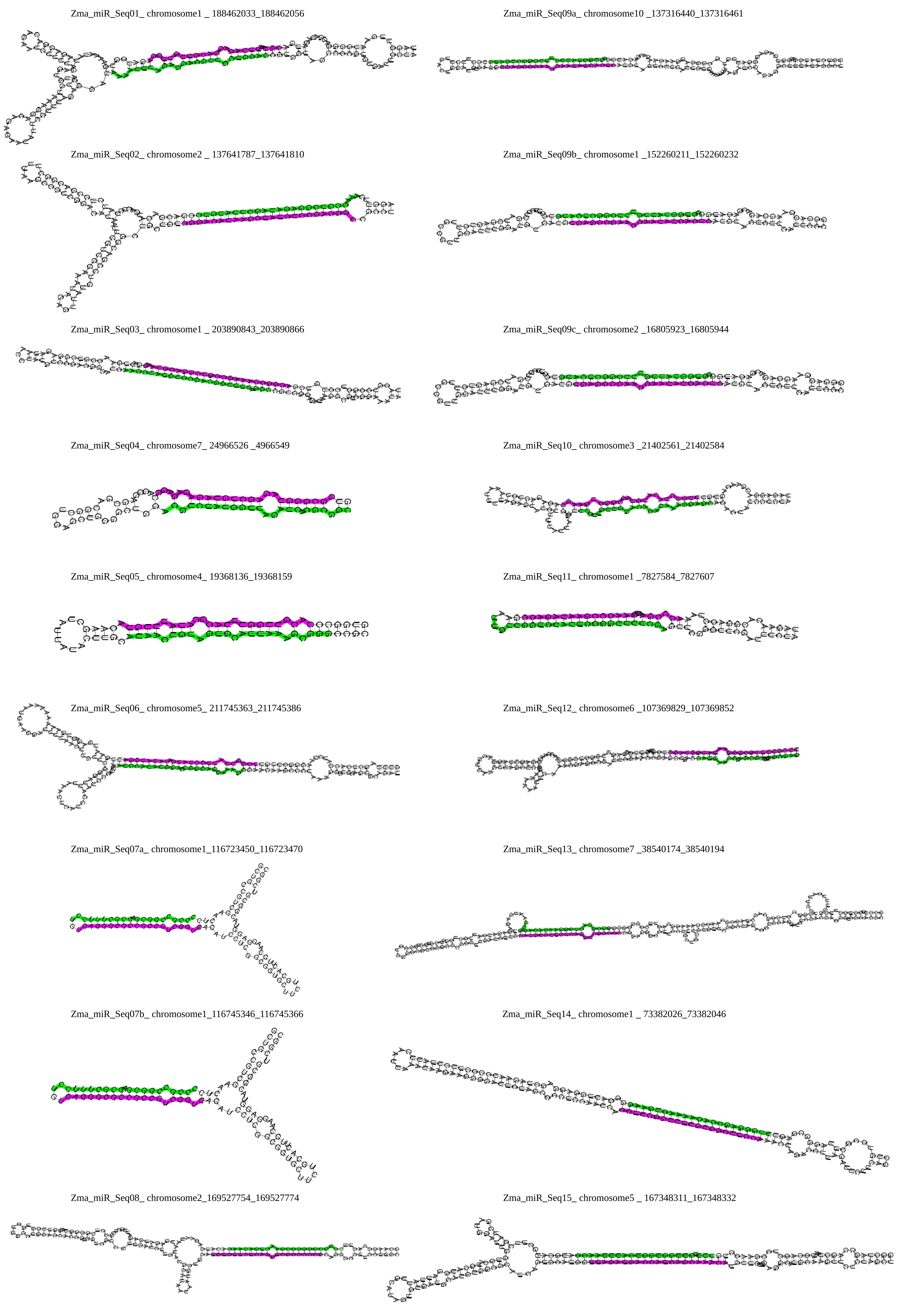

Supplement: Supplementary file 3 — Additional file 3: Figure S3: Predicted precursor structure of novel miRNAs class I identified. The maize mature miRNA (green), miRNA* (pink) were illustrated in pre-miRNA with chromosome and locus information based in the maize genome v.2. All novel miRNA sequences were denominated Zma_miR_Seq following the number, varying from 01 to 15. (PDF 413 KB) [file 12864_2014_6444_MOESM3_ESM.pdf]
