# Supplementary material for: Genome-wide identification of microRNA and siRNA responsive to endophytic beneficial diazotrophic bacteria in maize
Source: BMC Genomics. 2014 Sep 6;15(1):766. doi: 10.1186/1471-2164-15-766 (PMC4168055; doi:10.1186/1471-2164-15-766)
Supplement: Supplementary file 4 — Additional file 4: Figure S4: CViT image of the B73 assembly aligned with precursor of novel miRNAs. The POPcorn website (http://popcorn.maizegdb.org/main/index.php) was used. All novel miRNA sequences were denominated Zma_miR_Seq following the number, varying from 01 to 15. (PDF 454 KB) [file 12864_2014_6444_MOESM4_ESM.pdf]

Additional file Figure S4: **CViT image of the B73 assembly aligned with precursor of novel miRNAs**. The POPcorn website (http://popcorn.maizegdb.org/main/index.php) was used. All novel miRNAs sequence were denominated Zma_miR_Seq following the number, varying 01 to 15.

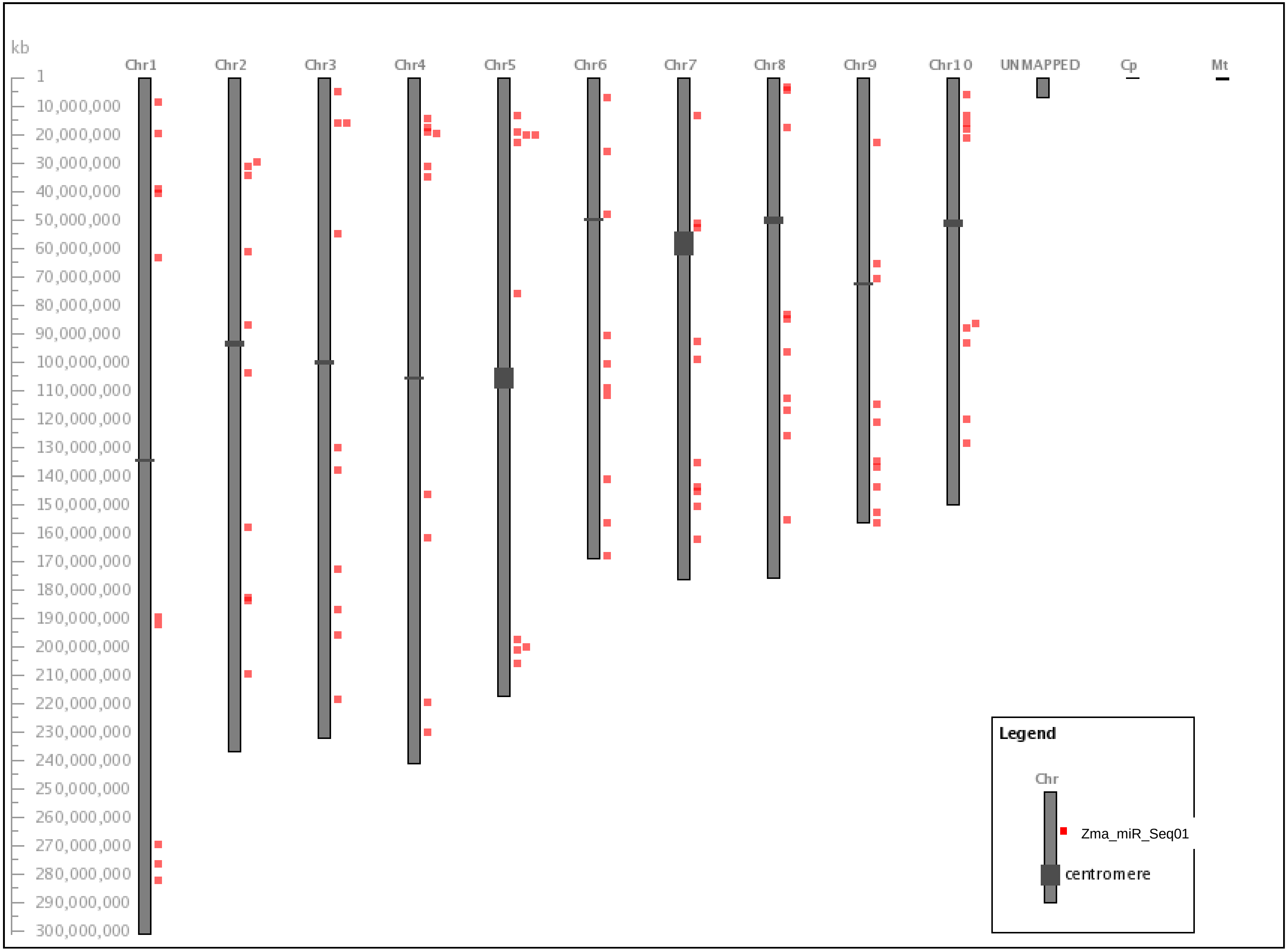

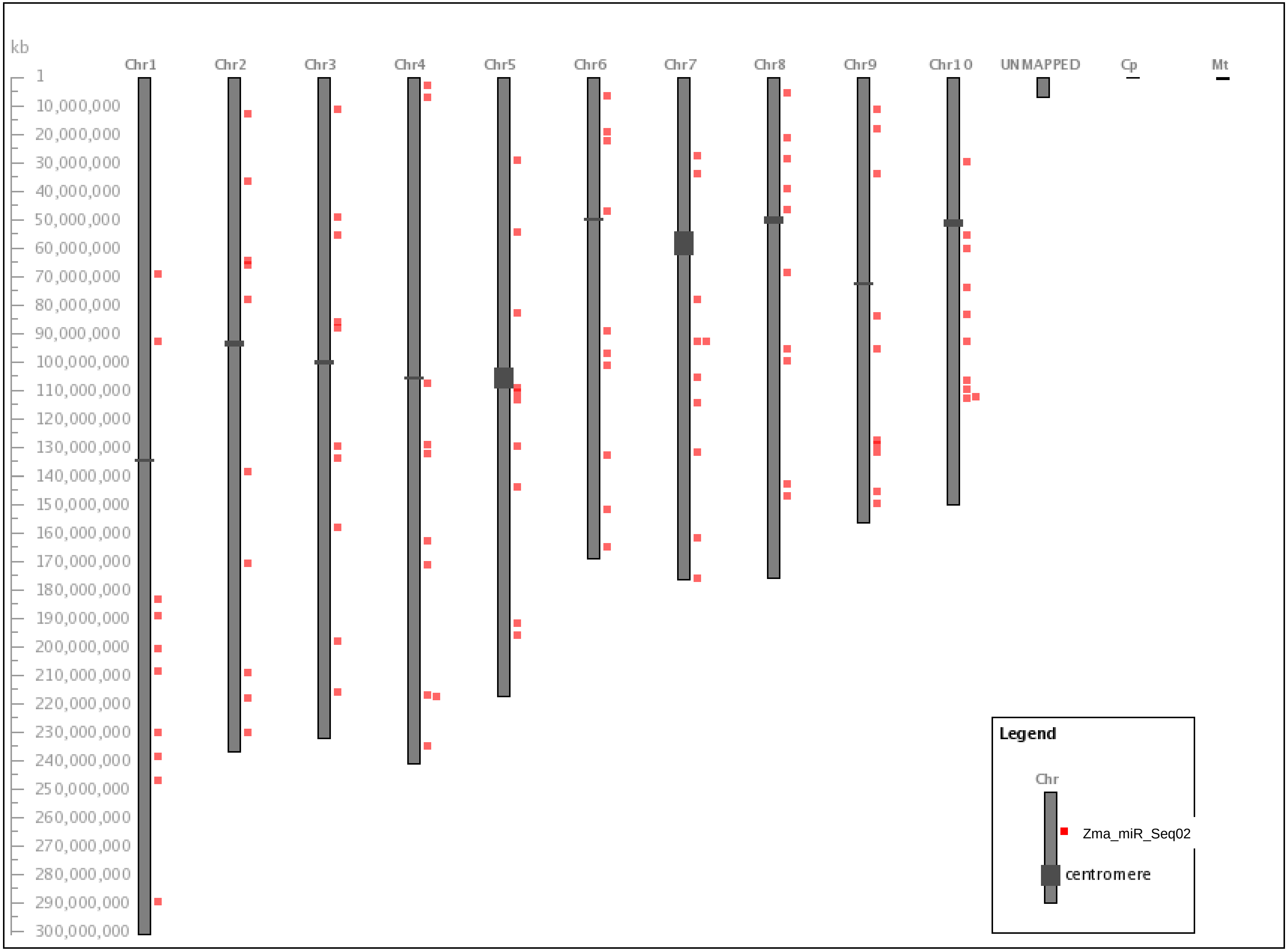

kb
1
10,000,000
20,000,000
30,000,000
40,000,000
50,000,000
60,000,000
70,000,000
80,000,000
90,000,000
100,000,000
110,000,000
120,000,000
130,000,000
140,000,000
150,000,000
160,000,000
170,000,000
180,000,000
190,000,000
200,000,000
210,000,000
220,000,000
230,000,000
240,000,000
250,000,000
260,000,000
270,000,000
280,000,000
290,000,000
300,000,000
Chr1
Chr2
Chr3
Chr4
Chr5
Chr6
Chr7
Chr8
Chr9
Chr10
UNMAPPED
Cp
Mt
Legend
Chr
Zma_miR_Seq02
centromere

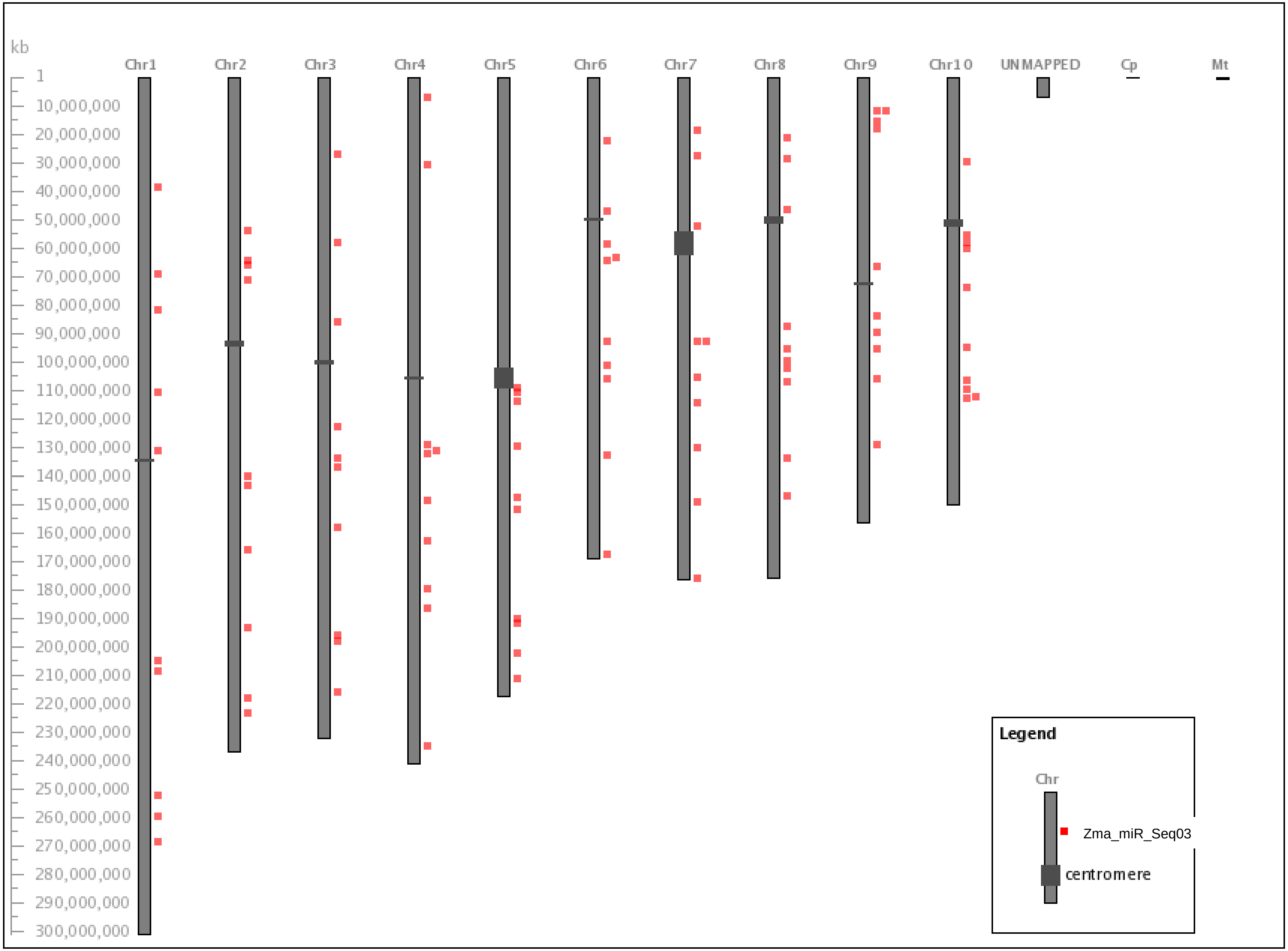
kb
1
10,000,000
20,000,000
30,000,000
40,000,000
50,000,000
60,000,000
70,000,000
80,000,000
90,000,000
100,000,000
110,000,000
120,000,000
130,000,000
140,000,000
150,000,000
160,000,000
170,000,000
180,000,000
190,000,000
200,000,000
210,000,000
220,000,000
230,000,000
240,000,000
250,000,000
260,000,000
270,000,000
280,000,000
290,000,000
300,000,000
Chr1
Chr2
Chr3
Chr4
Chr5
Chr6
Chr7
Chr8
Chr9
Chr10
UNMAPPED
Cp
Mt
Legend
Chr
Zma_miR_Seq03
centromere

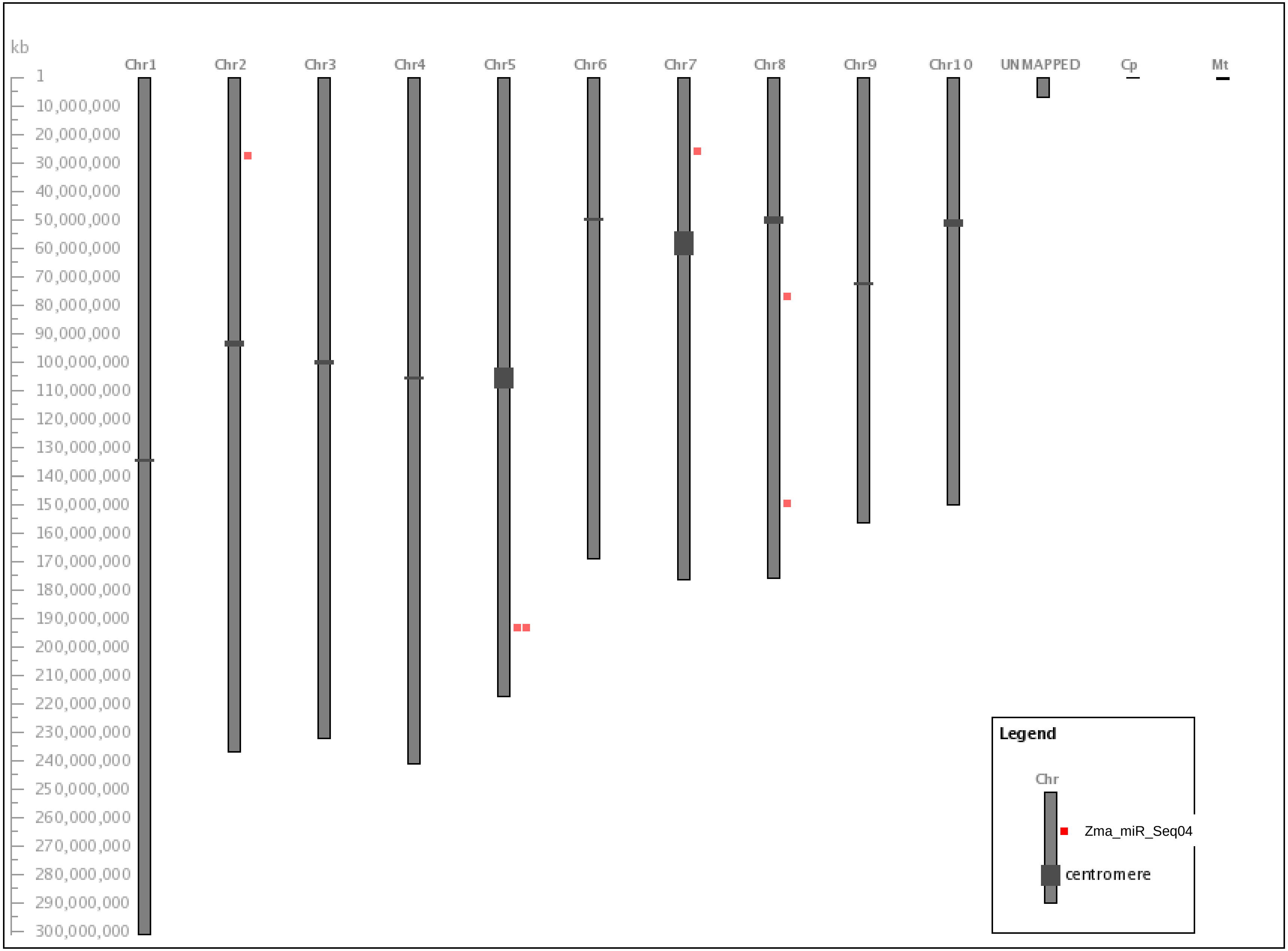
kb
Chr1
Chr2
Chr3
Chr4
Chr5
Chr6
Chr7
Chr8
Chr9
Chr10
UNMAPPED
Cp
Mt
1
10,000,000
20,000,000
30,000,000
40,000,000
50,000,000
60,000,000
70,000,000
80,000,000
90,000,000
100,000,000
110,000,000
120,000,000
130,000,000
140,000,000
150,000,000
160,000,000
170,000,000
180,000,000
190,000,000
200,000,000
210,000,000
220,000,000
230,000,000
240,000,000
250,000,000
260,000,000
270,000,000
280,000,000
290,000,000
300,000,000
Legend
Chr
Zma_miR_Seq04
centromere

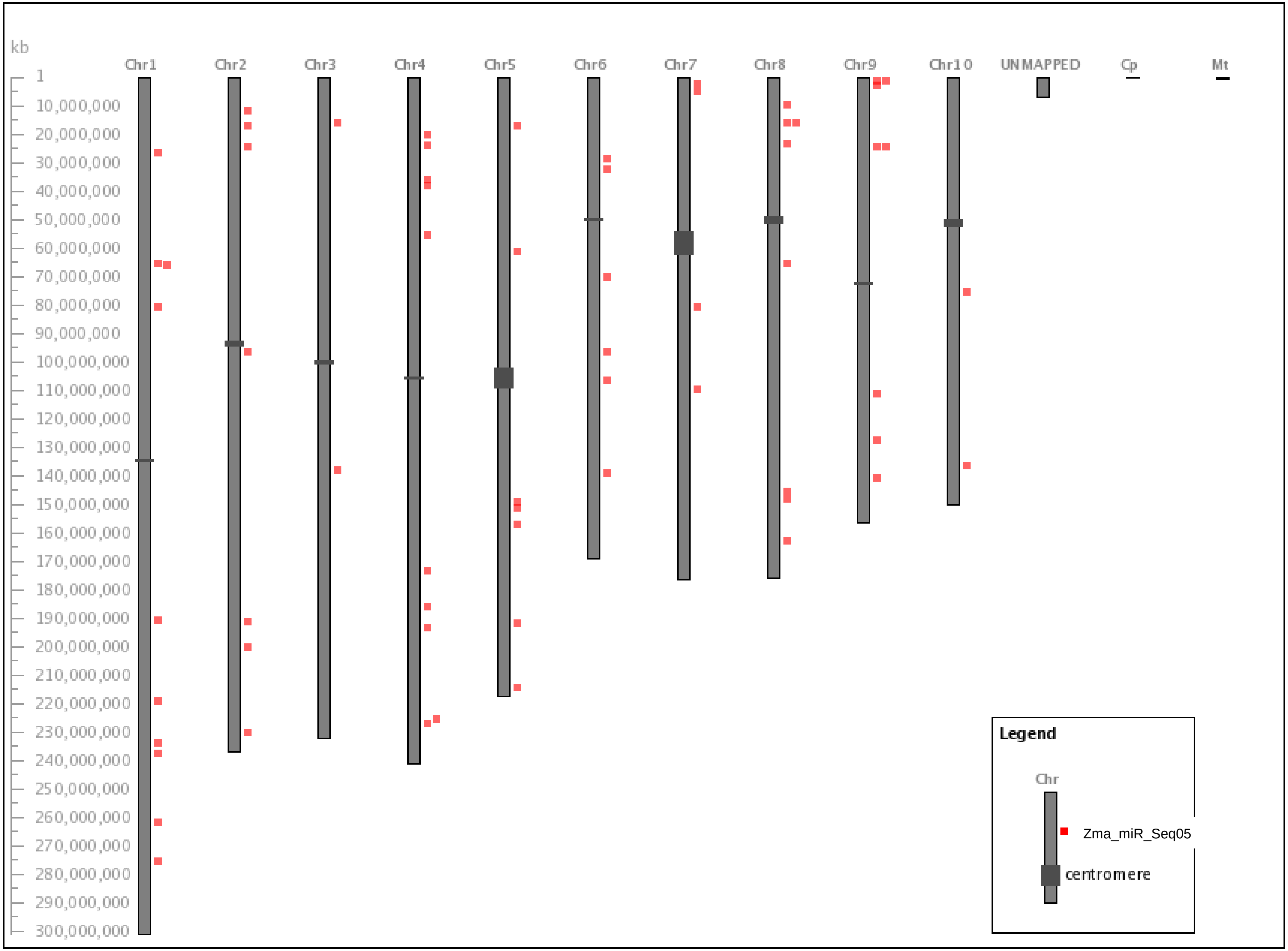
kb
1
10,000,000
20,000,000
30,000,000
40,000,000
50,000,000
60,000,000
70,000,000
80,000,000
90,000,000
100,000,000
110,000,000
120,000,000
130,000,000
140,000,000
150,000,000
160,000,000
170,000,000
180,000,000
190,000,000
200,000,000
210,000,000
220,000,000
230,000,000
240,000,000
250,000,000
260,000,000
270,000,000
280,000,000
290,000,000
300,000,000
Chr1
Chr2
Chr3
Chr4
Chr5
Chr6
Chr7
Chr8
Chr9
Chr10
UNMAPPED
Cp
Mt
Legend
Chr
Zma_miR_Seq05
centromere

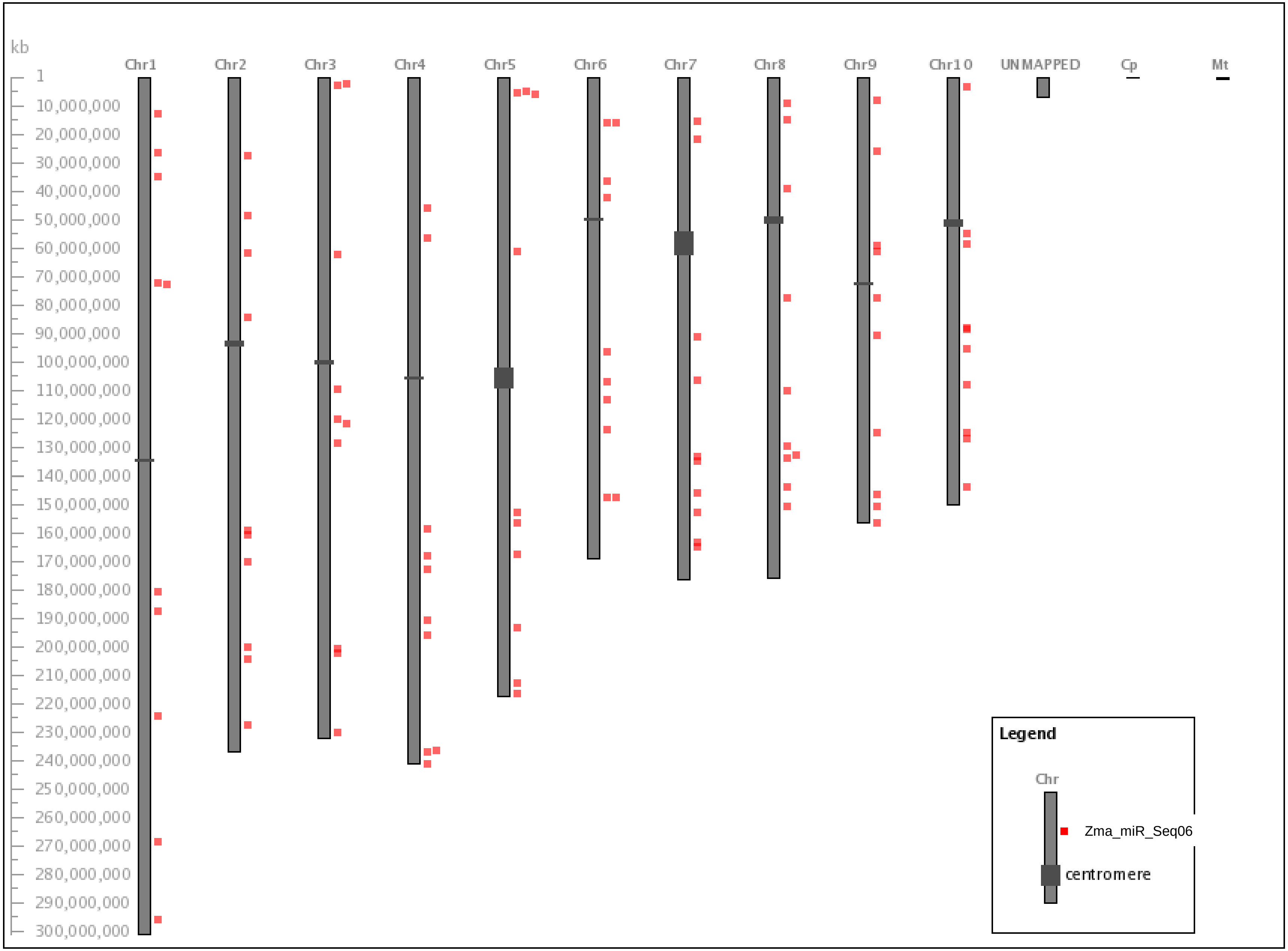
kb
Chr1
Chr2
Chr3
Chr4
Chr5
Chr6
Chr7
Chr8
Chr9
Chr10
UNMAPPED
Cp
Mt
1
10,000,000
20,000,000
30,000,000
40,000,000
50,000,000
60,000,000
70,000,000
80,000,000
90,000,000
100,000,000
110,000,000
120,000,000
130,000,000
140,000,000
150,000,000
160,000,000
170,000,000
180,000,000
190,000,000
200,000,000
210,000,000
220,000,000
230,000,000
240,000,000
250,000,000
260,000,000
270,000,000
280,000,000
290,000,000
300,000,000
Legend
Chr
Zma_miR_Seq06
centromere

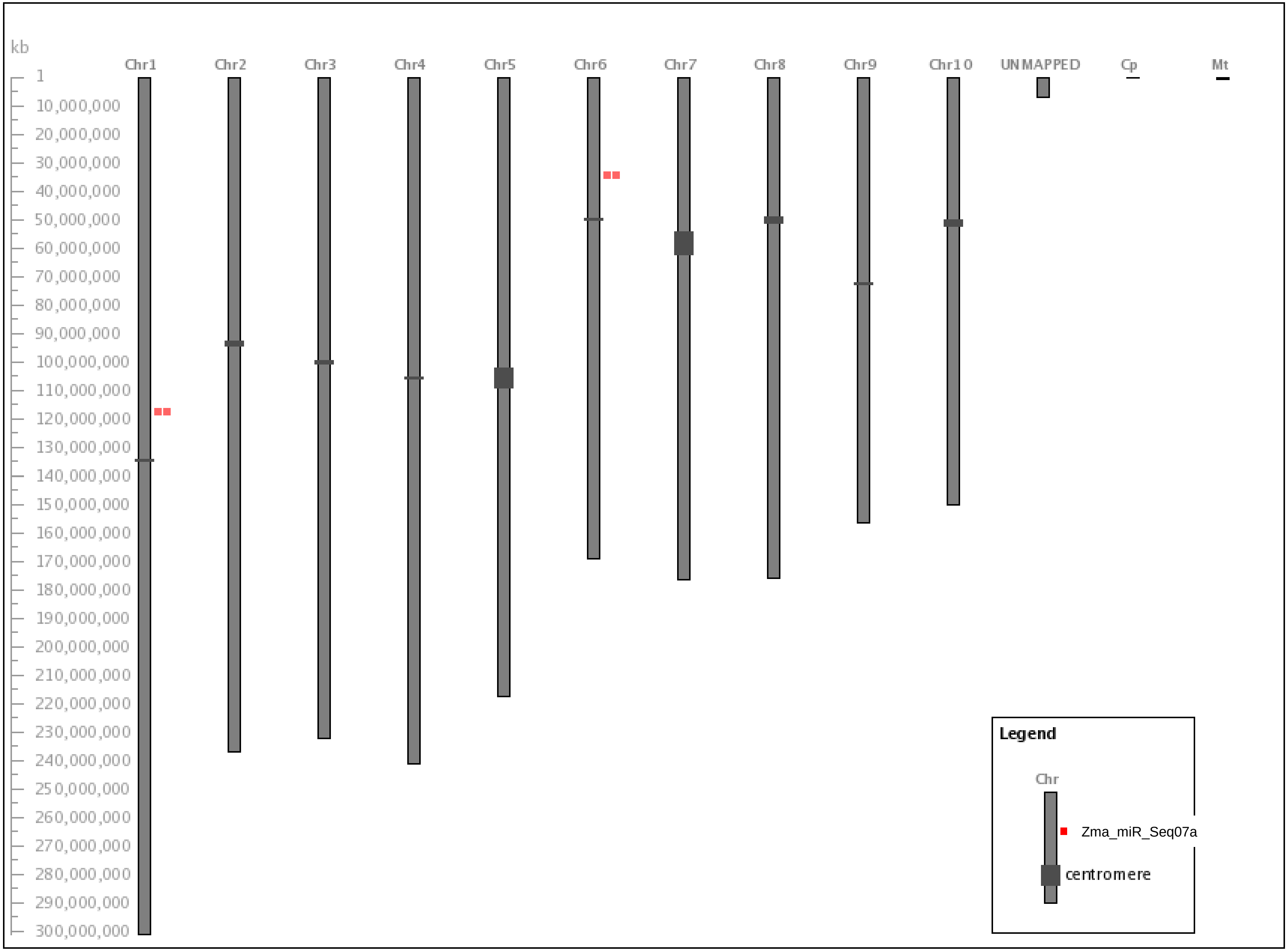
kb
1
10,000,000
20,000,000
30,000,000
40,000,000
50,000,000
60,000,000
70,000,000
80,000,000
90,000,000
100,000,000
110,000,000
120,000,000
130,000,000
140,000,000
150,000,000
160,000,000
170,000,000
180,000,000
190,000,000
200,000,000
210,000,000
220,000,000
230,000,000
240,000,000
250,000,000
260,000,000
270,000,000
280,000,000
290,000,000
300,000,000
Chr1
Chr2
Chr3
Chr4
Chr5
Chr6
Chr7
Chr8
Chr9
Chr10
UNMAPPED
Cp
Mt
Legend
Chr
Zma_miR_Seq07a
centromere

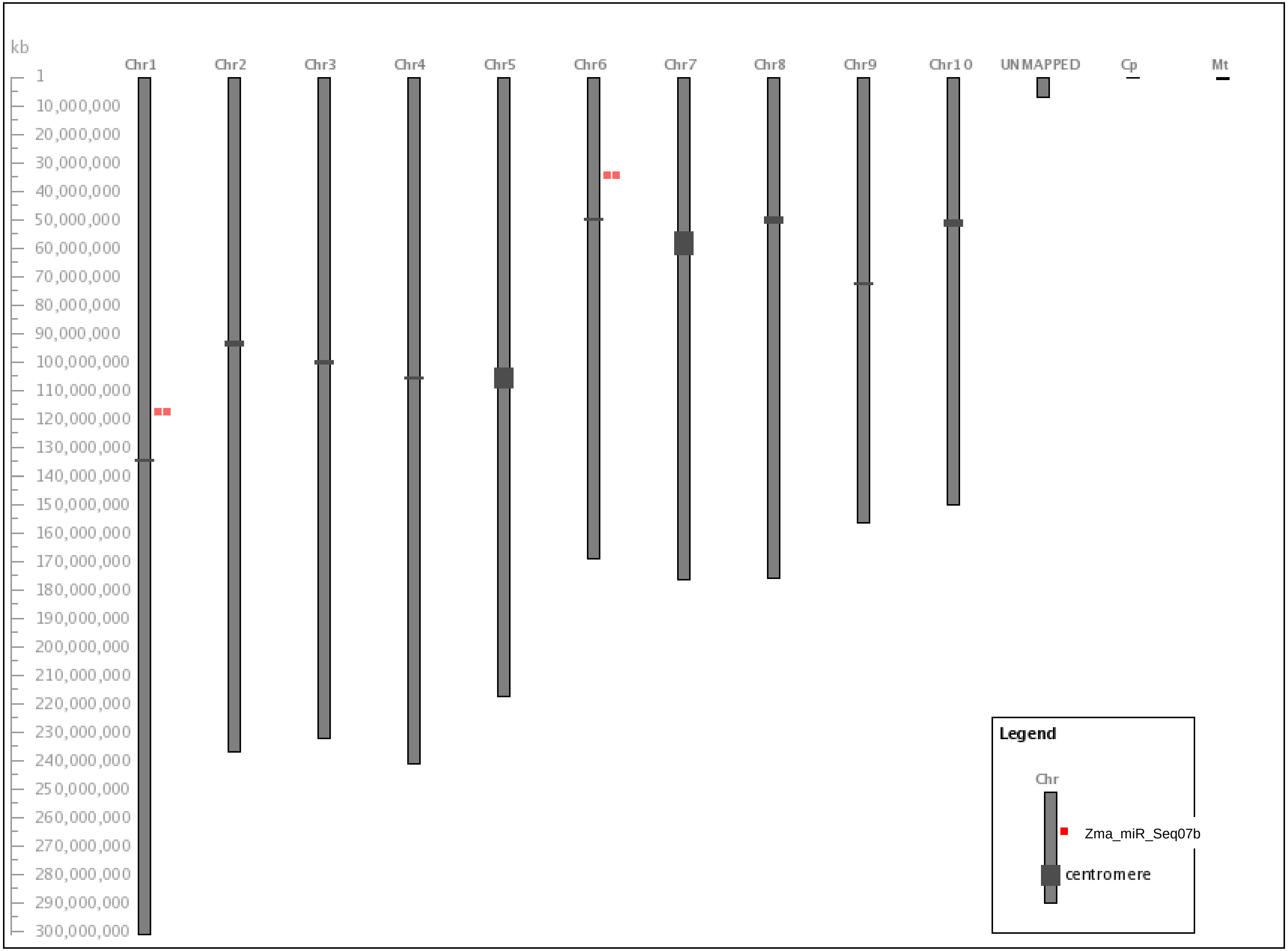

kb
Chr1
Chr2
Chr3
Chr4
Chr5
Chr6
Chr7
Chr8
Chr9
Chr10
UNMAPPED
Cp
Mt
1
10,000,000
20,000,000
30,000,000
40,000,000
50,000,000
60,000,000
70,000,000
80,000,000
90,000,000
100,000,000
110,000,000
120,000,000
130,000,000
140,000,000
150,000,000
160,000,000
170,000,000
180,000,000
190,000,000
200,000,000
210,000,000
220,000,000
230,000,000
240,000,000
250,000,000
260,000,000
270,000,000
280,000,000
290,000,000
300,000,000
Legend
Chr
Zma_miR_Seq07b
centromere

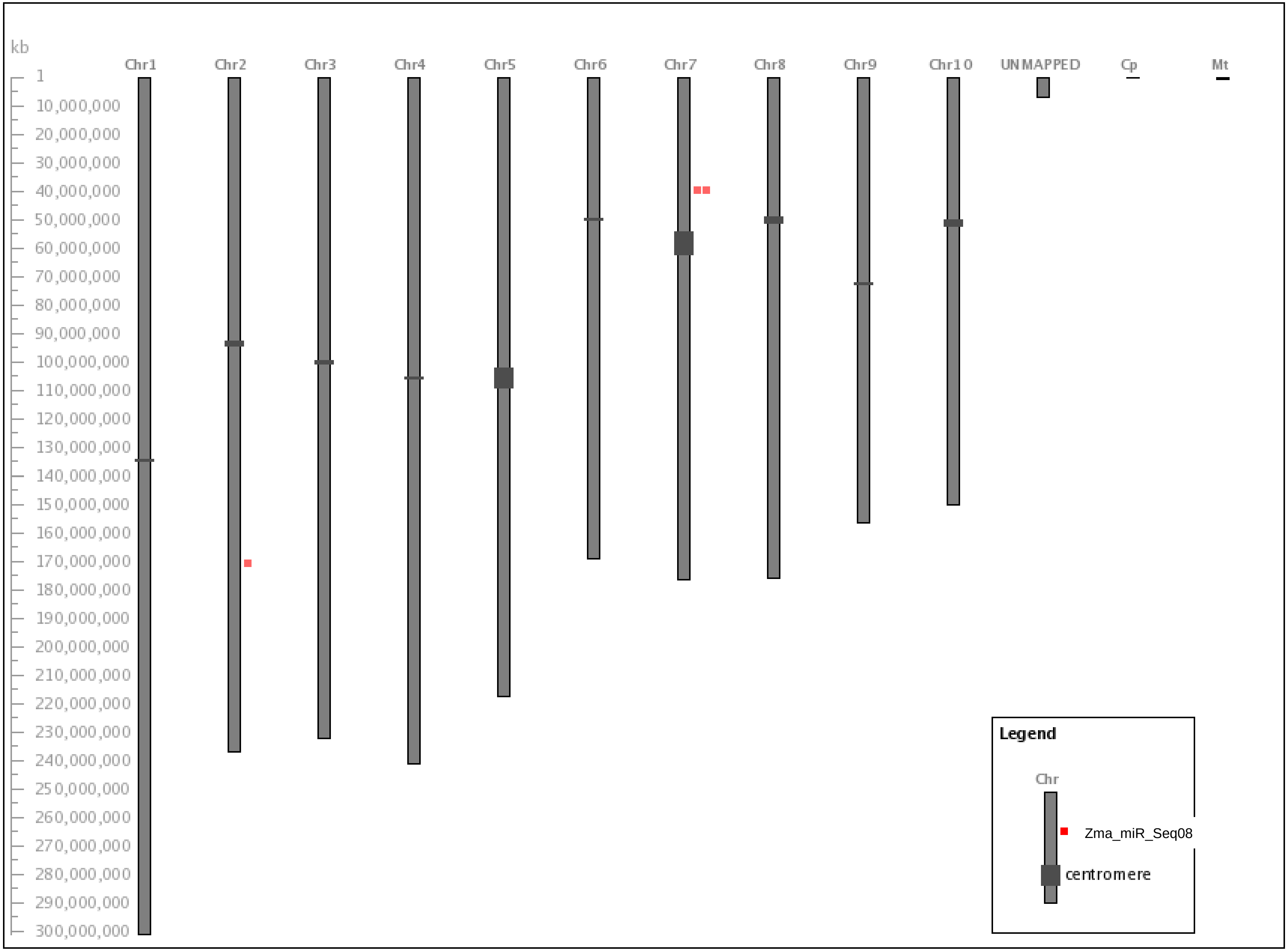
kb
Chr1
Chr2
Chr3
Chr4
Chr5
Chr6
Chr7
Chr8
Chr9
Chr10
UNMAPPED
Cp
Mt
1
10,000,000
20,000,000
30,000,000
40,000,000
50,000,000
60,000,000
70,000,000
80,000,000
90,000,000
100,000,000
110,000,000
120,000,000
130,000,000
140,000,000
150,000,000
160,000,000
170,000,000
180,000,000
190,000,000
200,000,000
210,000,000
220,000,000
230,000,000
240,000,000
250,000,000
260,000,000
270,000,000
280,000,000
290,000,000
300,000,000
Legend
Chr
Zma_miR_Seq08
centromere

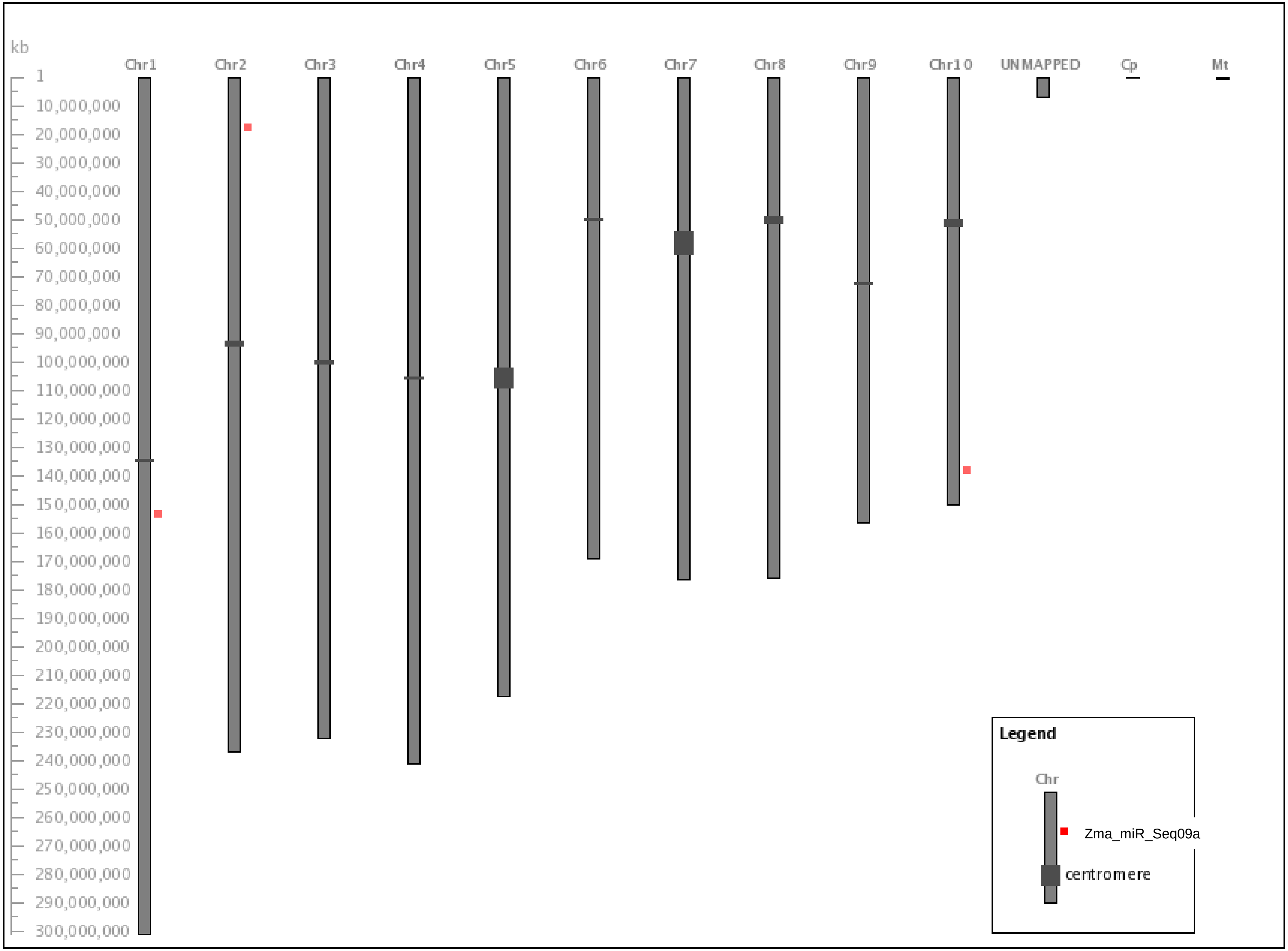

kb
1
10,000,000
20,000,000
30,000,000
40,000,000
50,000,000
60,000,000
70,000,000
80,000,000
90,000,000
100,000,000
110,000,000
120,000,000
130,000,000
140,000,000
150,000,000
160,000,000
170,000,000
180,000,000
190,000,000
200,000,000
210,000,000
220,000,000
230,000,000
240,000,000
250,000,000
260,000,000
270,000,000
280,000,000
290,000,000
300,000,000
Chr1
Chr2
Chr3
Chr4
Chr5
Chr6
Chr7
Chr8
Chr9
Chr10
UNMAPPED
Cp
Mt
Legend
Chr
Zma_miR_Seq09a
centromere

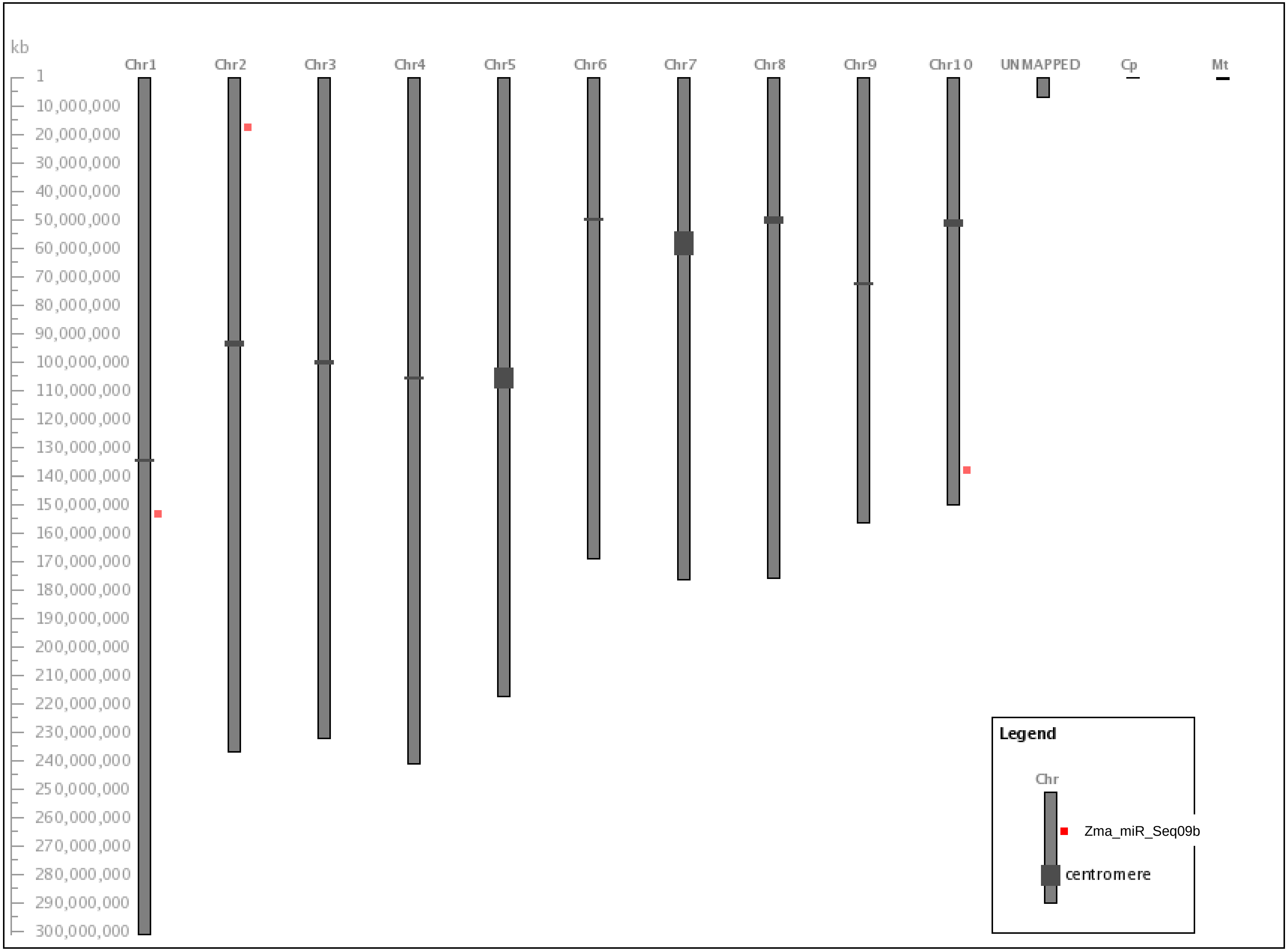
kb
Chr1
Chr2
Chr3
Chr4
Chr5
Chr6
Chr7
Chr8
Chr9
Chr10
UNMAPPED
Cp
Mt
1
10,000,000
20,000,000
30,000,000
40,000,000
50,000,000
60,000,000
70,000,000
80,000,000
90,000,000
100,000,000
110,000,000
120,000,000
130,000,000
140,000,000
150,000,000
160,000,000
170,000,000
180,000,000
190,000,000
200,000,000
210,000,000
220,000,000
230,000,000
240,000,000
250,000,000
260,000,000
270,000,000
280,000,000
290,000,000
300,000,000
Legend
Chr
Zma_miR_Seq09b
centromere

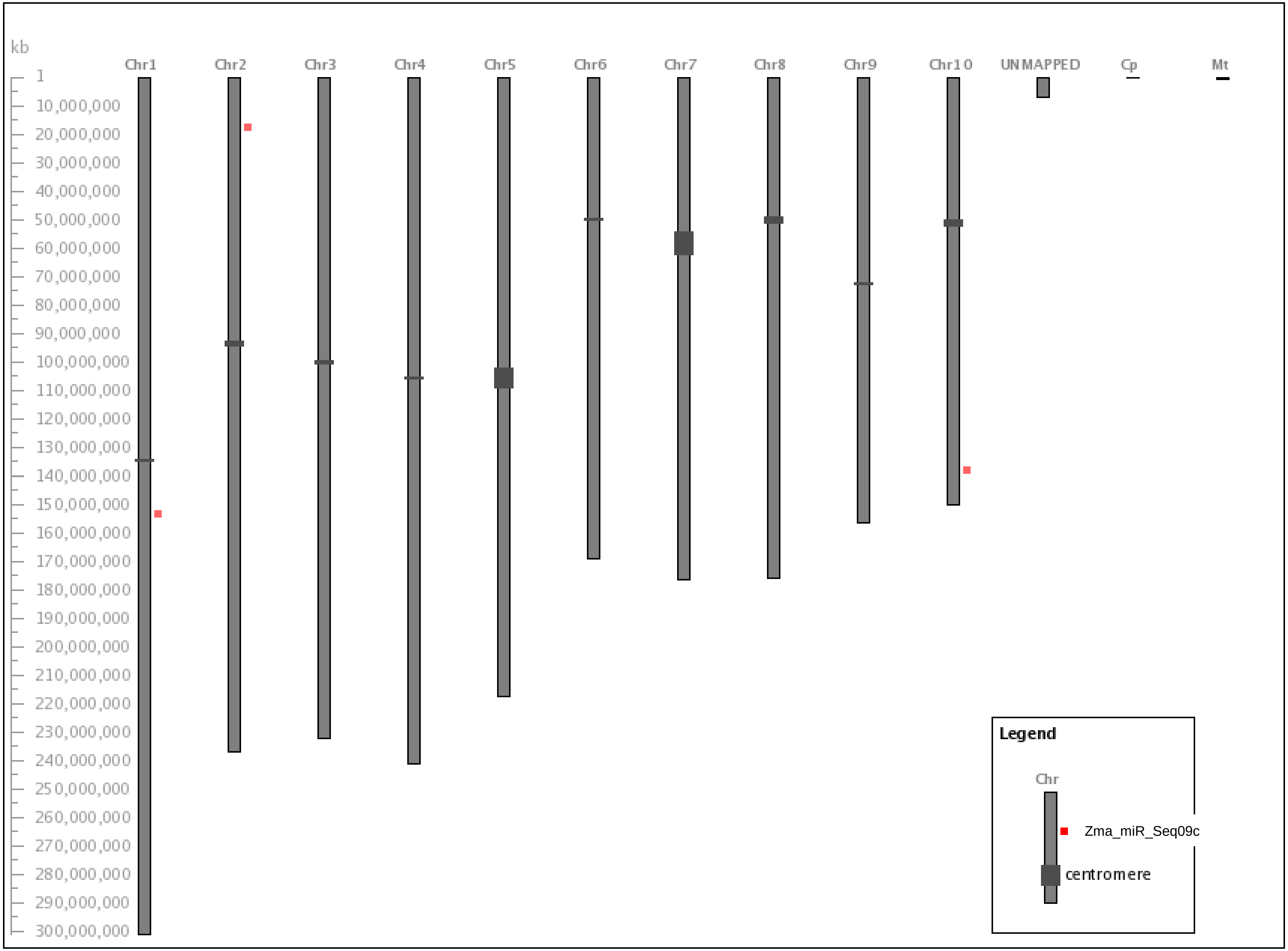

kb
Chr1
Chr2
Chr3
Chr4
Chr5
Chr6
Chr7
Chr8
Chr9
Chr10
UNMAPPED
Cp
Mt
1
10,000,000
20,000,000
30,000,000
40,000,000
50,000,000
60,000,000
70,000,000
80,000,000
90,000,000
100,000,000
110,000,000
120,000,000
130,000,000
140,000,000
150,000,000
160,000,000
170,000,000
180,000,000
190,000,000
200,000,000
210,000,000
220,000,000
230,000,000
240,000,000
250,000,000
260,000,000
270,000,000
280,000,000
290,000,000
300,000,000
Legend
Chr
Zma_miR_Seq09c
centromere

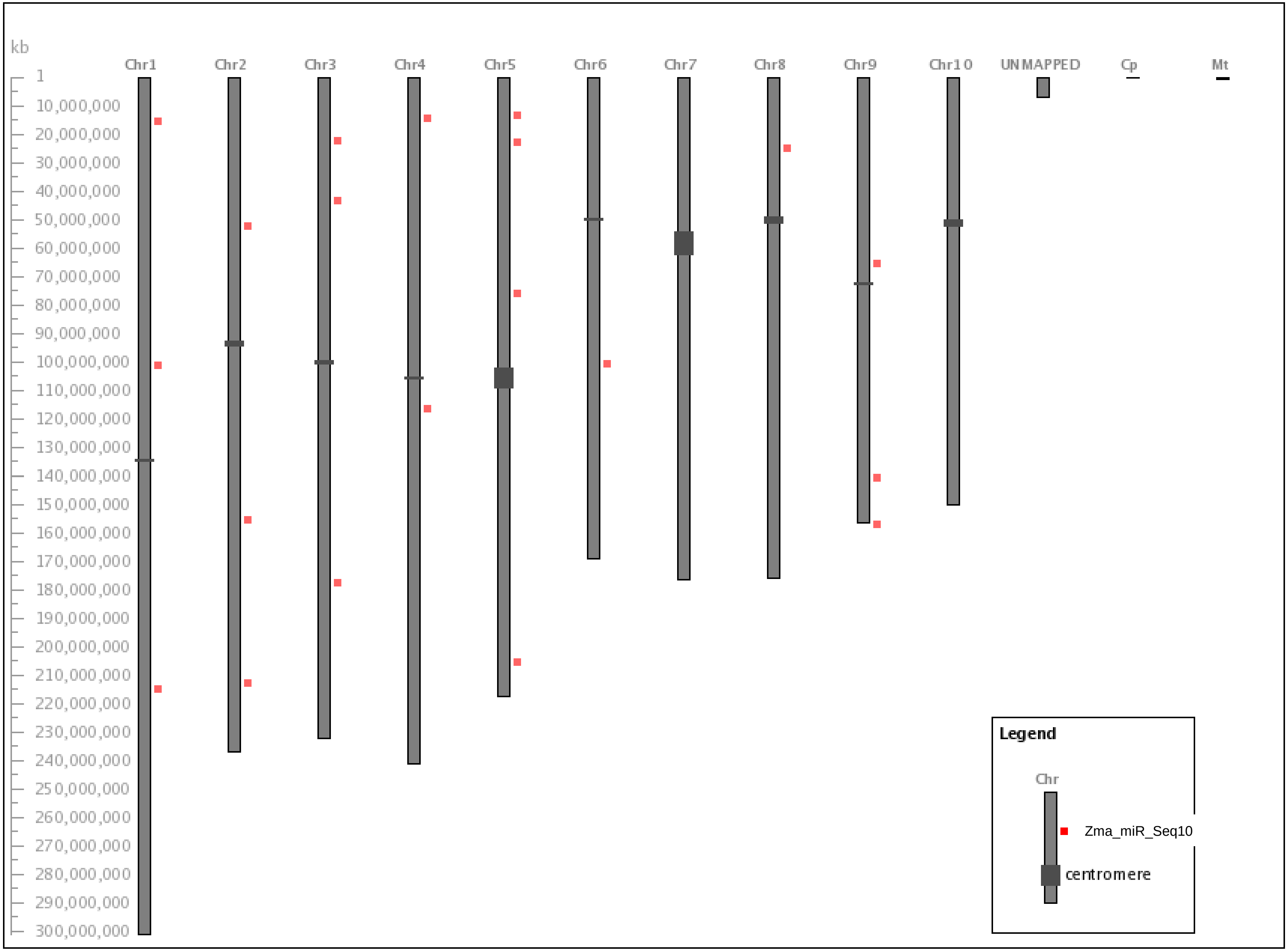

kb
1
10,000,000
20,000,000
30,000,000
40,000,000
50,000,000
60,000,000
70,000,000
80,000,000
90,000,000
100,000,000
110,000,000
120,000,000
130,000,000
140,000,000
150,000,000
160,000,000
170,000,000
180,000,000
190,000,000
200,000,000
210,000,000
220,000,000
230,000,000
240,000,000
250,000,000
260,000,000
270,000,000
280,000,000
290,000,000
300,000,000
Chr1
Chr2
Chr3
Chr4
Chr5
Chr6
Chr7
Chr8
Chr9
Chr10
UNMAPPED
Cp
Mt
Legend
Chr
Zma_miR_Seq10
centromere

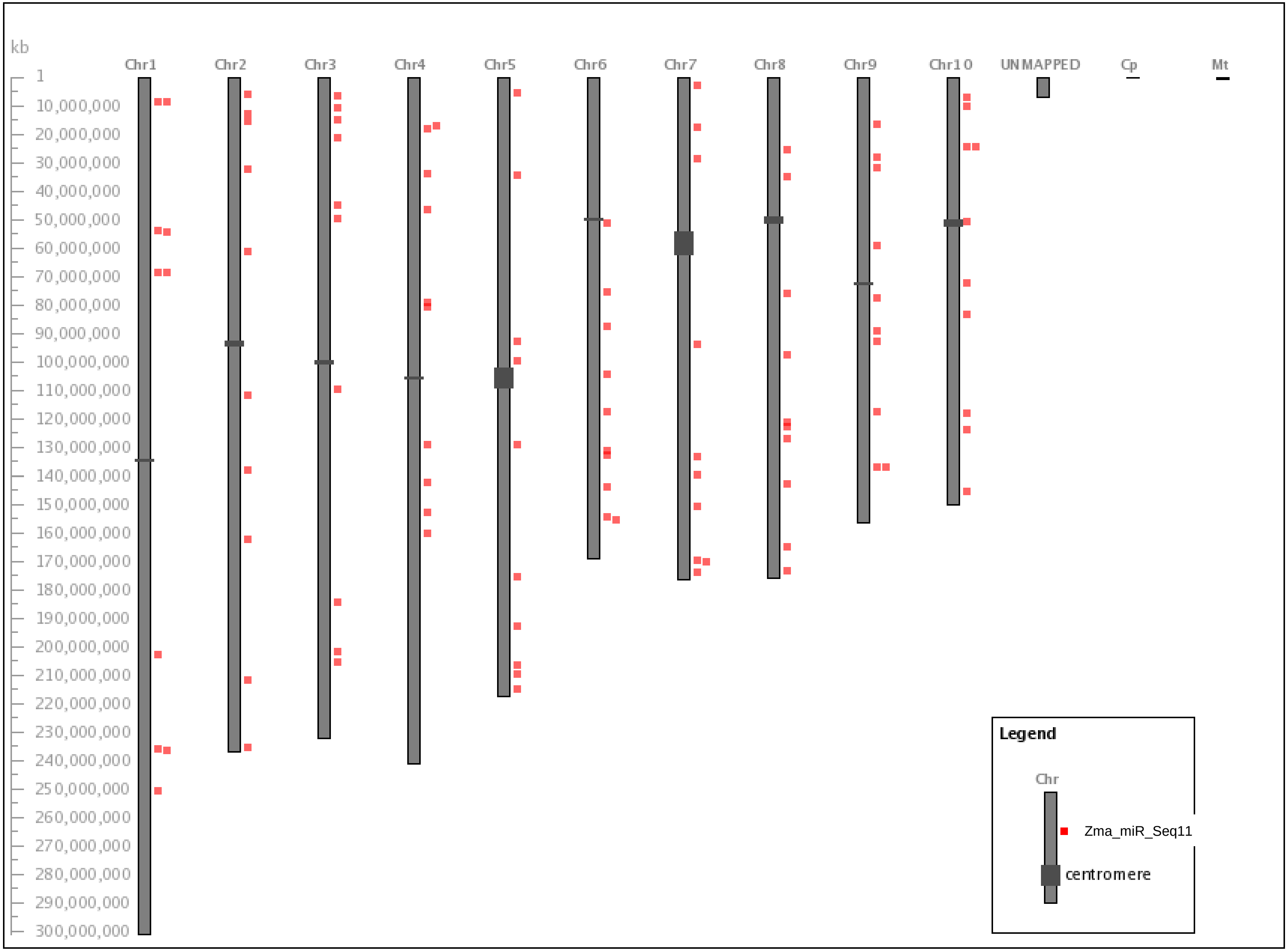
kb
Chr1
Chr2
Chr3
Chr4
Chr5
Chr6
Chr7
Chr8
Chr9
Chr10
UNMAPPED
Cp
Mt
1
10,000,000
20,000,000
30,000,000
40,000,000
50,000,000
60,000,000
70,000,000
80,000,000
90,000,000
100,000,000
110,000,000
120,000,000
130,000,000
140,000,000
150,000,000
160,000,000
170,000,000
180,000,000
190,000,000
200,000,000
210,000,000
220,000,000
230,000,000
240,000,000
250,000,000
260,000,000
270,000,000
280,000,000
290,000,000
300,000,000
Legend
Chr
Zma_miR_Seq11
centromere

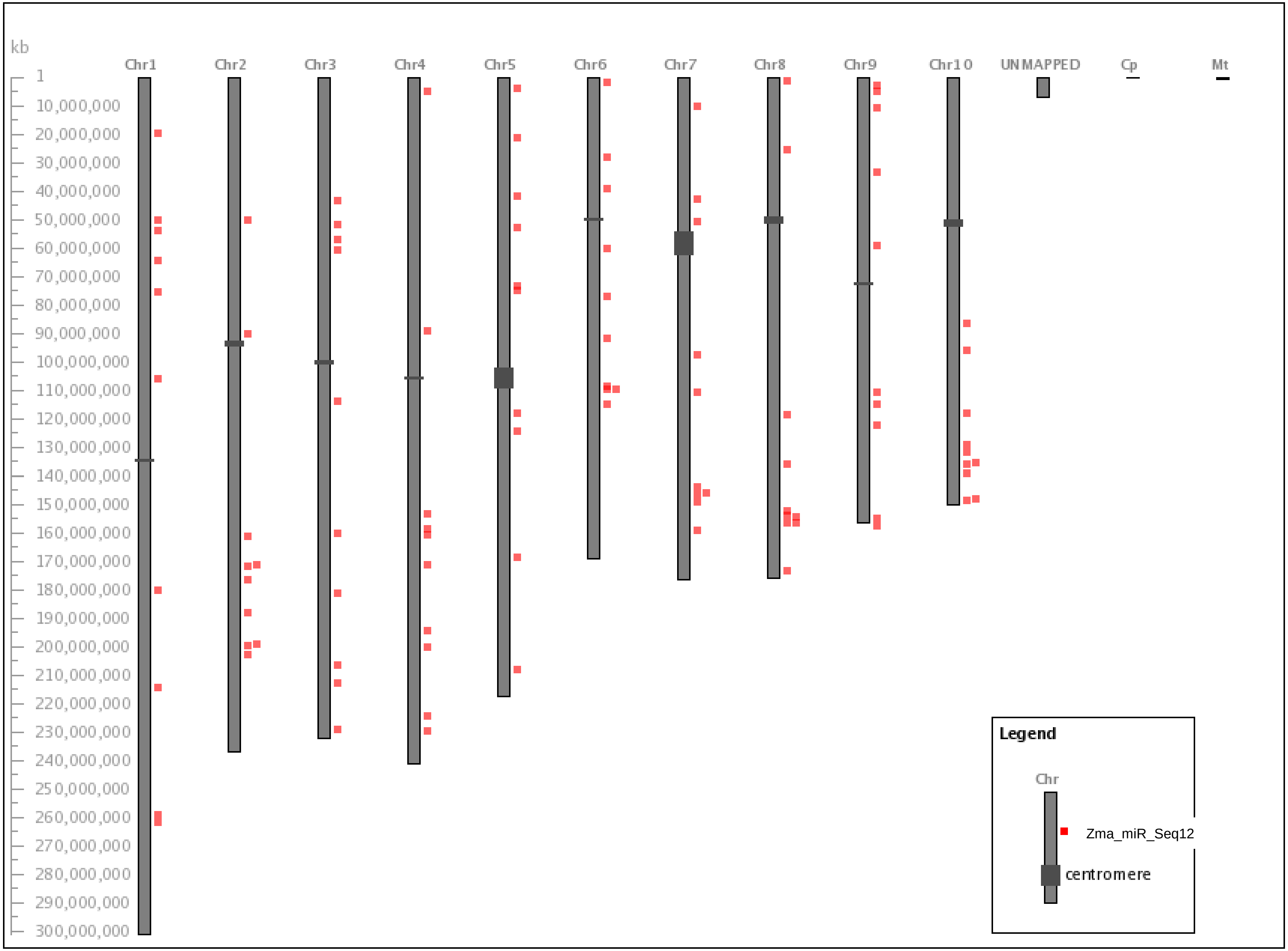
kb
1
10,000,000
20,000,000
30,000,000
40,000,000
50,000,000
60,000,000
70,000,000
80,000,000
90,000,000
100,000,000
110,000,000
120,000,000
130,000,000
140,000,000
150,000,000
160,000,000
170,000,000
180,000,000
190,000,000
200,000,000
210,000,000
220,000,000
230,000,000
240,000,000
250,000,000
260,000,000
270,000,000
280,000,000
290,000,000
300,000,000
Chr1
Chr2
Chr3
Chr4
Chr5
Chr6
Chr7
Chr8
Chr9
Chr10
UNMAPPED
Cp
Mt
Legend
Chr
Zma_miR_Seq12
centromere

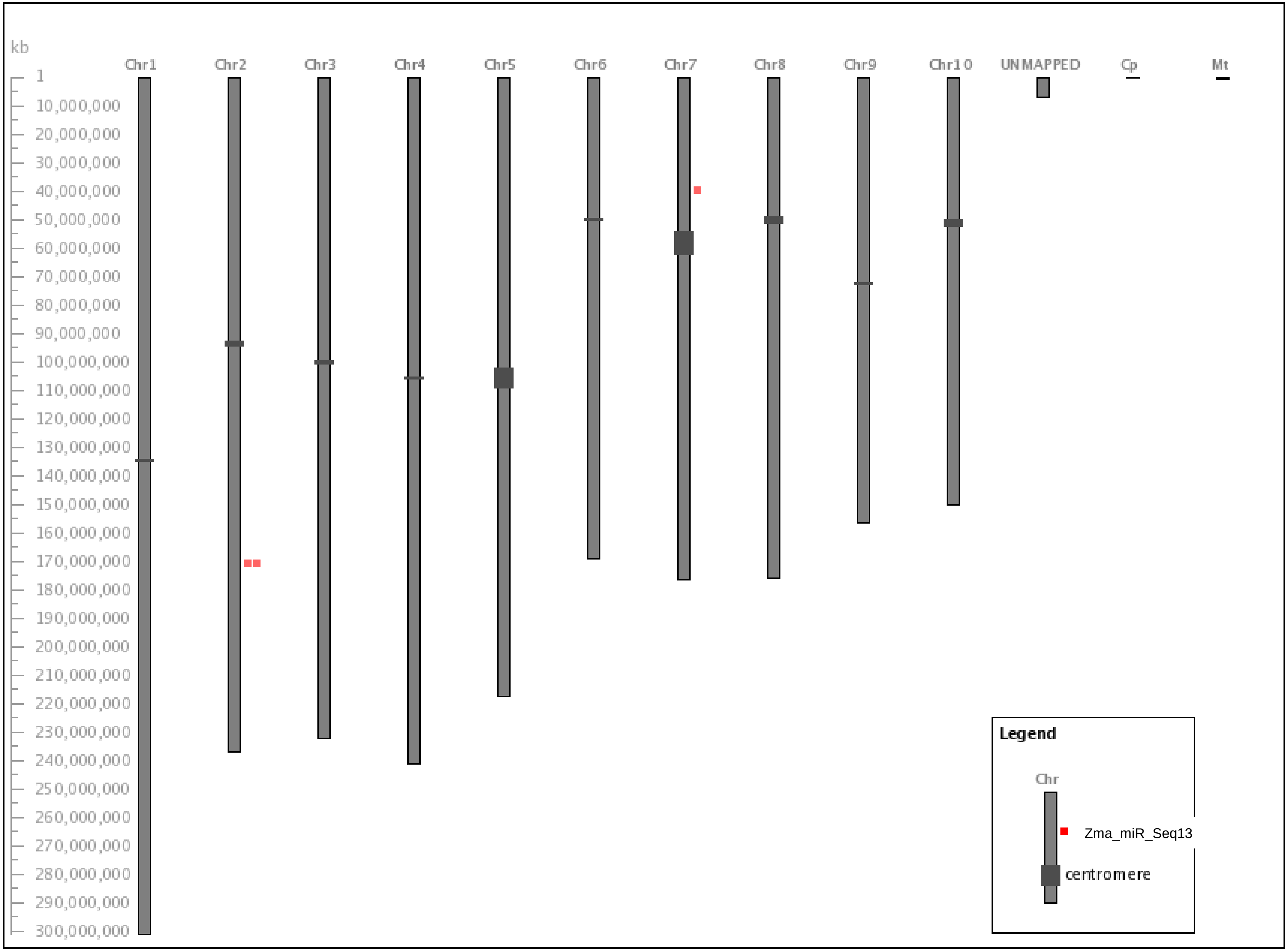

kb
1
10,000,000
20,000,000
30,000,000
40,000,000
50,000,000
60,000,000
70,000,000
80,000,000
90,000,000
100,000,000
110,000,000
120,000,000
130,000,000
140,000,000
150,000,000
160,000,000
170,000,000
180,000,000
190,000,000
200,000,000
210,000,000
220,000,000
230,000,000
240,000,000
250,000,000
260,000,000
270,000,000
280,000,000
290,000,000
300,000,000
Chr1
Chr2
Chr3
Chr4
Chr5
Chr6
Chr7
Chr8
Chr9
Chr10
UNMAPPED
Cp
Mt
Legend
Chr
Zma_miR_Seq13
centromere

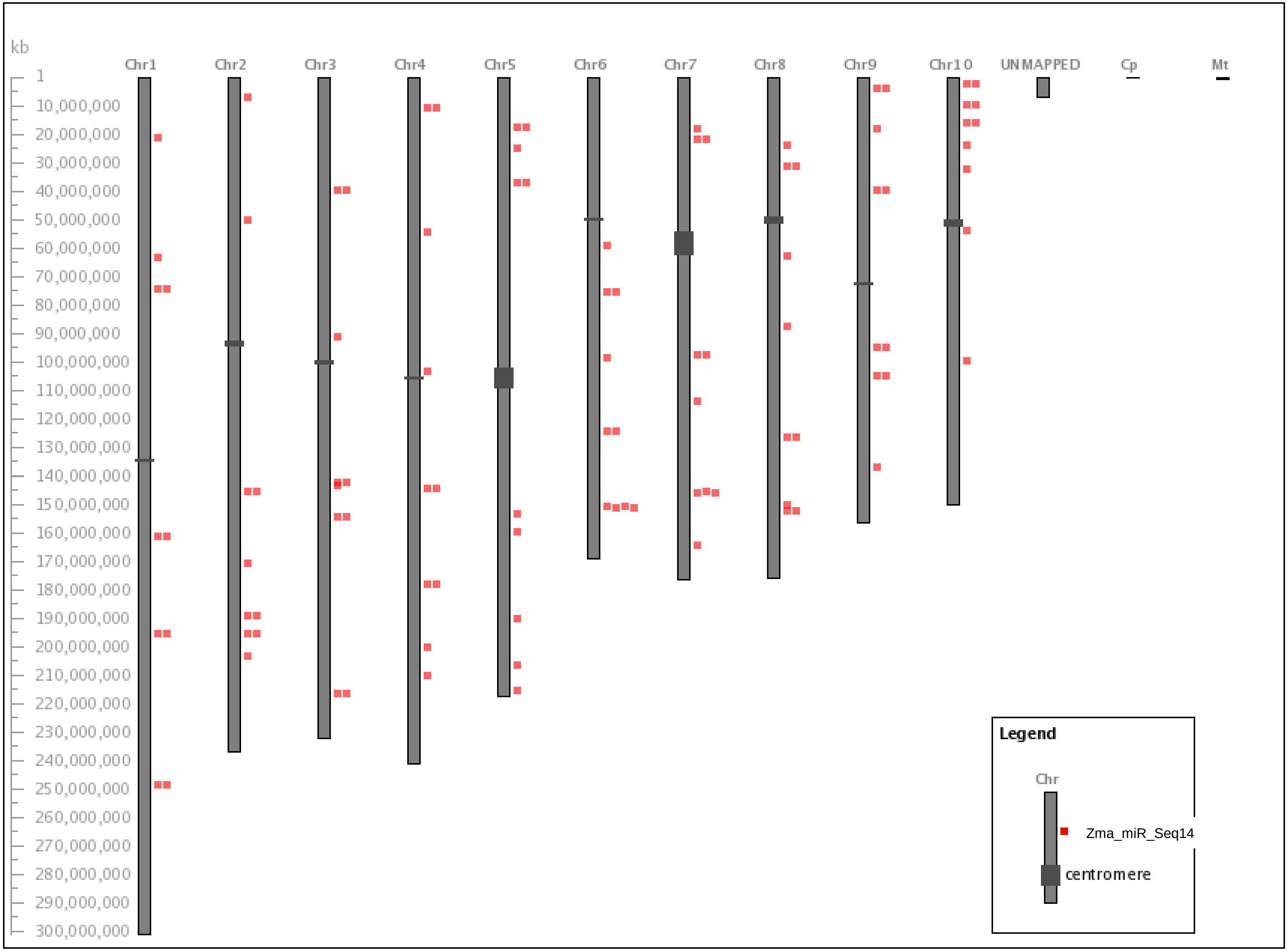
kb
Chr1
Chr2
Chr3
Chr4
Chr5
Chr6
Chr7
Chr8
Chr9
Chr10
UNMAPPED
Cp
Mt
1
10,000,000
20,000,000
30,000,000
40,000,000
50,000,000
60,000,000
70,000,000
80,000,000
90,000,000
100,000,000
110,000,000
120,000,000
130,000,000
140,000,000
150,000,000
160,000,000
170,000,000
180,000,000
190,000,000
200,000,000
210,000,000
220,000,000
230,000,000
240,000,000
250,000,000
260,000,000
270,000,000
280,000,000
290,000,000
300,000,000
Legend
Chr
Zma_miR_Seq14
centromere

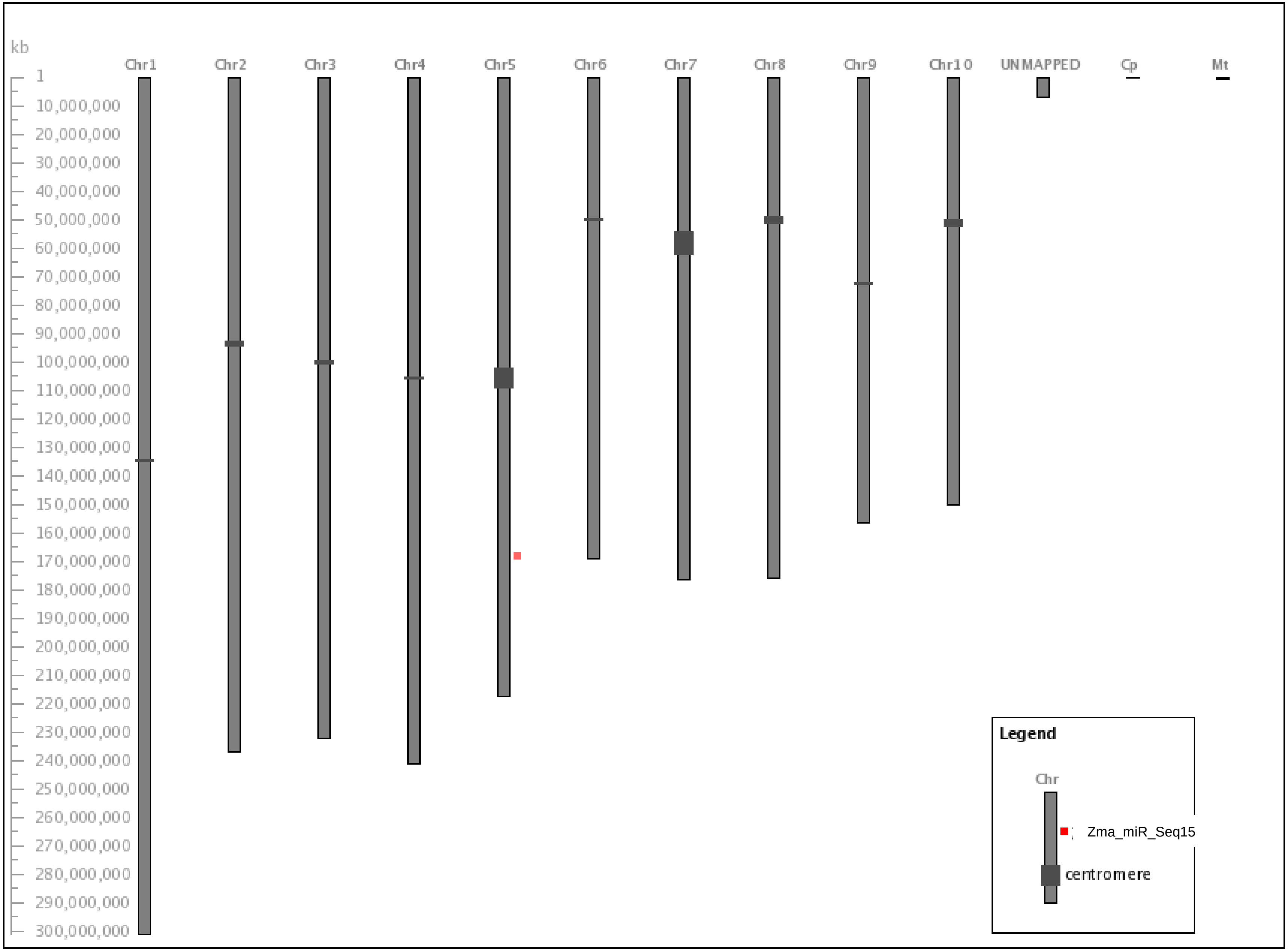
kb
1
10,000,000
20,000,000
30,000,000
40,000,000
50,000,000
60,000,000
70,000,000
80,000,000
90,000,000
100,000,000
110,000,000
120,000,000
130,000,000
140,000,000
150,000,000
160,000,000
170,000,000
180,000,000
190,000,000
200,000,000
210,000,000
220,000,000
230,000,000
240,000,000
250,000,000
260,000,000
270,000,000
280,000,000
290,000,000
300,000,000
Chr1
Chr2
Chr3
Chr4
Chr5
Chr6
Chr7
Chr8
Chr9
Chr10
UNMAPPED
Cp
Mt
Legend
Chr
Zma_miR_Seq15
centromere
