# Supplementary material for: Genome-wide identification of microRNA and siRNA responsive to endophytic beneficial diazotrophic bacteria in maize
Source: BMC Genomics. 2014 Sep 6;15(1):766. doi: 10.1186/1471-2164-15-766 (PMC4168055; doi:10.1186/1471-2164-15-766)

Additional file Figure S5: **Analysis of siRNA and methylation profile at the splice variant of GRMZM2G487629 gene.** (A) Localization of GRMZM2G487629 in chromosome 1 and two possible splice variant from these gene (boxes represent exons, and lines are intron). (B) Position of siRNA aligned in GRMZM2G487629_T02. The two peaks, blue and green, are siRNA non-redundant sequences from inoculated and control libraries, respectively. (C) Four data tracks show methylation levels for B73 in different sequence contexts. Methylation levels are displayed on a scale from 0 to 1.

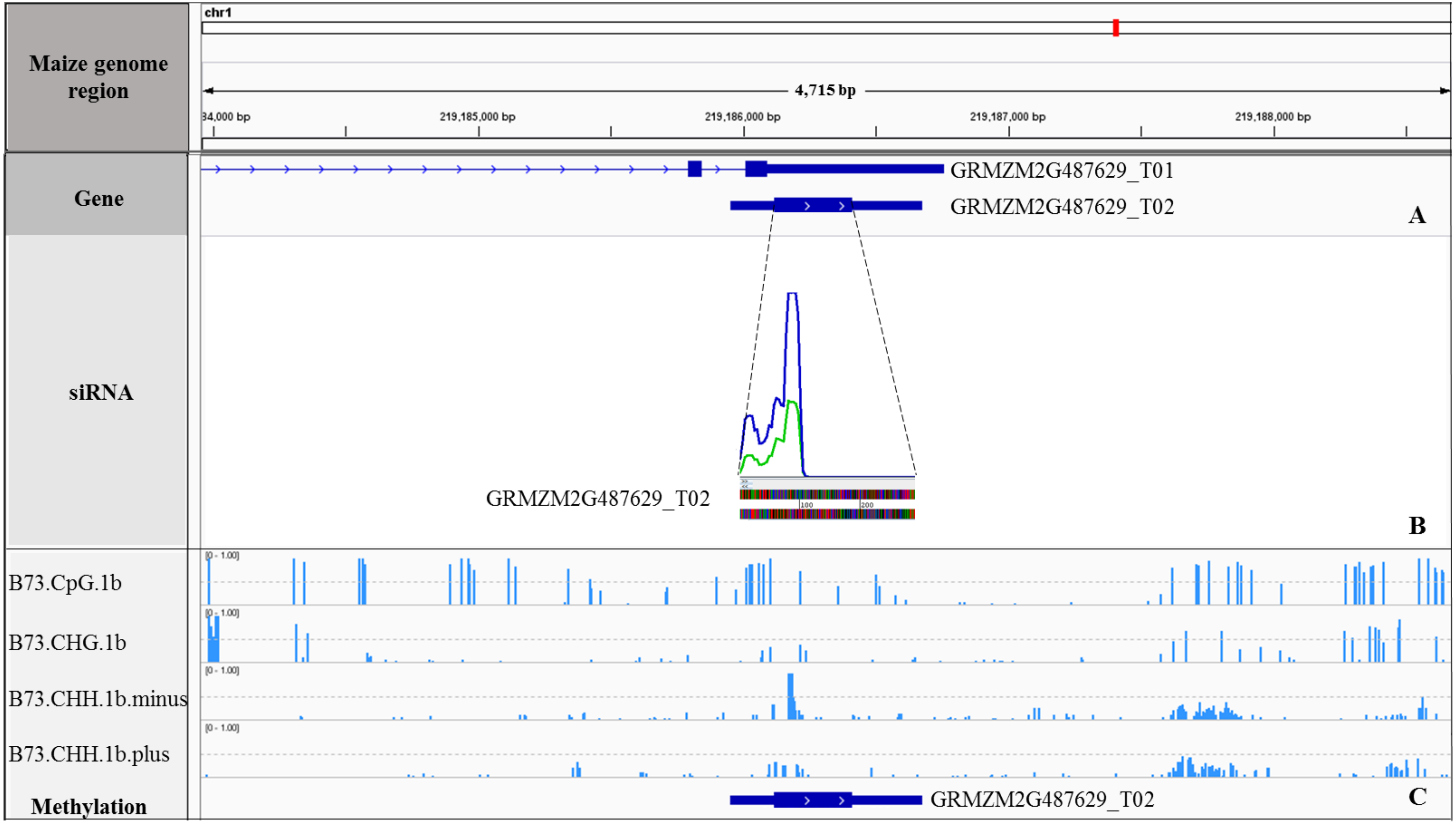

Supplement: Supplementary file 5 — Additional file 5: Figure S5: Analysis of siRNA and methylation profile at the splice variant of GRMZM2G487629 gene. (A) Localization of GRMZM2G487629 in chromosome 1 and two possible splice variants from this gene (boxes represent exons, and lines are introns). (B) Position of siRNA aligned in GRMZM2G487629_T02. The two peaks, blue and green, are siRNA non-redundant sequences from inoculated and control libraries, respectively. (C) Four data tracks show methylation levels for B73 in different sequence contexts. Methylation levels are displayed on a scale from 0 to 1. (PDF 115 KB) [file 12864_2014_6444_MOESM5_ESM.pdf]
